# Molecular mechanisms and regulation of inflammasome activation and signaling: sensing of pathogens and damage molecular patterns

**DOI:** 10.1038/s41423-025-01354-y

**Published:** 2025-10-09

**Authors:** Sagar R. Dubey, Cynthia Turnbull, Abhimanu Pandey, Anyang Zhao, Melan Kurera, Radhwan Al-Zidan, Cheng Shen, Manjul Gautam, Shreya Mahajan, Poonam S. Jadhav, Aritra Ghosh, Chinh Ngo, Si Ming Man

**Affiliations:** Division of Immunology and Infectious Diseases, The John Curtin School of Medical Research, The Australian National University, Canberra, ACT Australia

**Keywords:** GSDMD, Infection, Interferons, LPS, NINJ1, Pattern-recognition receptors, NOD-like receptors, Inflammasome

## Abstract

The inflammasome is an inflammatory signaling protein complex comprising a sensor protein, the adaptor protein ASC, and the cysteine protease caspase-1. Inflammasome sensor proteins are activated by microbial molecular patterns, endogenous self-derived damage signals, or exogenous environmental danger signals. Multiple inflammasomes that differ in their mechanisms of action and structural composition have been identified. The best characterized are the canonical NLRP1, NLRP3, NAIP-NLRC4, AIM2, and Pyrin inflammasomes and the noncanonical inflammasomes activated by caspase-4, caspase-5 or caspase-11. The lesser known inflammasomes are the NLRP6, NLRP7, NLRP9, NLRP10, NLRP12, CARD8, and MxA inflammasomes. Following inflammasome assembly, caspase-1 promotes the secretion of the proinflammatory cytokines IL-1β and IL-18, and pyroptosis is mediated by the membrane-disrupting proteins gasdermin D and ninjurin-1. These functional activities control innate and adaptive immune responses and the initiation, development, and progression of autoinflammation, cancer, infectious diseases, and neurodegenerative diseases. Understanding how inflammasomes respond to pathogens and sterile signals has refined our view of innate immunity and offered new therapeutic targets. In this review, we present a comprehensive overview of inflammasomes with an emphasis on the mechanistic principles that govern inflammasome formation. We also discuss the contributions of inflammasome activation to health and disease.

## Introduction

The inflammasome is a cytoplasmic protein complex that contains an inflammasome sensor protein, the adaptor protein called apoptosis-associated speck-like protein containing a CARD (known as ASC or PYCARD), and the cysteine protease caspase-1 [1–3]. Following the formation of this complex, caspase-1 undergoes activation and drives proteolytic cleavage or processing of a range of substrates. Among these substrates are pro-interleukin-1β (IL-1β) and pro-IL-18, which, via caspase-1-dependent cleavage, are converted to their biologically active forms [4, 5]. Caspase-1 also cleaves the pore-forming protein gasdermin D (GSDMD) [6–12]. Once cleaved, the N-terminal fragment of GSDMD forms pores in the plasma membrane, allowing the bioactive forms of IL-1β and IL-18 to escape from within the cytoplasm to outside of the cell [13, 14]. The build-up of GSDMD pores on the plasma membrane creates osmosis and the influx of water into the cell, causing the typical ballooning morphology and eventual lysis of the cell, called pyroptosis [15–18]. The physical rupture of the plasma cell membrane requires the membrane protein ninjurin-1 (also known as NINJ1) [19], and this tearing process liberates the remaining cellular content into the extracellular environment [20, 21].

Inflammasome formation is initiated by inflammasome sensor proteins. These sensor proteins are part of a larger family of germline-encoded pattern-recognition receptors (PRRs) that control inflammation, cell death, and the activation and recruitment of immune cells, resulting in hallmarks of inflammation characterized by redness, swelling, heat, and pain [22, 23]. PRRs, including inflammasome sensor proteins, detect all types of pathogen-associated molecular patterns (PAMPs), damage-associated molecular patterns (DAMPs), and exogenous environmental danger signals [24, 25]. All microbial components, including LPS, bacterial toxins, and viral proteins and nucleic acids, are considered PAMPs. DAMPs are endogenous self-derived molecules, such as nuclear and mitochondrial DNA and ATP, and when they are mislocalized, they are sensed by PRRs. Exogenous environmental danger signals are broadly defined and can include pollutants, silica, asbestos, and venom.

Several families of PRRs can form inflammasomes. The nucleotide-binding domain and leucine-rich repeat (LRR)-containing gene family (also known as NOD-like receptors or NLRs) carry the largest number of inflammasome-forming proteins. NLRP1, NLRP3, NLRC4, NLRP6, NLRP7, NLRP9, NLRP10, NLRP11, NLRP12, and NAIPs can form inflammasomes. Many NLRs contain a centrally located NACHT (also known as the domain present in NAIP, CIITA, HET-E, and TP-1; SPRY, Spla/Ryanodine receptor domain) and a C-terminal LRR. In general, NLRs carrying an N-terminal caspase activation and recruitment domain (CARD) are called NLRC, whereas those carrying an N-terminal Pyrin domain (PYD) are called NLRP [26–28].

Five other groups of PRRs can form inflammasomes. AIM2 and interferon gamma-inducible protein 16 (IFI16) from AIM2-like receptors (ALRs) are inflammasome sensor proteins. Pyrin is the only inflammasome sensor from the tripartite motif-containing protein receptor (TRIM). CARD8 from a family of loosely classified CARD-containing proteins and MxA from the interferon-inducible GTPase family are both poorly characterized proteins that have been shown to assemble inflammasome complexes. The last group of PRRs that can form inflammasomes is human caspase-4, human caspase-5, and mouse caspase-11. They are collectively referred to as noncanonical inflammasomes because they directly sense cytoplasmic LPS and subsequently drive the activation of the NLRP3 inflammasome [29, 30]. This unique activating step is referred to as the noncanonical inflammasome pathway. Since then, all other inflammasomes that do not require human caspase-4, human caspase-5, or mouse caspase-11 as part of their activation mechanisms have been known as canonical inflammasomes.

The discovery of new PRRs capable of initiating inflammasome activation via conventional or novel mechanisms has substantially advanced our understanding of innate immunity. Given the diverse range of signals that can drive inflammasome activation, aberrant or disrupted inflammasome signaling is linked to inflammatory diseases. As such, inflammasomes have emerged as novel therapeutic targets for human diseases. In this review, we provide a comprehensive overview of the molecular mechanisms governing the activation of inflammasome sensors and the implications of their dysregulated activity in health and disease.

## NLRP1 inflammasome

Human NLRP1 (also known as CARD7, DEFCAP, KIAA0296, NAC and NALP1) is the first receptor that was found to assemble an inflammasome complex [31]. Human NLRP1 has a C-terminal CARD, a centrally positioned function-to-find (FIIND), an LRR, NACHT, and an N-terminal PYD [32]. The NACHT consists of Walker A and B motifs that facilitate ATP binding and hydrolysis for NLRP1 activation [33, 34]. A single gene encodes NLRP1 in humans, whereas three paralogs of NLRP1, encoding NLRP1a, NLRP1b and NLRP1c, are found in mice [35, 36]. Mouse NLRP1a and NLRP1b paralogs possess a C-terminal CARD, and via this CARD, they can bypass ASC by directly recruiting caspase-1 [33, 34]. In humans, NLRP1 is expressed in the stomach, intestines, lungs, testis, and skin and is enriched in barrier cell types such as bronchial epithelial cells and keratinocytes [37–39]. In mice, NLRP1 paralogs are expressed in the hippocampus [40] and in macrophages [41], with little to no expression in keratinocytes [39].

**Fig. 1:**
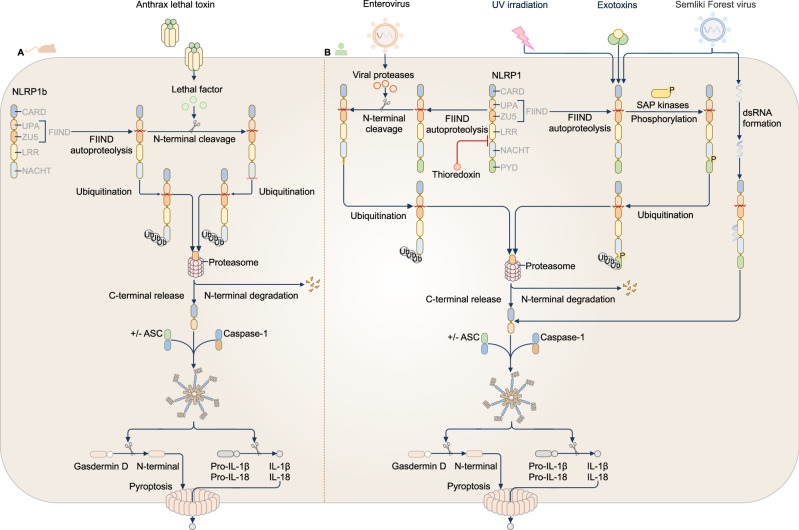
The NLRP1 inflammasome. **A** Murine NLRP1b undergoes autoproteolytic cleavage in the function-to-find domain (FIIND), generating two noncovalently associated fragments that maintain an autoinhibitory state. The activation of NLRP1b is initiated by extracellular stimuli, including anthrax lethal toxin, which cleaves the N-terminal domain of the nucleotide-binding domain (NBD)–leucine-rich-repeat domain (LRR)–FIIND fragment, marking it for ubiquitination and degradation by the proteasome. This process releases the C-terminal fragment (containing a caspase-activation and recruitment domain; CARD), which initiates inflammasome assembly with or without ASC. The assembled inflammasome leads to caspase-1-dependent cleavage of pro-IL-1β and pro-IL-18 and pyroptosis (left). **B** The activation of human NLRP1 also requires a series of proteolytic cleavage events. Human NLRP1 also undergoes autoproteolytic cleavage in the function-to-find domain (FIIND), generating two noncovalently associated fragments that maintain an autoinhibitory state. In addition, oxidized thioredoxin binds to the NACHT-LRR region of NLRP1 and suppresses its activation. Diverse stimuli, including viral protease cleavage, ultraviolet (UV) irradiation, exposure to exotoxins, double-stranded RNA (dsRNA), or stress-activated protein (SAP) kinase–mediated phosphorylation, promote the ubiquitination and degradation of the N-terminal fragment. Proteasomal degradation of the N-terminus releases the active C-terminal UPA-CARD fragment, which forms the NLRP1 inflammasome, triggering caspase-1-dependent cleavage of pro-IL-1β and pro-IL-18 and pyroptosis (right)

An initial 2006 study demonstrated that mouse NLRP1b induces caspase-1-mediated cell death in response to anthrax lethal toxin [35]. Subsequent structural and mechanistic studies clarified the mechanisms of NLRP1 activation (Fig. 1A, B). Prior to activation, NLRP1 undergoes FIIND-mediated autoproteolysis between the ZU5 subdomain and the UPA subdomain, resulting in the generation of an N-terminal fragment and a C-terminal UPA-CARD, which remain noncovalently attached [33, 34, 42]. A key mechanism driving the activation of NLRP1 is the degradation of its N-terminal domain, which releases the C-terminal UPA-CARD that forms the inflammasome [39, 43–45]. This is achieved by stimulation with certain microbial factors, such as toxins, or the chemical inhibitor Val-BoroPro (also known as VbP, Talabostat, or PT100), which inhibits the proteolytic enzymes dipeptidyl peptidase (DPP) 8 and DPP9 [46, 47]. Under steady-state conditions, the FIIND of full-length human, mouse or rat NLRP1 interacts with DPP8 or DPP9 to form an inactive ternary complex that traps the UPA-CARD [48, 49]. Val-BoroPro disrupts this interaction between NLRP1 and DPP8 or DPP9, promoting the accelerated proteasomal degradation of the N-terminal fragment and the release of the UPA-CARD of NLRP1 [49].

Other factors or drivers leading to N-terminal NLRP1 degradation include microbial components, protein folding stress, reductive stress, metabolic stress, and tissue damage [50–53]. In human keratinocytes, for example, NLRP1 can be activated by ribosomal stress in response to ultraviolet light [54–57]. Upon exposure to ultraviolet light, stalled ribosomes cause the activation of stress-activated protein (SAP) kinases, leading to the hyperphosphorylation of serine residues between the PYD and NACHT of NLRP1 [57]. This hyperphosphorylation causes N-terminal NLRP1 degradation through an unknown pathway, leading to the release of UPA-CARDs. Other triggers of SAP kinase activation include exotoxin-A from the bacterium *Pseudomonas aeruginosa*, which activates NLRP1 in human keratinocytes and corneal and airway epithelial cells [58, 59]. Proteases from viruses in the Picornaviridae family induce NLRP1 inflammasome activation by cleaving human NLRP1 between the PYD and NACHT, called the tripwire region [60]. Human NLRP1 is also activated in keratinocytes and bronchial epithelial cells in response to viral dsRNA [61, 62]. NLRP1 may serve as an alternative responder to cellular stress in mammalian cells where other known nucleic acid sensors are absent. For example, NLRP1 is activated in response to the dsDNA poly(dA:dT) in human keratinocytes that lack AIM2 [62]. Similarly, in primary human skin and nasal and corneal epithelial cells that lack NLRP3, NLRP1 can be triggered by the ionophore nigericin, leading to potassium (K^+^) efflux and inflammasome activation [63].

Germline mutations in the gene encoding human NLRP1 are found in patients with autoinflammatory disorders, eye disorders, mucosal inflammation, multiple myeloma, and neurodegenerative diseases [64]. Variations in the gene encoding human DPP9, which can lead to aberrant activation of NLRP1, also contribute to inflammasomopathies. These conditions may present as skin abnormalities, immune response defects, anemia, and increased susceptibility to herpes virus infections [65]. Thus, pharmacological modulation of NLRP1, such as the use of the small-molecule dual NLRP1 and NLRP3 inhibitor ADS032 [66] or the modulation of DPP9 activity, holds substantial promise in targeting NLRP1-mediated immune responses in these disorders. In addition, endogenously oxidized thioredoxin can bind to NACHT and LRR and inhibit human NLRP1 [50, 67], which provides another therapeutic target. Additionally, uncovering the mechanisms of NLRP1 activation, particularly posttranslational modifications that trigger NLRP1 N-terminal degradation, may reveal how NLRP1 responds to different triggers and whether this degradation can be accelerated to enhance the killing of virus-infected cells or inhibited to control sepsis. Furthermore, studying the tissue-specific functions of NLRP1 and its potential coactivation with other immune sensors in response to distinct stimuli could shed light on its broader role in orchestrating immune responses.

A major challenge remains in defining the full spectrum of endogenous and pathogen-derived triggers that induce NLRP1 N-terminal degradation in specific tissues. The lack of NLRP1 expression in murine keratinocytes complicates the use of mouse models in the study of NLRP1-mediated inflammation. Humanized mouse models expressing human NLRP1 in epithelial tissues or organoids and primary human keratinocyte cultures can be employed to more accurately recapitulate NLRP1 activation in vivo. Dissecting the regulation of FIIND autoproteolysis under physiological versus stress conditions is also a key priority. Additional approaches using tissue-specific knockout models and inducible NLRP1 mutants may help address these pressing questions and guide therapeutic targeting of NLRP1 in autoinflammatory and infectious diseases.

## NLRP3 inflammasome

NLRP3 (also known as NALP3, Pypaf1, Cryopyrin and CIAS1) is the best characterized NLR and is expressed in the spleen, intestine, liver, kidneys, lungs and brain of humans and mice, with the highest expression in immune cells [37, 68–70]. Like many other NLR family members, it contains an N-terminal PYD, NACHT and C-terminal LRR. NLRP3 was identified through its association with a group of rare autoinflammatory diseases collectively known as cryopyrin-associated periodic syndrome (CAPS) [71–73]. Earlier studies established that NLRP3 interacts with ASC to form an inflammasome complex [31, 74, 75] following the sensing of PAMPs, DAMPs, and exogenous danger signals [76–79]. Since then, the pathways activated by NLRP3 have been broadly defined as the canonical NLRP3 inflammasome or the noncanonical NLRP3 inflammasome. This section focuses on canonical NLRP3 inflammasome activation, whereas noncanonical NLRP3 inflammasome activation will be further discussed in a separate section.

**Fig. 2:**
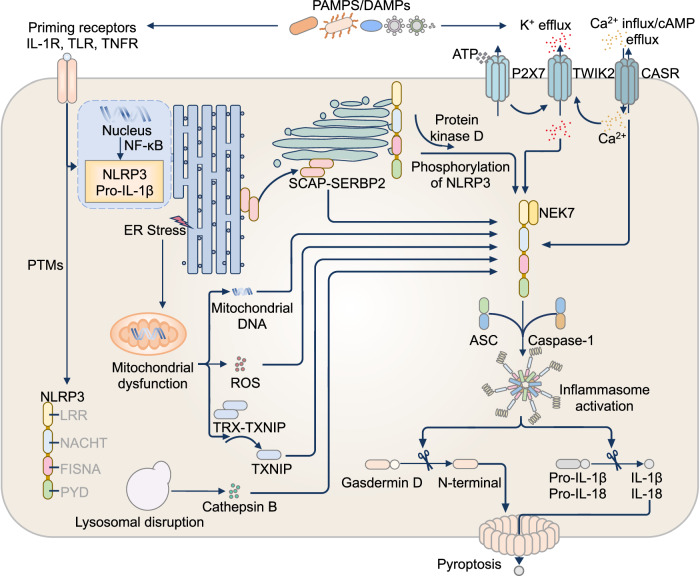
The NLRP3 inflammasome. The NLRP3 inflammasome can be activated via canonical or noncanonical pathways. Canonical NLRP3 activation occurs in a two-step process. The first step, known as priming, is triggered by several classes of receptors in response to pathogen-associated molecular patterns (PAMPs) or damage-associated molecular patterns (DAMPs). The activation of priming receptors stimulates nuclear factor (NF)-κB signaling, resulting in the transcriptional upregulation of NLRP3 and proinflammatory cytokines, including pro-IL-1β. In addition, the priming step promotes posttranslational modifications (PTMs) of NLRP3 to maintain it in a poised state. The second step, or activation, is driven by diverse stimuli, including microbial products, environmental irritants, and cellular stressors, that induce cellular perturbations such as potassium (K^+^) efflux, calcium (Ca^2+^) influx, lysosomal disruption, mitochondrial dysfunction, and endoplasmic reticulum (ER) stress. For example, extracellular ATP binds the P2X7 receptor, affecting TWIK2 channels to mediate K^+^ efflux. Ca^2+^ influx is triggered via the calcium-sensing receptor (CASR), which in turn reduces the level of intracellular cyclic AMP (cAMP), relieving the inhibitory effect of cAMP on NLRP3. Mitochondrial damage results in the release of mitochondrial DNA, reactive oxygen species (ROS), and thioredoxin-interacting protein (TXNIP), all of which contribute to NLRP3 activation. ER stress exacerbates mitochondrial dysfunction to facilitate NLRP3 activation. The translocation of the cholesterol transcription factor and its chaperone, the SCAP–SREBP2 complex, from the ER to the Golgi apparatus under stress promotes NLRP3 activation. Moreover, protein kinase D can phosphorylate Golgi-bound NLRP3, facilitating its release and activation. Disruption of lysosomes leads to the release of cathepsin B, which can also activate NLRP3. Upon activation, NLRP3 binds to NIMA-related kinase (NEK) 7, which stabilizes the active conformation of NLRP3 and facilitates its oligomerization. Activated NLRP3 then recruits the adaptor protein ASC, which in turn binds with caspase-1 to form the functional inflammasome complex

Activation of the canonical NLRP3 inflammasome requires a two-step process involving priming and activation signals (Fig. 2). The priming process is triggered by Toll-like receptors (TLRs) that sense PAMPs and/or DAMPs, leading to the activation of the NF-κB signaling cascade and the transcription of genes encoding NLRP3, pro-IL-1β and other proinflammatory cytokines [80, 81] (Fig. 2). In some cases, cell-surface cytokine receptors such as tumor necrosis factor (TNF) receptors and cytosolic PRRs such as NOD1 and NOD2 can also induce the activation of NF-κB signaling and, therefore, the priming process [80, 81]. Priming also induces posttranslational modifications such as phosphorylation by the kinase PKD [82], palmitoylation by the palmitoyltransferase ZDHHC5 [83], and SUMOylation by the regulatory TRIM protein TRIM28 [84], which collectively promote NLRP3 structure stabilization and inflammasome assembly. NLRP3 activation and inflammasome formation can also be suppressed before or during priming by other inhibitory posttranslational modifications, such as ubiquitination [85–90].

The second step of the activation signal is initiated by a variety of triggers, ranging from PAMPs from bacteria, viruses, parasites, and fungi to endogenous and exogenous signals [91, 92]. These signals alter cellular homeostasis, typically through K^+^ efflux [93–95], lysosomal disruption [96, 97], and mitochondrial dysfunction [98, 99]. In general, NLRP3 activation occurs through K^+^ efflux-dependent or -independent pathways. The K^+^ efflux-dependent pathway is the best characterized pathway and is triggered by the majority of NLRP3 activators [93–95]. In the case of ATP stimulation, for example, ATP binds to the nonselective cation channel P2X purinoceptor 7 (P2X7), which cooperates with the two-pore domain K^+^ channel TWIK2, leading to K^+^ efflux and NLRP3 activation [76, 93, 100, 101] (Fig. 2). In the case of bacterial pore-forming toxins, such as *Bacillus cereus* hemolysin BL and nonhemolytic enterotoxin, these toxins bind to host cell-surface receptors and oligomerize into a membrane pore [102, 103]. These toxin pores disrupt plasma membrane integrity and release K^+^ ions through osmosis, which drive NLRP3 activation [102, 103].

How K^+^ efflux activates NLRP3 is unclear, but it likely requires relieving the autoinhibition of NLRP3. Furthermore, the kinase NEK7 is a crucial component of the NLRP3 inflammasome [104–106], and K^+^ efflux promotes the interaction between NLRP3 and NEK7 [104]. However, K^+^ efflux-independent and reactive oxygen species (ROS)-dependent NLRP3 activation by the chemical compound imiquimod also requires NEK7 [98], indicating that K^+^ efflux must cause other NLRP3-activating cellular changes in addition to promoting the NLRP3‒NEK7 interaction. Structural analysis indicated that NEK7 binds to the LRR of the inactive “cage” conformation of NLRP3 and enables opening of the cage into two halves anchored by an NLRP3 PYD filament [104, 106, 107]. These halves then assemble into an NLRP3 wheel-like oligomer through NLRP3–NLRP3 interactions, with the LRR facing outward and the PYD forming a disk at the center [108]. NLRP3 oligomerization is an ATPase-dependent process in which ATP binds the nucleotide-binding site within NACHT and stabilizes NLRP3 in the active state when hydrolyzed by ATPase elements such as the Walker A and B motifs [109, 110]. The NLRP3 oligomer then acts as a scaffold for ASC recruitment [111–113]. Recruited ASC proteins form a long helical filament where caspase-1 binds to the ASC CARD as the presumed last step of inflammasome formation [111–114]. NEK7 is also phosphorylated at threonine-190 or -191 by the kinase JNK1 following K^+^ efflux, NLRP3 activation, and GSDMD pore formation, providing a positive feedback loop that enhances the binding between NLRP3 and NEK7 [115].

Other forms of cation signaling, such as calcium ion (Ca^2+^) flux, function independently or in concert with K^+^ efflux to trigger NLRP3 inflammasome activation [116–118]. The calcium-sensing receptor (CASR) activates NLRP3 by increasing the level of intracellular Ca^2+^ and decreasing the level of cellular cyclic AMP (cAMP), with cAMP binding to and inhibiting NLRP3 [116, 119]. CASR increases intracellular Ca^2+^ levels by interacting with phospholipase C to increase inositol-1,4,5-trisphosphate production and the efflux of Ca^2+^ ions from the endoplasmic reticulum (ER) [116]. Moreover, CASR decreases cAMP levels by binding to and inhibiting the adenylate cyclase enzyme needed for the conversion of ATP to cAMP [116]. Additionally, an increase in intracellular Ca^2+^ during opening of the P2X7 channel increases K^+^ efflux by activating the Ras-related protein Rab11a, which is necessary for the translocation of TWIK2 to the plasma membrane [120]. Moreover, efflux of chloride ions (Cl^-^) through Cl^-^ channels either promotes IL-1β transcription, the NEK7–NLRP3 interaction, and ASC speck formation and oligomerization [121, 122] or inhibits NLRP3 [123].

In addition to ion flux, cell organelles contribute to NLRP3 inflammasome activation [124]. When dysregulated, mitochondrial components and products activate the NLRP3 inflammasome [125–129]. These include oxidized mitochondrial DNA, which activates NLRP3 and facilitates inflammasome formation [125–127], and the mitochondrial apoptotic effectors BAX and BAK [128]. BAX and BAK trigger NLRP3 inflammasome formation indirectly by increasing caspase-3 and caspase-7 activation and caspase-3- and -7-dependent K^+^ efflux, driving the activation of NLRP3 [128]. Similarly, mitochondrial ROS increase the expression of inflammasome components to mediate inflammasome assembly and additionally dissociate thioredoxin-interacting protein (TXNIP) from thioredoxin to activate NLRP3 [130]. Further work also suggested that certain NLRP3 activators, such as imiquimod, extracellular ATP and the bacterial ionophore nigericin, can inhibit oxidative phosphorylation and, in turn, suppress mitochondrial ATP production and induce damage to the architecture of the mitochondrial cristae [131]. These mitochondrial stressors alone are insufficient to trigger NLRP3 activation but do so in the presence of secondary signals, such as the TLR7/8 agonist resiquimod or Yoda1, an activator of the mechanosensitive ion channel PIEZO1 [131].

The ER contributes to protein synthesis and modifications, such as protein folding, and serves as the assembly line for the active NLRP3 inflammasome. In its resting state, NLRP3 localizes to the *trans*-Golgi network (TGN) as a monomer or in the inactive cage conformation [132]. It is thought that the activation signal prompts a conformational change in NLRP3 and the dispersion of the TGN into vesicles containing NLRP3 [132]. NLRP3 binds to the dispersed TGN through ionic bonding of the polybasic region of NLRP3 and the negatively charged phospholipid phosphatidylinositol 4-phosphate (PtdIns4P) on the dispersed TGN [132]. In response to nigericin, K^+^ efflux does not affect TGN dispersion but is required for NLRP3 recruitment to the remodeled TGN [132]. This model is not universal because, in response to Type A cholesterol-dependent cytolysins, exemplified by perfringolysin O from *Clostridium perfringens*, K^+^ efflux affects neither TGN dispersion nor NLRP3 recruitment to the remodeled TGN [133]. Instead, a small amount of toxins enter the cytoplasm and peel away the PtdIns4P-negative TGN membrane into multiple vesicles, exposing the remodeled PtdIns4P-positive TGN membrane for NLRP3 recruitment [133].

Dispersed TGN vesicles are thought to traffic to the microtubule organizing center, where NEK7 is recruited and activates NLRP3 [108, 132, 134]. The ER further modulates NLRP3 activation through calcium signaling and organelle crosstalk. Inhibition of ER-to-mitochondria Ca²⁺ flux has been shown to impair NLRP3 activation in bone marrow-derived macrophages (BMDMs) [116, 117]. In contrast, ER stress promotes mitochondrial dysfunction, ROS generation, and NLRP3 activation [135]. In addition, perturbed trafficking between organelles can facilitate NLRP3 activation. For example, disruption of ER-endosome membrane sites causes impaired endosome-to-TGN trafficking and accumulation of PtdIns4P in endosomes, which in turn increases NLRP3 recruitment and inflammasome formation [136, 137].

The Golgi apparatus sorts proteins from the ER for transport to the cell membrane and works as a hub for NLRP3 activation signals [113]. A complex formed by the cholesterol transcription factor sterol regulatory element binding protein 2 (SREBP2) and its chaperone SREBP cleavage-activating protein (SCAP) binds to NLRP3 in a ternary complex and escorts it to the Golgi to optimize inflammasome assembly [138]. PKD further phosphorylates NLRP3 on the Golgi, enabling the release of NLRP3 from mitochondria-associated ER membranes and the formation of an inflammasome in the cytoplasm [82]. Lysosomes, which breakdown cellular waste and intracellular pathogens, also enable NLRP3 inflammasome activation. Lysosome-related NLRP3 activation is triggered by the phagocytosis of self or foreign particles, including amyloid-β [139], uric acid crystals [79], cholesterol or deoxyshingolipid crystals [140, 141], silica and aluminum salts [96], or bacterial enzymatic toxins [97]. Furthermore, the stress granule protein DDX3X interacts with NLRP3 to promote inflammasome activation and pyroptosis or relieves NLRP3 to form stress granules to promote cell survival [142].

In human monocytes, NLRP3 activation can occur through alternative pathways. In primary human monocytes, but not in mouse monocytes, LPS-TLR4 engagement triggers the TRIF–RIPK1–FADD–caspase-8 signaling cascade, which drives NLRP3 inflammasome assembly, recruiting ASC and caspase-1 to process IL-1β [143]. Unlike the canonical pathway, this route bypasses K^+^ efflux and induces IL-1β secretion without triggering pyroptotic cell death, representing a nonlytic mode of inflammasome activation [143]. A subsequent study revealed that, in addition to TLR4, other TLRs, including TLR1/2, TLR2/6, and TLR7/8, can also activate NLRP3 via an alternative, nonlytic pathway, in which RIPK1 is dispensable, while K^+^ efflux and pyroptosis are similarly bypassed [144]. Heat-killed gram-negative bacteria also induce NLRP3 activation via a single-step alternative pathway in human monocytes. This pathway is negatively regulated by the short isoform of cellular FLICE-like inhibitory protein (cFLIP_S), which inhibits caspase-8 and reduces IL-1β release [145]. cFLIP_S expression is controlled by TGF-β-activated kinase 1 (TAK1)-dependent NF-κB signaling, and indeed, TAK1 activity is essential for caspase-8 cleavage in response to these bacterial stimuli [145]. Collectively, these studies highlight the breadth of nonlytic alternative mechanisms regulating NLRP3 in human monocytes, underscoring their mechanistic distinction from the canonical two-signal model.

Given the abundance of NLRP3 activators, it is not surprising that the NLRP3 inflammasome has been implicated in many forms of infectious and inflammatory diseases. Indeed, the NLRP3 inflammasome plays an important role in the clearance of bacterial, viral and fungal infections [76, 77, 146–152]. NLRP3 inflammasome activation is stimulated by gram-positive bacteria, such as *Staphylococcus aureus* [76], *Streptococcus* species [153, 154], and *Clostridium* species [97, 155], and gram-negative bacteria, such as *Salmonella* [156–158] and *Yersinia* [159] species, *P. aeruginosa* [160] and *Escherichia coli* [29, 30]. Some bacteria have evolved mechanisms to suppress the NLRP3 inflammasome to increase survival in the host. These bacteria include *Helicobacter pylori*, which reduces NLRP3 activation by mediating mitophagy-mediated degradation of damaged mitochondria via the virulence factor CagA [161], and *Yersinia pestis*, which uses the type III secreted outer effector protein YopK to alter the structure of the type III secretion system and disguise it from NLRP3 sensing [159].

The NLRP3 inflammasome is also involved in antiviral responses to RNA and DNA viruses, namely, influenza A virus (IAV) [151, 162, 163], hepatitis B virus (HBV) [164, 165], Japanese encephalitis virus (JEV) [166], Rift Valley fever [167], encephalomyocarditis virus (EMCV) [168], foot-and-mouth disease virus [169], Mayaro virus (MAYV) [170], varicella-zoster [171], dengue [172] and Zika [173] viruses. NLRP3 is not known to bind directly to viral products. Instead, NLRP3 senses cellular perturbations induced by viral infections, including the activation of the mitochondrial antiviral-signaling protein MAVS, RIPK1-RIPK3-DRP1 signaling, K^+^ efflux, ROS accumulation, lysosome and mitochondrial damage, and the release of oxidized DNA and the lysosomal protease cathepsin B [174–179]. Notably, NLRP3 also has inflammasome-independent functions in antiviral immunity by promoting type I interferon responses. In mice, acetate produced by the gut bacterium *Bifidobacterium pseudolongum* NjM1 activates the host G protein-coupled receptor GPR43, which engages NLRP3 to facilitate MAVS oligomerization on mitochondria [180]. This response triggers TANK-binding kinase 1 (TBK1)-mediated phosphorylation of interferon regulatory factor 3 (IRF3) and subsequent transcription of type I interferons, enhancing antiviral defense against IAV independently of caspase-1 or IL-1β activation [180].

Furthermore, NLRP3 inflammasome signaling mediates immunity to fungal species *Candida albicans* [147, 181], *Aspergillus fumigatus* [182, 183], and *Talaromyces marneffei* [184]. These fungi activate the NLRP3 inflammasome via β-glucan zymosan [185], fungal DNA [182] or the polysaccharide galactosaminogalactan [186]. The production of pro-IL-1β and the release of mature proinflammatory cytokines by the NLRP3 inflammasome during fungal infection can involve crosstalk with other fungal-sensing receptors. For example, Dectin-1 recognizes many fungal species, such that the activation of the NLRP3 inflammasome by certain fungal species is partially mediated by Dectin-1 [187–192]. The NLRP3 inflammasome can also mediate chronic and pathological inflammation during infectious diseases [97, 102, 103, 155, 193–199]. For example, excessive NLRP3 inflammasome signaling is a key contributor to cytokine storms, respiratory distress and organ failure during COVID-19 [193, 194] and exacerbates injury to the lungs and intestinal barrier during bacterial infection [195–197]. Furthermore, it enables persistent human immunodeficiency virus (HIV) infection by contributing to CD4^+^ T-cell death [200].

In humans, gain-of-function mutations in the gene encoding NLRP3 drive overactivation of NLRP3 and the development of CAPS [72, 73]. CAPS can be divided into three subtypes on the basis of severity and onset. The mildest form of CAPS is familial cold autoinflammatory syndrome (FCAS), followed by Muckle–Wells syndrome (MWS), with the most severe form of CAPS being neonatal multisystemic inflammatory syndrome (NOMID) [72–74, 201]. NLRP3 mutations enhance NLRP3 inflammasome activity through various mechanisms, including increased ATP binding, oligomerization of the PYD, and reduced binding affinity with NLRP3-inhibiting cAMP molecules [116, 202]. Mapping of CAPS mutations to the NLRP3 structure revealed that most mutations, including the common R260W, L305P, T348M and A439V mutations, are located within the NACHT [107]. These mutations destabilize the inactive conformation of NLRP3, thereby lowering the activation threshold [107]. Certain mutations hypersensitize NLRP3 to cold exposure and nigericin, and most CAPS-associated variants are responsive to the NLRP3 inhibitor MCC950 (also known as CP-456,773 and CRID3) [202]. Although MCC950 has shown efficacy in preclinical models, its toxicity has limited its clinical utility [203]. Therefore, the development of next-generation safer NLRP3 inhibitors remains a critical therapeutic goal for the treatment of CAPS and other NLRP3-dependent inflammatory diseases.

In addition to infectious and genetic conditions, NLRP3 activation contributes to the development of neurogenerative disorders, Alzheimer’s disease [139, 204], Parkinson’s disease [205], cancer [206–212], atherosclerosis [140], gout [79], inflammatory bowel disease (IBD) [206, 213–215], liver diseases [216–219], obesity [216], rheumatoid arthritis [220], and type 2 diabetes [221]. In the case of IBD, for example, the NLRP3 inflammasome can elicit both protective and damaging effects. NLRP3 inflammasome activation in the gut causes excessive inflammation during IBD; however, defective NLRP3 inflammasome formation results in a loss of gut epithelial integrity, bacterial overgrowth, and increased susceptibility to dextran sodium sulfate (DSS)-induced colitis [206, 213, 214]. A similar duality in NLRP3 function is observed in certain cancers. The NLRP3 inflammasome has antitumor effects in colon cancer but promotes tumor growth in pancreatic and breast cancers and confers resistance to checkpoint inhibition therapy [206–208, 214, 222]. The mechanisms underlying these context-dependent effects remain unclear, highlighting the need for further research into NLRP3 activation and regulatory pathways and their crosstalk with other immune signaling networks.

A key challenge is to define the context-specific mechanisms that render NLRP3 activation protective or pathological across tissues and disease states. It remains unclear how upstream signals, posttranslational modifications, and organelle crosstalk integrate to fine-tune NLRP3 activation in vivo, particularly under chronic or low-grade inflammatory conditions. Experimental strategies to address these questions may include proximity labeling and proteomics to identify novel NLRP3-interacting factors under distinct activation states, to map posttranslational modifications regulating NLRP3, CRISPR-based screens to reveal upstream regulators or inhibitory pathways and single-cell RNA sequencing to define cell-specific transcriptional programmes associated with canonical or alternative activation. Collectively, these approaches may help unravel the molecular, cellular, and contextual determinants of NLRP3 signaling and could guide the development of safer, context-specific therapeutic interventions for infections, autoinflammatory diseases and cancer.

## Noncanonical inflammasomes and caspase-4/5/11

**Fig. 3:**
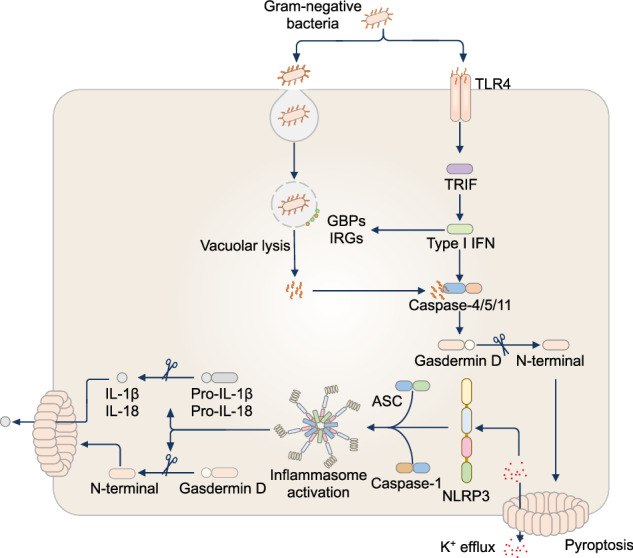
Noncanonical inflammasomes and caspase-4/5/11. In the noncanonical inflammasome pathway, lipopolysaccharide (LPS) from gram-negative bacteria activates the Toll-like receptor 4 (TLR4)–TIR-domain-containing adapter-inducing interferon (IFN)-β (TRIF) signaling pathway, leading to the upregulation of guanylate-binding proteins (GBPs) and immunity-related GTPases (IRGs) via the type I IFN pathway. GBPs and IRGs target outer membrane vesicles or bacterial and vacuolar membranes to facilitate the release of LPS into the cytoplasm. The binding of LPS to murine caspase-11 or human caspase-4/5 leads to the cleavage of gasdermin D, triggering pyroptosis and potassium (K^+^) efflux. This efflux of K^+^ activates the noncanonical NLRP3 inflammasome

The outer layer of gram-negative bacteria comprises LPS, a potent endotoxin and a widely studied PAMP that triggers the immune response. The extracellular sensing of LPS by Toll-like receptor 4 (TLR4) initiates NF-κB signaling, whereas the cytosolic sensing of LPS by caspase-11 in mice and the orthologs caspase-4 and caspase-5 in humans initiate the activation of the noncanonical inflammasome [29, 223, 224] (Fig. 3). Activated caspase-11, caspase-4, or caspase-5 induce direct proteolytic cleavage of GSDMD, liberating the N-terminal domain of GSDMD, which forms plasma membrane pores, leading to pyroptotic cell death [8–12]. These GSDMD pores also mediate the efflux of K^+^ ions from within the cell, which drives intracellular ionic perturbation and the activation of the NLRP3 inflammasome, leading to caspase-1-dependent proteolytic cleavage of IL-1β and IL-18 [225–227]. The requirement for caspase-11, caspase-4, and caspase-5 in the activation of the NLRP3 inflammasome is referred to as the noncanonical inflammasome pathway (Fig. 3).

Murine caspase-11 shares 60% protein identity with human caspase-4 or caspase-5 [224, 228]. However, a notable difference is that caspase-11 cannot directly induce the proteolytic cleavage of IL-1β and IL-18 into their bioactive forms, whereas caspase-4 and caspase-5 can form a protein complex with pro-IL-18, inducing the cleavage of pro-IL-18 into its bioactive form with a similar efficiency as that of caspase-1 [229–231]. In some cases, a p37 form of caspase-4 can be generated that has an impaired ability to process IL-18 [232]. The recognition and processing of substrates by these inflammatory caspases are nuanced because these caspases can cleave IL-1β, generating an inactive 27-kDa IL-1β fragment that deactivates IL-1β signaling [229].

Caspase-11 is composed of 373 amino acids with a molecular weight of 43 kDa and has an N-terminal CARD and a C-terminal caspase catalytic domain [224]. Caspase-11 is activated following direct binding between the N-terminal CARD and LPS [9]. LPS is composed of an O-antigen, a core oligosaccharide, and lipid A [233]. Most lipid A species bind and activate caspase-11, but underacylated lipid IVa and LPS from *Rhodobacter sphaeroides* can bind to caspase-11 but cannot induce caspase-11 activation [9]. Furthermore, pentaacylated and hexaacylated lipid A, but not tetraacylated lipid A, can activate caspase-11 [234]. Upon binding to LPS, caspase-11 proteins form oligomers via CARD-CARD interactions [9, 235], but chemical-induced dimerization of caspase-11 is sufficient for activation [236]. Caspase-11 then cleaves itself at D285 within the linker connecting the large and small enzymatic subunits within the C-terminal caspase catalytic domain, which is required for subsequent proteolytic cleavage of GSDMD [236, 237]. Further studies suggest that GSDMD pores that form on the mitochondrial membrane induce the release of mitochondrial DNA into the cytoplasm, which, along with LPS, facilitates the interaction between NLRP3 and another cytosolic sensor, Nur77, triggering inflammasome activation [238]. Additionally, the LPS-binding protein CD14, independent of TLR4, mediates the cytosolic localization of LPS to enable caspase-11 activation [239].

Caspase-4 comprises 377 amino acids with a molecular weight of 42 kDa [240–242], whereas caspase-5 comprises 434 amino acids with a molecular weight of 48 kDa [242, 243]. Like caspase-11, the two human orthologs also possess an N-terminal CARD and a C-terminal caspase catalytic domain required for cleaving GSDMD [12]. Like caspase-11, caspase-4 and caspase-5 also interact with the lipid A motif of LPS through their respective CARD [9]. However, caspase-4 and caspase-5 seem to have broader detection repertoires than does caspase-11, with these human caspases being able to bind pentaacylated, hexaacylated, and tetraacylated LPS [244]. Furthermore, caspase-11, caspase-4 and caspase-5 can be activated by LPS presented on bacterial outer membrane vesicles (OMVs) [245–247] and by lipooligosaccharide (LOS), which contains a core oligosaccharide and lipid A domain but not the O-antigen chain [233], which is found in bacteria such as *Moraxella catarrhalis* and *Neisseria gonorrheae* [245, 248].

Type I interferon signaling plays a fundamental role in licensing the activation of caspase-11 [249–251]. TLR4 recognition of extracellular LPS induces the expression of procaspase-11, whereby endosomal uptake of LPS initiates the TLR4-TRIF signaling pathway, leading to the activation of the transcription factors IRF3 and IRF7 and the upregulation of type I interferons [150, 249–252]. Secreted type I interferons can act in an autocrine and/or paracrine manner by binding to the type I interferon receptors IFNAR1 and IFNAR2, activating the STAT1-STAT2-IRF9 pathway that drives pro-caspase-11 expression [249–253]. Caspase-4 differs from caspase-11 and caspase-5 in that it is constitutively expressed [253]. In addition to the upregulation of the procaspase-11 protein, the type I interferon pathway increases the expression of guanylate-binding proteins (GBPs) and immunity-related GTPases (IRGs) [254, 255]. Following the phagocytosis of bacteria such as *Citrobacter koseri* [256], *E. coli* [257], *Enterobacter cloacae* [256], *Legionella pneumophila* [258], and *Salmonella enterica* serovar Typhimurium (also known as *S*. Typhimurium) [256], GBPs and/or IRGB10 can permeabilize bacteria-containing vacuoles and/or lyse bacterial cells directly, releasing LPS into the host cell cytoplasm. For example, mouse GBP2 is recruited to vacuoles containing *C. koseri*, *Enterobacter cloacae, L. pneumophila* and *S*. Typhimurium as early as 20 minutes after infection [258], rupturing the vacuolar and bacterial membranes and leading to LPS-induced caspase-11 activation in macrophages [256, 259, 260]. Similarly, mouse GBP1, GBP2, GBP3 and GBP5 are recruited to *M. catarrhalis*, promoting GBP-dependent bacterial lysis and LOS-induced caspase-11 activation in macrophages [245]. Caspase-11 activation, therefore, requires GBPs to liberate LPS or LOS from pathogen-containing vacuoles and pathogens themselves. In human cells, caspase-4 activation by LPS also requires GBPs [261, 262]. In this case, human GBP1 can directly bind to the LPS of *S*. Typhimurium and *Shigella flexneri* [263, 264]. Up to 30,000 human GBP1 molecules are thought to be recruited to the outer membrane of bacteria [265, 266] and facilitate rupture of the bacterial membrane [267, 268]. The initial coating by human GBP1 mediates the recruitment of human GBP2, GBP3 and GBP4 to the bacterial surface, where caspase-4 can subsequently dock to this GBP complex and interact with LPS [263, 264].

The importance of caspase-11 in mediating host defense against gram-negative bacteria has been demonstrated in mouse models of *Acinetobacter baumannii, Burkholderia* species, *E. coli*, and *M. catarrhalis* [29, 245, 269–271]. Caspase-11 reduces bacterial burden and/or lethality following infection with *A. baumannii, Burkholderia thailandensis*, *Burkholderia pseudomallei*, or *M. catarrhalis* [245, 269, 271]. In contrast, systemic activation of caspase-11 in response to LPS leads to sepsis and lethality in mice [10, 29, 30, 223, 224, 234, 238, 272–274]. These opposing outcomes highlight the protective role of caspase-11 during localized infections and its detrimental role during systemic inflammation.

Studies have shown a broader role for caspase-4, caspase-5, and caspase-11 in mitigating infectious diseases that are not driven by LPS or gram-negative bacteria [272, 275]. The gram-positive bacterium *Streptococcus pyogenes* and its lipoteichoic acid (LTA) and extracellular vesicles can activate caspase-4 and caspase-5 in human monocytes via MyD88, RIPK1, and caspase-8 [276]. Both the parasite *Toxoplasma gondii* and the fungal pathogen *Aspergillus fumigatus* lack LPS or LOS, but *Casp11*^–/–^ mice have reduced inflammation and attenuated disease severity in response to infection with *T. gondii* [275], and *Casp11*^–/–^ mice infected with *A. fumigatus* succumb faster than do wild-type mice [272]. These unexpected observations indicate that these inflammatory caspases may sense additional PAMPs or DAMPs. In addition to LPS or LOS, caspase-11 can bind and sense oxidized phospholipids in murine dendritic cells [277], but caspase-11 also appears to be inhibited by oxidized phospholipids in murine macrophages [278]. Furthermore, caspase-11 and caspase-4 can bind to and be inhibited by mitochondrial cardiolipin [279]. Regardless, these observations support a model in which host phospholipids, often released by damaged mammalian cells, can activate or interfere with noncanonical inflammasome functions.

The importance of caspase-11 has been further demonstrated by studies showing that pathogens evolve strategies to evade immune detection by caspase-11 [234, 280–284]. For example, caspase-11 is unable to bind to the tetraacylated LPS of *Francisella* species and *Chlamydia trachomatis* [234, 281]. The virulence factor NleF from enteropathogenic *Citrobacter rodentium* and *E. coli* can bind to and inhibit the catalytic domains of caspase-11 and caspase-4, respectively, resulting in impaired host defenses against these pathogens [282, 283]. Other evasion strategies against caspase-4 have been reported. The virulence factor OspC3 in *S. flexneri* binds to the p19 subunit of the caspase-4 CARD, hindering the heterodimerization between the p19 subunit and the p10 subunit required for caspase-4 activation. This inhibition ultimately prevents pyroptosis and promotes bacterial replication in epithelial cells [284]. Therapeutic blockade of these virulence proteins may enhance inflammasome-mediated defense against immune-evading pathogens.

Caspase-11 also protects against intestinal inflammation and colorectal cancer [285–287]. During acute intestinal inflammation, *Casp11*^–/–^ mice treated with DSS are more susceptible and have impaired IL-18 production and epithelial proliferation [285, 286]. Following treatment with the carcinogen azoxymethane (AOM) in combination with DSS, *Casp11*^–/–^ mice develop more intestinal tumors and have impaired IL-1β secretion and STAT1 activation [287]. The possible antitumor role of caspase-11 may inspire further studies into the role of caspase-4 or caspase-5 in IBD and colorectal cancer in humans. Importantly, differences between mice and humans have hindered the development of therapeutics. In sepsis research, for example, experimental mice are highly resilient to LPS, with the doses of LPS used in most studies being approximately a million times higher than those used in human volunteer studies [288, 289], potentially making findings in mice difficult to translate to human clinical trials. A specific inhibitor of caspase-4 or caspase-5 is not clinically approved for therapeutic use. Many available drugs and therapies can target peripheral proteins such as NLRP3, caspase-1, or GSDMD [290–292]. Nevertheless, novel inhibitors of caspase-4 and/or caspase-5 may be developed by modeling similar mechanisms of action to those of bacterial virulence factors or host phospholipids. Research that elucidates the molecular basis of noncanonical inflammasome activation is expected to guide the development of therapeutics. The full spectrum of microbial and host-derived signals beyond LPS that activate caspase-4, caspase-5, and caspase-11 across different tissues and disease contexts remains poorly defined. While GBPs recruit these caspases to bacterial LPS, it remains unclear whether these caspases also engage other bacterial membrane components or how nonbacterial pathogens, such as fungi, and host-derived signals, such as oxidized phospholipids, trigger noncanonical inflammasome activation.

## NLRP6 inflammasome

NLRP6 (also known as NALP6, PYPAF5, PAN3, and CLR11.4) is highly expressed in the large and small intestine but is also expressed in the lungs, liver, kidneys, and brain of humans and mice [293–296]. NLRP6 was first identified in human cell lines as a PYRIN-containing APAF-1-like protein (PYPAF), called PYPAF5, which can activate caspase-1 and NF-κB [297]. Like most other NLRs, NLRP6 consists of three domains: an N-terminal PYD, a central NACHT, and a C-terminal LRR [26]. An earlier study revealed that *Nlrp6*^−/−^ mice produced similar levels of IL-1β following infection with *S*. Typhimurium, *Listeria monocytogenes*, and *E*. *coli* as wild-type mice did [298]. Furthermore, no difference in caspase-1 activation or IL-1β maturation was observed in *Nlrp6*^−/−^ bone marrow-derived macrophages infected with *S*. Typhimurium or *L. monocytogenes*, implying that NLRP6 is not involved in inflammasome signaling during infection [298]. Instead, NLRP6 was found to inhibit NF-κB and ERK signaling in mouse bone marrow-derived macrophages infected with *L. monocytogenes* and *Streptococcus pneumoniae* [298, 299]. However, subsequent reports revealed NLRP6-dependent IL-1β production in mouse bone marrow-derived macrophages infected with *S. aureus* and *S. pneumoniae* and the induction of NLRP6-dependent necroptosis and pyroptosis in macrophages and neutrophils in the lungs [299, 300]. It is also thought that NLRP6 binds to LTA from *L. monocytogenes* during infection in bone marrow-derived macrophages, triggering inflammasome activation [301]. How NLRP6 can form an inflammasome complex but also inhibit NF-κB and ERK in the same cell during the same infection is not known.

**Fig. 4:**
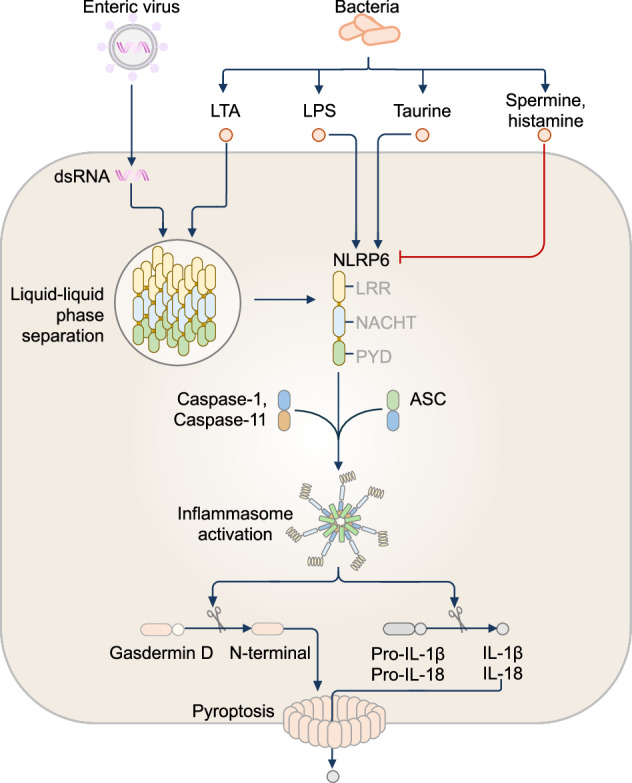
The NLRP6 inflammasome. NLRP6 inflammasome activation is modulated by microbial and metabolic signals. Double-stranded RNA (dsRNA) from enteric viruses or lipoteichoic acid (LTA) from gram-positive bacteria directly interact with NLRP6 to undergo liquid‒liquid phase separation, triggering NLRP6 inflammasome activation. In response to LTA, NLRP6 facilitates the recruitment of both caspase-1 and caspase-11 to the inflammasome complex. The microbial ligand LPS from gram-negative bacteria binds to the NLRP6 monomer, triggering NLRP6 inflammasome activation. The microbial metabolite taurine activates the NLRP6 inflammasome, whereas the metabolites histamine and spermine inhibit NLRP6 inflammasome activation

Several other microbial activators and inhibitors of the NLRP6 inflammasome have since been identified. Microbial metabolites, namely, taurine, histamine and spermine, either activate or inhibit NLRP6 inflammasome assembly [302] (Fig. 4). Taurine activates the NLRP6 inflammasome, leading to increased IL-18 production, whereas histamine and spermine inhibit inflammasome assembly [302]. LPS from gram-negative bacteria binds to NLRP6 monomers and triggers oligomerization with ASC [303], but LPS also initiates inflammasome-independent inhibition of NF-κB and ERK via NLRP6 [304], again highlighting the two facets of NLRP6 signaling. Additionally, double-stranded RNA (dsRNA) and LTA trigger NLRP6 inflammasome formation via liquid‒liquid phase separation, which involves the binding of ASC to NLRP6 condensates [305]. This finding argues for a conceptually different mechanism for NLRP6 inflammasome assembly that departs from the typical oligomer formation that occurs for other inflammasomes.

The different types of PAMPs that can activate or inhibit NLRP6 activity must be considered, especially in the gastrointestinal tract, where NLRP6 expression is highest. During *C. rodentium* infection in mice, a host deubiquitinase called Cyld deubiquitinates NLRP6 through the cleavage of the K63-linked ubiquitination chain on NLRP6 [306]. This event weakens the interaction between NLRP6 and ASC, inhibiting NLRP6 inflammasome formation, which in turn limits IL-18 secretion and severe intestinal inflammation [306]. Earlier reports suggested that the NLRP6 inflammasome maintains gut homeostasis by controlling the composition of the intestinal microbiome, contributing to protection against *C. rodentium* infection in mice [293, 307]. *Nlrp6*^−/−^ mice have an altered gut microbiome, including the expansion of two colitis-inducing pathobionts, the Prevotellaceae family [293] and *Akkermansia muciniphila* [308], and are more susceptible to DSS-induced colitis and tumorigenesis [293]. Furthermore, germ-free *Nlrp6*^−/−^ mice colonized with conventional microbiota developed dysbiosis from 3 weeks of age [302]. However, these microbiome studies lacked littermate controls, and subsequent work has largely argued against an altered gut microbiome in littermate-controlled *Nlrp6*^−/−^ mice [308–310].

NLRP6 has been associated with other inflammatory gastrointestinal diseases, including Crohn’s disease and gastrointestinal symptoms of graft-versus-host disease, and appears to be protective in the development of gastric cancer [311–315]. Epigenetic silencing of the gene encoding NLRP6 enhances cell proliferation and migration during gastric cancer [311], whereas expression of NLRP6 reduces cancer growth via direct ubiquitination and degradation of the molecular chaperone GRP78 in gastric cancer [314]. Finally, reduced NLRP6 inflammasome expression in patients with congenital large intestine conditions and Hirschsprung’s disease, characterized by the absence of nerve cells in parts of the colon, suggests that NLRP6 may be protective in congenital gut diseases [316]. However, exactly how NLRP6 signaling contributes to the health of the gastrointestinal tract during development is unknown. The role of NLRP6 in noninflammasome contexts has also been demonstrated in mouse models of bacterial and viral infections, cancer, and autoinflammatory diseases involving different organs and cell types [304, 317–321]. Overall, NLRP6 remains an enigmatic NLR that plays an important role in homeostasis and disease, with both protective and damaging effects, suggesting many avenues for future studies. How NLRP6 balances its dual functions as an inflammasome sensor and as a negative regulator of NF-κB and ERK signaling remains unclear. It also remains unresolved whether NLRP6 is primarily activated by microbial ligands, host-derived metabolites, or damage signals and how these inputs vary across epithelial, immune, and neuronal cell types. Germ-free or gnotobiotic mouse models with littermate controls, coupled with metabolomics to identify activating or inhibitory metabolites, could resolve the context-specific protective versus pathological functions of NLRP6.

## NLRP7 inflammasome

NLRP7 (also known as NALP7, NOD12 and PYPAF3) is a part of the reproductive NLR family [322] with predominant expression in oocytes [323] and testes [324]. NLRP7 is also expressed in cells and organs of the immune system, including spleen, thymus and bone marrow [324]. NLRP7 is only present in humans, with phylogenetic studies suggesting that NLRP7 evolved from a gene duplication event in primates, resulting in the genes encoding NLRP2 and NLRP7 [325].

**Fig. 5:**
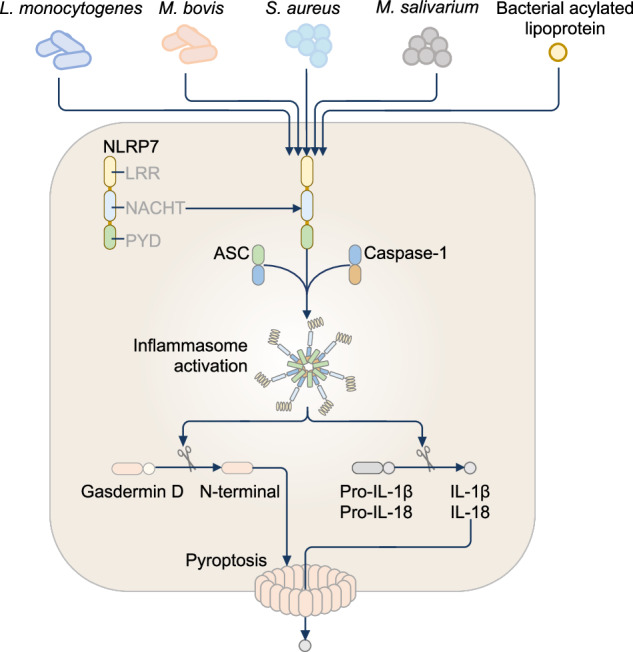
The NLRP7 inflammasome. In human macrophages, the NLRP7 inflammasome is triggered by infections with *Listeria monocytogenes*, *Mycobacterium bovis*, *Staphylococcus aureus*, and *Mycoplasma* species. NLRP7 also detects acylated lipoproteins from *Mycoplasma*, engaging ASC and caspase-1 to assemble an active inflammasome complex

Similar to most NLRP inflammasome-forming proteins, NLRP7 carries an N-terminal PYD, an NACHT, and a C-terminal LRR [26]. Evidence emerged to suggest NLRP7 can form an inflammasome complex. An siRNA screen in human macrophages identified NLRP7 as a sensor of bacterial lipopeptides, including di-acylated and tri-acylated lipopeptides [326]. Whether NLRP7 interacts directly with these lipopeptides or is activated in response to host cellular perturbations triggered by lipopeptides during bacterial infection is unknown. Following exposure to lipopeptides, NLRP7 undergoes a conformational shift, forming a high-molecular-weight inflammasome complex with ASC and pro-caspase-1, leading to the release of IL-1β and IL-18 [326]. Silencing of the gene encoding NLRP7 in human macrophages leads to a reduction in IL-1β and IL-18 release during infection with the bacterium *S. aureus* or *L. monocytogenes*, suggesting that NLRP7 activation mediates an inflammasome response [326] (Fig. 5). NLRP7 inflammasome formation has also been observed in THP-1 macrophages during infection with the bacterium *Mycobacterium bovis* [327], and in amnion epithelial cells stimulated with fibroblast-stimulating lipopeptide from the bacterium *Mycoplasma salivarium* [328]. The oligomerization of NLRP7 during inflammasome formation is mediated by its NACHT, such that the ATP-binding Walker A motif within the NACHT is required for ATP binding, hydrolysis and self-association of NLRP7 [329]. Introducing mutations into this motif, particularly GKT to AAA, impairs inflammasome responses to *S. aureus* infection and acylated lipopeptides [329].

Evidence also suggests that NLRP7 can inhibit inflammasome activation and signaling. For example, NLRP7 competitively interacts with pro-caspase-1 to inhibit NLRP3 inflammasome activation [324]. This observation was confirmed by reduced IL-1β release in THP-1 cells expressing the N-terminal fragment of NLRP7 [324]. The anti-inflammatory functions of NLRP7 also affect other inflammatory proteins and pathways. Indeed, NLRP7 interacts with NF-kB regulatory proteins, such as Fas-associated factor 1, in HEK293 cells to restrain inflammatory responses mediated by NF-kB [324]. However, this anti-inflammatory function of NLRP7 might be dependent on cell types because silencing the gene encoding NLRP7 in THP-1 macrophages, fibroblasts, and primary macrophages did not affect cytokine-induced and LPS-induced NF-kB activation [297, 327, 329, 330]. The pro-inflammatory and anti-inflammatory roles of NLRP7 may also be explained by the presence of multiple functional isoforms differing in the number of LRR generated by alternative splicing events [326, 331–333]. However, further work is needed to verify the functions of these NLRP7 isoforms. The mechanistic switch enabling NLRP7 to trigger anti-inflammatory functions or inflammasome activities also requires further investigation.

Mutations in the gene encoding NLRP7 are typically found within the LRR [334] and affect placental development and early pregnancy [335]. A mutation in the NACHT has also been found in patients with a type of molar pregnancy disorder called hydatidiform mole [336]. Furthermore, genetic variants of NLRP7 have also been found in patients with ulcerative colitis and lung cancer [337–339]. How these disease-associated NLRP7 variants affect inflammasome activation, reproductive cell functions, and/or other pathologies is not clear. It is also unclear whether these mutations drive the dual functions of NLRP7 or whether these functions are governed by isoform diversity or cell type.

## NLRP9 inflammasome

NLRP9 (also known as NALP9, NOD6 and PAN12) is an underexplored member within the NLR family. NLRP9 is predominantly expressed in oocytes, ovaries and testes in humans, mice, and bovines [322, 340–343], suggesting a putative role in reproductive organs. Human NLRP9 is encoded on chromosome 14, which also carries NLRP2, NLRP4, NLRP5, NLRP7, NLRP8, NLRP11, and NLRP13, which are also expressed in reproductive organs [322]. This chromosomal colocation highlights a potential series of tandem duplication events in the evolutionary emergence of this group of NLRs [325]. A single gene encodes NLRP9 in humans, whereas three isoforms encode NLRP9a, NLRP9b, and NLRP9c in mice [325]. Human NLRP9 and the mouse NLRP9 isoforms have a conserved PYD, NACHT, and LRR domain arrangement, similar to most members of the NLR family [26]. Human NLRP9 PYD exists as a monomer in solution, adopting an antiparallel six-helical bundle fold [344–346]. However, the structural details of the remaining NLRP9 domains remain to be resolved.

**Fig. 6:**
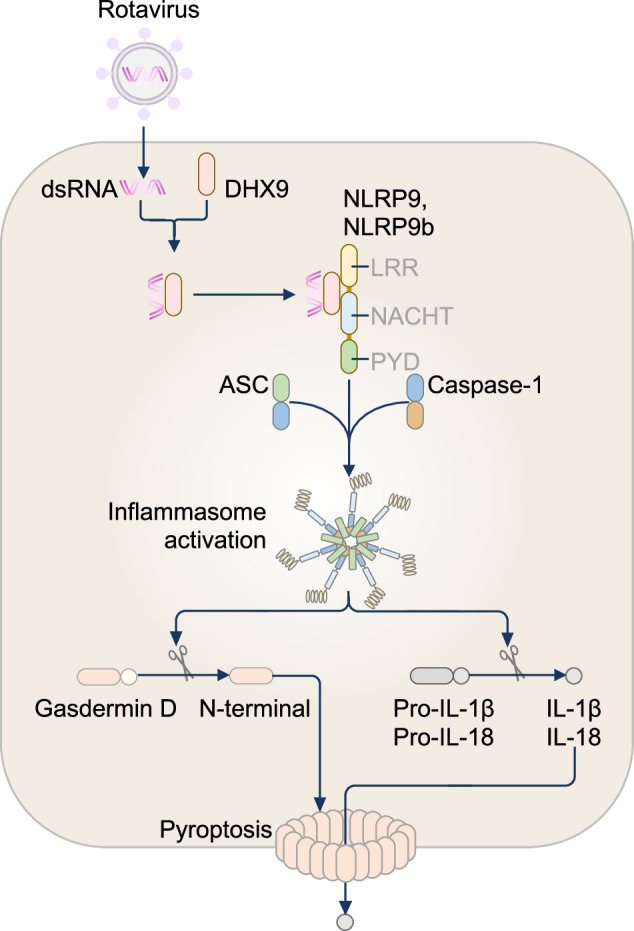
The NLRP9 inflammasome. The mouse NLRP9b inflammasome is activated in intestinal epithelial cells in response to rotavirus infection. Following infection, rotavirus double-stranded RNA (dsRNA) is detected by the RNA sensor DEAH-box helicase (DHX) 9, which directly interacts with NLRP9b to promote NLRP9b inflammasome activation. In human kidney embryonic kidney (HEK) 293T cells, human NLRP9 interacts with dsRNA, DHX9 and ASC

Beyond the reproductive system, human NLRP9 and mouse NLRP9b are also strongly expressed in intestinal epithelial cells and contribute to anti-viral defense [347]. In mice, NLRP9b recognizes viral dsRNA indirectly by acting as an adaptor protein to the RNA-binding helicase DHX-9. DHX-9 binds directly to viral dsRNA and, when complexed with NLRP9b, enables the formation of the NLRP9b inflammasome [347] (Fig. 6). This inflammasome triggers the release of IL-18 and GSDMD-dependent pyroptosis in intestinal epithelial cells and restricts infection by the dsRNA virus rotavirus. Indeed, conditional deletion of NLRP9b in the mouse intestine leads to increased susceptibility to rotavirus [347]. Unlike human NLRP9, mouse NLRP9b does not appear to interact with ASC, suggesting the presence of subtle structural differences between human NLRP9 and mouse NLRP9b [347]. Notably, the PYD of human NLRP9 does not undergo self-oligomerization or nucleate ASC specks [346], despite interacting with ASC in HEK293T cells infected with rotavirus [347]. This may be due to repulsive charge inversions within the PYD interfacing residues of NLRP9, which hinder the interactions between strands of the PYD necessary for self-oligomerization [345, 346]. It is also likely that oligomerization is mediated by another NLRP9 domain or additional binding partners, which also facilitate ASC binding.

Whether NLRP9 can also sense endogenous self-dsRNA and trigger subsequent inflammasome formation is not known. However, self-derived dsRNA species generated through dysregulated epigenetic control of transposable elements, alterations in RNA modifications, genotoxic stress, and mitochondrial stress response [348] could be potential activators of NLRP9. Porcine NLRP9 interacts with the intermediate filament vimentin and induces the production of type I interferons in enterocytes stimulated with synthetic dsRNA poly (I:C) [349]. As vimentin is associated with the accumulation of endogenous dsRNA in fibroblasts within human islet preparations and is also highly expressed in astrocytes within demyelinated multiple sclerosis lesions [350], crosstalk between vimentin, NLRP9 and endogenous dsRNA could contribute to a breach in self-tolerance underpinning Type I diabetes, multiple sclerosis and other autoimmune diseases. Mutations in NLRP9 have already been found in patients with multiple sclerosis [351] but also in patients with Alzheimer’s disease [352] and colon cancer [353]. Investigating how these mutations cause NLRP9 dysfunction and contribute to neurodegeneration, autoimmunity, and cancer could provide deeper insights into the broader role of this inflammasome sensor. Although NLRP9 has an emerging role in antiviral defense, it remains unclear whether the function of NLRP9 is restricted to the intestine or extends to other anatomical sites. Whether NLRP9 senses other RNA viruses or endogenous RNA ligands remains unknown. Immunoprecipitation and crosslinking of RNA of different origins and structural features could verify the ligand-binding repertoire of NLRP9, extending its relevance beyond rotavirus infection and revealing broader roles in diseases.

## NLRP10 inflammasome

NLRP10 (previously known as CLR11.1, PAN5, PYNOD, NALP10, and NOD8) is a newly identified inflammasome sensor [354, 355]. The genes encoding human, mouse, and rat NLRP10 carry only two exons encoding NACHT and PYD, such that NLRP10 lacks the LRR typically found in other NLRs [26]. Human and mouse NLRP10 share 55.5% amino acid sequence identity; human and rat NLRP10 share 55.9%; and mouse and rat NLRP10 share 91.5% amino acid sequence identity [356]. NLRP10 is expressed across most organs in both humans and mice, including the brain, colon, heart, kidney, liver, skeletal muscle, skin, small intestine, and testis [295, 356, 357]. In humans, NLRP10 is more abundant in the colon, liver, muscles, and small intestine, whereas in mice, NLRP10 has the highest expression in the colon, kidney, and testis [295]. Earlier investigations using an overexpression system revealed that NLRP10 inhibited ASC aggregation and caspase-1-dependent cleavage of IL-1β [356, 357], suggesting that NLRP10 has an inhibitory function. Further investigations yielded conflicting results concerning whether NLRP10 is proinflammatory or anti-inflammatory. Some studies suggest a proinflammatory role for NLRP10 in *S. flexneri* infection [358] and skin hypersensitivity [359], whereas other studies revealed an anti-inflammatory role in *Mycobacterium tuberculosis* infection [360], endotoxic shock [357], and fungal and parasitic infections [361, 362]. These context-dependent roles could imply that the function of NLRP10 is highly cell type- and stimulus-specific. NLRP10 was originally thought to initiate the adaptive immune response in mice by triggering dendritic cell migration [363]. However, this purported function of NLRP10 was instead caused by the cytoskeletal protein DOCK8, owing to the presence of a coincidental *Dock8* mutation in the *Nlrp10*^–/–^ mice used in the study [364].

**Fig. 7:**
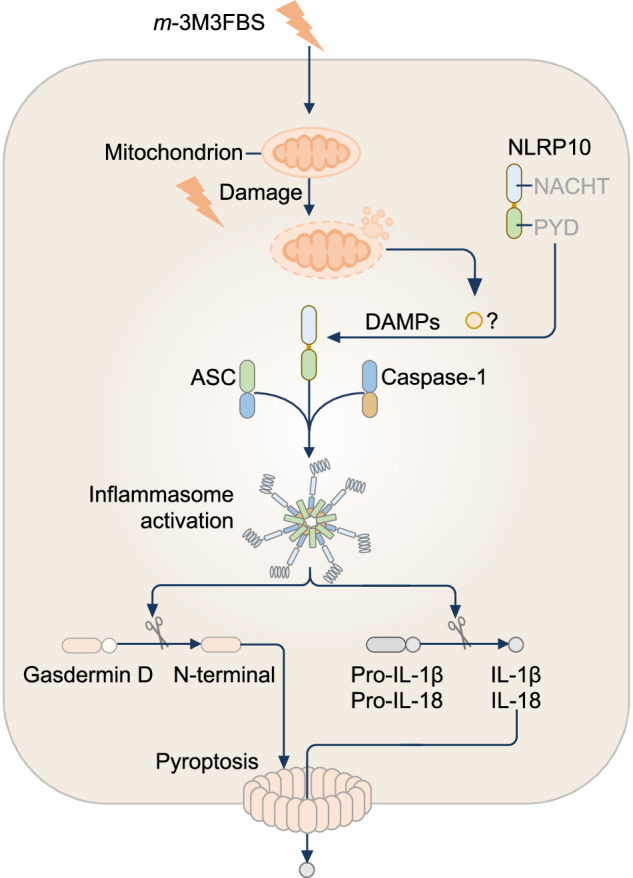
The NLRP10 inflammasome. NLRP10 detects mitochondrial damage caused by the chemical *m*-3M3FBS and assembles an inflammasome complex. Additionally, the proinflammatory agent dextran sodium sulfate (not shown) induces NLRP10 inflammasome formation in mouse colonic epithelial cells through an unknown mechanism

Subsequent studies revealed that NLRP10 has inflammasome-activating effects on primary differentiated human keratinocytes and mouse intestinal epithelial cells [354, 355] (Fig. 7). Upon stimulation with the phospholipase C activator *m*-3M3FBS in differentiated keratinocytes, NLRP10 is recruited to destabilized mitochondria [355]. This mitochondrial localization promotes the assembly of the NLRP10 inflammasome complex, resulting in caspase-1 activation, GSDMD cleavage, and the secretion of IL-1β and IL-18 [355]. Structurally, both NACHT and PYD are necessary for the ability of NLRP10 to function as an inflammasome sensor in response to mitochondrial damage induced by *m*-3M3FBS [354, 355]. Furthermore, the Walker A and B motifs within the NACHT, which mediate ATP binding, are important for NLRP10 inflammasome assembly [354, 355]. Notably, in HEK293 cells expressing NLRP10 carrying an atopic dermatitis–associated mutation, the R243W variant [365], ASC speck formation was abolished following *m*-3M3FBS stimulation, indicating that R243W is a loss-of-function variant that impairs inflammasome assembly [355]. NLRP10 inflammasome activation has also been reported in mouse colonic organoids and in mice treated with DSS [354]. Given that NLRP10 deficiency promotes skin inflammation in humans and exacerbates intestinal inflammation in mice, therapeutic strategies aimed at restoring or enhancing NLRP10 function may represent promising anti-inflammatory approaches.

While AIM2 can directly bind to cytosolic mitochondrial DNA released from damaged mitochondria, NLRP10 does not appear to interact with mitochondrial DNA [354, 355]. In immortalized mouse macrophages depleted of mitochondrial DNA in which the gene encoding AIM2 was replaced with the gene encoding human or mouse NLRP10, ASC speck formation and caspase-1 activation can be triggered following stimulation with *m*-3M3FBS [355]. In addition, the NLRP3-specific inhibitor MCC950 did not reduce IL-1β secretion or ASC speck formation in *m*-3M3FBS-stimulated mouse macrophages [355]. These findings imply that inflammasome activation occurs independently of NLRP3, AIM2 and mitochondrial DNA. The precise ligand(s) of NLRP10 are not yet known, but it is possible that NLRP10 may not bind to any ligands and instead recognizes mitochondrial-specific damage signals or perturbations. Furthermore, the molecular mechanisms guiding the recruitment of NLRP10 to damaged mitochondria and how disease-associated variants such as R243W disrupt the function of NLRP10 are important areas for investigation.

## NLRP12 inflammasome

**Fig. 8:**
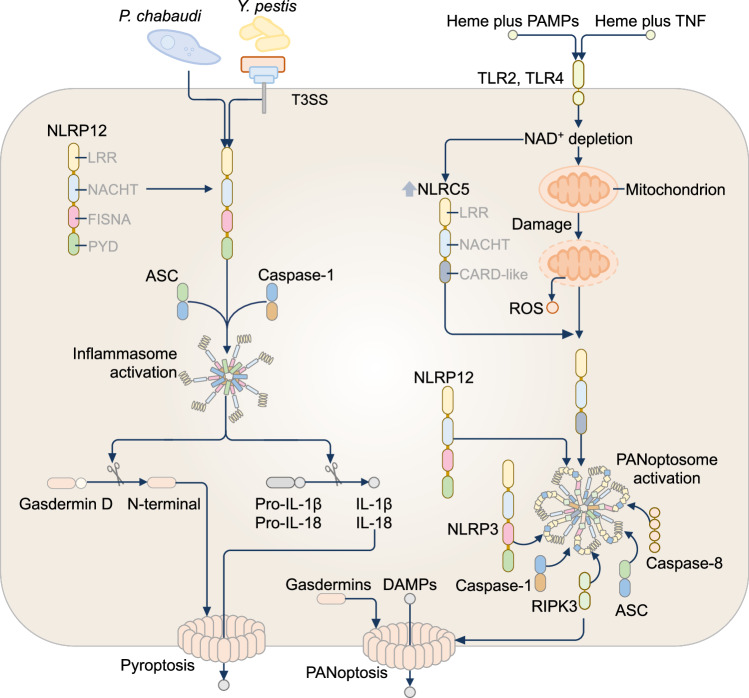
The NLRP12 inflammasome. In mouse macrophages, the NLRP12 inflammasome is activated following infection with *Yersinia pestis* or *Plasmodium chabaudi*. The combination of the heme-containing component of hemoglobin with pathogen-associated molecular patterns (PAMPs) or the cytokine TNF activates Toll-like receptors (TLR2 and TLR4), leading to depletion of cytoplasmic NAD⁺. NAD⁺ loss upregulates the innate immune sensor NLRC5 and induces mitochondrial stress, resulting in reactive oxygen species (ROS) production and the assembly of a PANoptosome complex containing NLRP12, NLRC5, NLRP3, ASC, caspase-1, caspase-8, and RIPK3. This complex drives PANoptosis, a lytic inflammatory cell death pathway mediated by gasdermins, which disrupt the plasma membrane and release damage-associated molecular patterns (DAMPs)

NLRP12 (also known as MONARCH-1, NALP12, PYPAF7 and RNO) was first identified in the human leukemic cell line HL60 [366]. Human NLRP12 is predominantly expressed in myeloid cells, such as macrophages, neutrophils, monocytes, and immature dendritic cells [367]. NLRP12 functions as an inhibitor of inflammation [368–370], an initiator of inflammasome [371, 372], or for scaffolding the PANoptosome [373]. Earlier studies suggest that NLRP12 suppresses canonical and noncanonical NF-kB pathways [368–370], or colocalizes with ASC to inhibit [374] or activate inflammasomes [371, 372]. The first evidence that NLRP12 can assemble a physiological inflammasome complex is in response to the bacterial pathogen *Y. pestis* [372]. The *Y. pestis* Type III Secretion System (T3SS) can activate the NLRP12 inflammasome in mouse macrophages, resulting in the secretion of IL-1β and IL-18 [372]. *Nlrp12*^–/–^ mice exhibit decreased IL-1β and IL-18 secretion, rendering them more susceptible to *Y. pestis* infection compared to wild-type mice [372] (Fig. 8). A further study has shown that NLRP12, in synergy with NLRP3, mediate caspase-1-dependent release of IL-1β and pyroptosis in mouse splenic macrophages and dendritic cells in response to the parasite *Plasmodium chabaudi* [371] (Fig. 8). In contrast to these studies, another study has shown that ectopically expressed human NLRP12 interacts with human NLRP3 in HEK293T cells, leading to the inhibition of the NLRP3 inflammasome [374]. The PYD of NLRP12 can also form a heterotypic interaction with the inhibitory protein of NF-kB signaling, FAF-1 [375, 376], which might provide another mechanism by which NLRP12 inhibits pro-inflammatory responses. The switch in mechanisms between pro-inflammatory functions and anti-inflammatory functions by NLRP12 remains to be resolved.

The precise microbial ligands from *Y. pestis* or *P. chabaudi* that activate NLRP12 are not known. However, a recent study has potentially shed light on the role of NLRP12 in sensing PAMPs and DAMPs. In response to heme in the presence of TNF or activators of TLRs, such as LPS, PAM3CSK4 or R848, the transcription factor IRF1 induces the expression of NLRP12 [373]. NLRP12 is required to drive the assembly of an NLRP12 PANoptosome complex containing NLRP12, NLRP3, ASC, caspase-1, caspase-8, and RIPK3 [373]. This PANoptosome mediates lytic inflammatory cell death and IL-1β and IL-18 secretion [373]. Furthermore, NLRC5 has been identified as another NLR that is part of this protein complex, functioning as a sensor of both NAD⁺ depletion and ROS production induced by LPS and heme [377]. NLRC5 and NLRP12 can directly interact with one another and facilitate the recruitment of other PANoptosome complex components [377] (Fig. 8). Genetic deletion of NLRP12 in mice causes a reduction in acute kidney damage and lethality in hemolytic disease [373], suggesting a pathological role of NLRP12. Addressing whether *Y. pestis* or *P. chabaudi* infection can activate the NLRP12-NLRC5 PANoptosome could reveal a potential mechanism of transition from the inflammasome scaffold to a PANoptosome scaffold. *Y. pestis* encodes a heme-protein acquisition system that allows the bacterium to use heme as a source of iron [378], raising the possibility that heme could be released during *Y. pestis* infection, activating NLRP12. Furthermore, malaria caused by *P. chabaudi* infection results in the release and accumulation of oxidized heme [379] that might be sufficient to drive NLRP12 activation. Given that NLRP12 has been implicated in both inflammasome and PANoptosome signaling, what mechanisms govern the shift from potentially beneficial inflammasome activity to pathological PANoptotic signaling could clarify the context-specific outcomes of NLRP12 activation.

## NAIP-NLRC4 inflammasome

NLRC4 (also known as CARD12, CLAN, CLAN1 and IPAF) was first identified by a search of genes with sequence similarity to caspase-1 [380]. In humans, NLRC4 is expressed in monocytes, monocyte-derived macrophages, neutrophils, peripheral blood mononuclear cells, and intestinal immune, epithelial and stromal cells, whereas in mice it is found in BMDMs, dendritic cells, neutrophils, intestinal epithelial cells, astrocytes, microglia, and B and T lymphocytes [381, 382]. NLRC4 carries an N-terminal CARD, a NACHT, and a C-terminal LRR. The first evidence that NLRC4 forms an endogenous inflammasome complex came from a study showing that NLRC4 induces caspase-1 activation in BMDMs infected with the bacterium *S*. Typhimurium [383]. Subsequent studies found that *S*. Typhimurium strains lacking flagellar components FliC and FljB cannot robustly activate NLRC4 in wild-type BMDMs [384, 385]. Furthermore, transfection of *S*. Typhimurium flagellin into wild-type BMDMs leads to inflammasome activation [384, 385]. *L. pneumophila* flagellin was also subsequently found to induce the activation of the NLRC4 inflammasome [386–389], firmly establishing NLRC4 as a bona fide sensor of bacterial flagellin. Other virulence factors from bacteria with structures and/or functions similar to flagellin can also activate NLRC4. These protein factors include Type III secretion system components PrgJ from *S*. Typhimurium, Mxil from *S. flexneri*, Pscl from *P. aeruginosa*, and EprJ and Escl from *E. coli* [390].

**Fig. 9:**
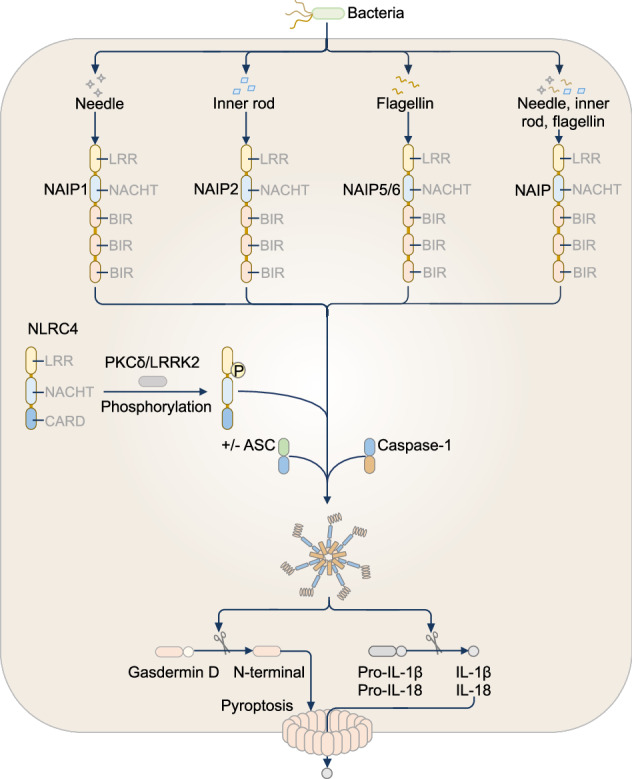
NAIP-NLRC4 inflammasome. The NAIP–NLRC4 inflammasome is activated by bacterial flagellin and components of the type III secretion system (T3SS). In mice, distinct NAIP proteins recognize flagellin, needle, and rod proteins, whereas in humans, a single NAIP protein detects all these ligands. Phosphorylation of NLRC4 by the protein kinase PKCδ and leucine-rich repeat kinase 2 (LRRK2) promotes NAIP–NLRC4 complex formation and the recruitment of caspase-1, with or without ASC, to assemble the inflammasome

As no direct interaction between NLRC4 and bacterial flagellin was reported, it was speculated that additional proteins may act as direct sensors of flagellin that drive NLRC4 activation. During this time, mouse NLR family apoptosis inhibitory protein 5 (NAIP5), one of the seven mouse NAIP paralogs [391], is known to contribute to host resistance to *L. pneumophila* infection and restrict bacterial replication in macrophages by detecting cytosolic flagellin [392, 393]. A conserved C-terminal region of flagellin was identified as the critical domain recognized by mouse NAIP5, which triggers pyroptosis and IL-1β release in macrophages [394]. Notably, flagellin-deficient *L. pneumophila* mutants evade NAIP5- and caspase-1-mediated restriction in mice [395, 396]. These findings established mouse NAIP5 as a cytoplasmic sensor of bacterial flagellin capable of initiating inflammasome-mediated host defense. The molecular connection between NAIPs and NLRC4 became clearer with the discovery that NAIPs are receptors of flagellin and T3SS components that activate the NLRC4 inflammasome [397, 398] (Fig. 9). Mouse NAIP1 and NAIP2 directly bind the T3SS needle and inner-rod proteins, respectively, while mouse NAIP5 and mouse NAIP6 directly bind flagellin [397, 398]. The functions of mouse NAIP3, NAIP4, and NAIP7 remain to be defined. Unlike mice, humans express a single NAIP that appears to be functionally analogous to murine NAIP1 in sensing T3SS needles [399, 400]. Later studies revealed that human NAIP can also sense flagellin and inner-rod proteins.[401, 402] (Fig. 9).

Both human and mouse NAIPs contain a central NACHT with subdomains: helical domain 1 (HD1), helical domain 2 (HD2), and the winged helix domain (WHD) [381]. In addition to NACHT, NAIPs feature a C-terminal LRR and an N-terminal baculoviral IAP repeat (BIR) domain [381]. Cryo-EM structures have elucidated how ligand binding to NAIP drives NAIP–NLRC4 assembly [403–407]. Binding of the *S*. Typhimurium inner rod protein PrgJ to an inactive mouse NAIP2 triggers the formation of a disc structure comprising one mouse NAIP2 monomer and ten mouse NLRC4 monomers [403, 407]. This activated disk structure exposes the CARD of mouse NLRC4, enabling NLRC4 to recruit caspase-1 to form an inflammasome [403, 407]. In the case of the mouse NAIP5–FliC complex, a “trap-and-lock” mechanism sequesters the flagellin D0 domain within the NAIP5 hydrophobic pocket, which is stabilized by the NAIP5 insertion domain located between HD2 and the WHD [406]. An alternative model suggests that a wide-open conformation of mouse NAIP5 has a fully accessible nucleating surface, which recruits NLRC4 [405]. Upon ligand binding, the WHD undergoes a 20° rotation, leading to a steric clash with inactive NLRC4 [405]. This event leads to the transition of NLRC4 from an inactive state to an active state [405]. Subsequent studies demonstrated that, unlike murine NAIPs with discrete ligand-binding surfaces, human NAIP is activated via ligand-induced tightening of its lasso-like motif, a loop formed by the last eight C-terminal residues of human NAIP that thread through the insertion domain and pull it toward the human NLRC4 LRR, stabilizing the complex through additional hydrogen bonds [404]. Specifically, the *Bacillus thailandensis* T3SS needle protein directly interacts with the baculovirus IAP repeat 1 domain of human NAIP, forming a stable needle-NAIP binary complex [404]. This complex induces the conformational change required for human NAIP to recruit and activate NLRC4, thereby assembling the NAIP–NLRC4 inflammasome complex [404]. The findings that ligand-bound NAIPs initiate NLRC4 oligomerization clarify the cooperative relationships between these proteins in inflammasome assembly.

NLRC4 activation is regulated in a context-dependent manner by posttranslational modifications, particularly phosphorylation at S533 [408] (Fig. 9). When mouse BMDMs are infected with *S*. Typhimurium at a multiplicity of infection (MOI) of 1, the kinases PKC-δ and LRRK2 phosphorylate NLRC4 at S533 [408]. Mutation of this residue to alanine (S533A) or pharmacological inhibition of PKC-δ by the nonselective PKC inhibitors staurosporine and K-252a reduces NLRC4 oligomerization and IL-1β release [408], indicating that phosphorylation at this site is required for inflammasome activation. Host-derived retrotransposon RNAs also activate the NLRC4 inflammasome in a manner dependent on S533 phosphorylation [409]. However, other findings complicate this model [410, 411]. NLRC4 is phosphorylated at S533 in the inactive mouse NLRC4 monomer [403], suggesting that phosphorylation may be necessary but not sufficient for NLRC4 activation. Furthermore, when a higher infection dose of *S*. Typhimurium at an MOI of 20 is used, NLRC4 activation occurs regardless of S533 phosphorylation [411], and genetic deletion of PKC-δ does not affect inflammasome activation in response to either *S*. Typhimurium or *S. flexneri* [410].

The interplay between NLRC4 and other inflammasome sensors is essential for mounting effective immune responses against pathogenic and sterile stimuli. Earlier investigations into inflammasome signaling revealed that NLRC4 and NLRP3 have overlapping and cooperative functions in the host defense against *Salmonella* infection [156]. Subsequent mechanistic studies further revealed that NLRC4 and NLRP3 are physically recruited together into a single macromolecular inflammasome complex during *Salmonella* infection [157, 412]. The NLRC4 inflammasome can also recruit other proteases, such as caspase-8, facilitating a coordinated signaling event that enhances the maturation of IL-1β or prevents actin polymerization to limit the intracellular bacterial burden [413, 414]. Importantly, this functional interplay extends to sterile inflammatory processes within the central nervous system. The lipid lysophosphatidylcholine, a DAMP derived from the mammalian plasma membrane and implicated in neurodegenerative diseases and demyelination, activates both NLRC4 and the NLRP3 inflammasome in microglia and astrocytes [415]. Compared with wild-type controls, mice lacking both NLRC4 and NLRP3 exhibit reduced astrogliosis [415], underscoring the contribution of these inflammasome sensors to sterile neuroinflammation. Although the NLRC4–NLRP3 axis is well established, whether NLRC4 interacts with additional inflammasome sensors to drive disease remains an open question.

Aberrant activation of NLRC4 has been implicated in other inflammatory disorders and cancer. For example, gain-of-function mutations of NLRC4 cause or enhance multiple autoinflammatory diseases, including enterocolitis [416], recurrent macrophage activation syndrome [417], and familial cold autoinflammatory syndrome [418]. In allergic asthma, NLRC4 is activated in response to protease allergen-induced release of High mobility group box 1, a protein involved in coordinating cellular stress response [419]. Indeed, upon administration with *Aspergillus* protease ovalbumin, the NLRC4 inflammasome promotes asthma with increased airway hyperresponsiveness and IL-1β secretion in the bronchoalveolar lavage fluid of mice [419]. The functional role of NLRC4 in cancer appears to be highly context dependent. The NLRC4 inflammasome promotes obesity-associated breast cancer progression by promoting angiogenesis and increased expression of vascular endothelial growth factor [420]. In contrast, the NLRC4 inflammasome prevents the high-fat diet-induced growth of colon cancer-derived liver metastasis [421]. In colitis-associated colorectal cancer, the NLRC4 inflammasome has been shown to prevent the development of tumorigenesis [422]. However, other studies using the same mouse model have challenged this conclusion and reported that the NLRP3 inflammasome, instead of NLRC4 inflammasome, is responsible for preventing the development of colorectal tumors [206, 214]. Differences in the findings from these studies may be due to variations in gut microbiome communities among experimental mouse groups, as changes in the gut microbiome profile can substantially influence the progression of intestinal cancer and other diseases [423, 424]. The use of littermate-controlled groups is an optimal approach to standardize the gut microbial communities [425]. Under littermate-controlled conditions, NLRC4 has an inflammasome-independent function by interacting with the ATR-ATRIP DNA damage complex to maintain genomic stability and suppress tumor development [382].

Current approaches to therapeutically target NLRC4 are still in their infancy, but emerging insights from structural and mechanistic studies highlight several promising directions. Given that NLRC4 plays a role in promoting or limiting disease processes, precise modulation of NLRC4 function is essential. Small molecules that enhance or disrupt the interaction between NLRC4 and its interacting proteins represent a promising strategy to target NLRC4-mediated pathways in contexts such as cancers, infectious diseases, and autoinflammatory disorders. Therapeutic modulation of NLRC4 posttranslational modifications, such as phosphorylation, may hold potential and provide context-dependent fine-tuning of inflammasome activity in disease settings. Furthermore, selective targeting of specific NLRC4-NAIP interactions in disease contexts could offer more precise therapeutic strategies than global inhibition of the inflammasome.

## AIM2 inflammasome

AIM2 (also known as Gm1313 and Ifi210) is an inflammasome sensor protein that binds to cytosolic double-stranded DNA (dsDNA) [426–429]. AIM2 consists of a C-terminal hematopoietic interferon-inducible nuclear (HIN) protein with a 200 amino acid repeat (HIN200) domain and an N-terminal PYD [430, 431]. In the resting configuration, AIM2 PYD associates with the HIN200 domain through electrostatic interactions [431]. The AIM2 HIN200 domain binds dsDNA independently of sequence specificity through electrostatic interactions between the positively charged oligonucleotide/oligosaccharide-binding (OB) folds of the HIN200 domain and the negatively charged phosphate groups on the DNA backbone [430]. This sequence-independent recognition mechanism allows AIM2 to recognize dsDNA derived from pathogens and/or pathogen-induced or sterile-induced damage to the host cell. Once the HIN200 domain interacts with dsDNA, the AIM2 PYD can bind other AIM2 PYD and ASC PYD through hydrophobic interactions, triggering inflammasome responses [431]. Evidence also suggests that AIM2 PYD drives both AIM2 filamentation and dsDNA binding but not autoinhibition of the resting AIM2 protein [432].

**Fig. 10:**
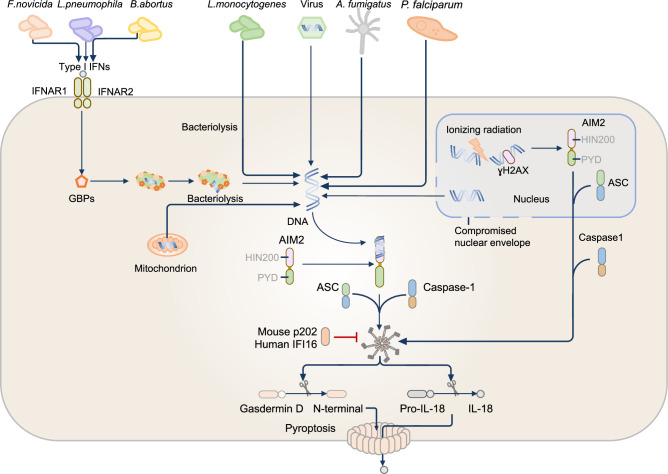
The AIM2 inflammasome. Human and mouse AIM2 inflammasomes are activated in response to microbial or self-DNA. Cytosolic bacteria such as *Francisella novicida*, *Legionella pneumophila* and *Brucella abortus* induce the production of type I IFNs that signal through IFNAR receptors, which drive the expression of guanylate-binding proteins (GBPs) and disrupt bacterial membranes, releasing DNA into the cytosol and facilitating AIM2 activation. In addition, *Listeria monocytogenes* undergoes bacteriolysis, releasing bacterial DNA into the cytoplasm to activate AIM2. DNA viruses such as mouse cytomegalovirus and fungal pathogens such as *Plasmodium berghei* and *Plasmodium falciparum* can activate AIM2 independently of type I IFN signaling. AIM2 also detects radiation-induced damage to host DNA, which is marked by ɣH2AX, in the nucleus. Furthermore, AIM2 recognizes host DNA in the cytoplasm following damage to the nucleus or mitochondria. Following the detection of DNA, AIM2 recruits ASC and caspase-1 to form an active inflammasome complex. AIM2 can be inhibited by several host factors, including p202 in mice and IFN-γ-inducible protein 16 (IFI16) in humans

AIM2 is activated during various scenarios in which dsDNA is released from pathogens. Bacterial infections of *Francisella tularensis* [257, 433–440] and *L. monocytogenes* [441–444], viral infections of herpes simplex virus [445] and pseudorabies virus [446], fungal infection of *A. fumigatus* [182], and protozoal infections of *Plasmodium berghei* [447, 448] and *Plasmodium falciparum* [448] are among the microbial triggers that can activate AIM2 (Fig. 10). In some cases, the activation of AIM2 leads to the recruitment of the cytosolic sensors Pyrin and Z-DNA-binding protein 1 (ZBP1) into the same signaling complex, in addition to ASC and caspase-1, which are typically observed within the inflammasome [445, 449]. AIM2 is expressed at a basal level in the resting state and can be further upregulated by type I interferons [450]. In response to bacterial infection, host interferon-inducible GTPases, including GBPs and the immunity-related GTPase IRGB10, are also globally upregulated in macrophages by type I interferons [255, 451]. Both GBPs and IRGB10 associate with pathogen-containing vacuoles and/or cytoplasmic bacteria, leading to membrane disruption [256, 257, 265, 266, 440]. During *Francisella novicida* infection, mouse GBP1, GBP2, GBP3, and GBP5 are recruited to the surface of the bacterial membrane [440]. A highly hydrophobic stretch within mouse GBP1, GBP1^28–67^, has been identified as an antimicrobial region that directly targets *F. novicida* for bacterial killing [440]. GBPs further recruit IRGB10, which also targets cytoplasmic *F. novicida* [257], where GBPs and IRGB10 collectively compromise bacterial membrane integrity, mediating the release of bacterial dsDNA [257, 437, 438, 440]. Mouse GBP2, GBP3, and GBP5 also promote AIM2 inflammasome activation in macrophages infected with *Brucella abortus* [452]. It is possible that mitochondrial ROS, which are induced during *B. abortus* infection [453], may damage either the bacterial or the mitochondrial membrane that drives the cytosolic release of dsDNA. In other cases, *L. monocytogenes* undergoes autobacteriolysis in the macrophage cytoplasm, which leads to bacterial dsDNA release and AIM2 inflammasome activation [442]. Among bacterial pathogens, the AIM2 inflammasome plays a protective role in mice infected with *F. tularensis*, *B. abortus*, *Mycobacterium tuberculosis* or *S. aureus*, resulting in a reduced bacterial burden and/or disease severity [257, 434, 435, 437, 440, 452–458]. In contrast, the AIM2 inflammasome can also induce excessive inflammation in response to *L. monocytogenes* infection, leading to increased bacterial burden in the liver and reduced survival of mice [459] (Fig. 10).

In addition to its role in bacterial pathogens, AIM2 is also activated during infection with DNA and RNA viruses [460]. For DNA viruses, activation of the AIM2 inflammasome is usually triggered by viral genomic DNA [461, 462]. In the case of HBV, the virus enters the phagolysosome in human monocytes, leading to the release of viral DNA in the host cytoplasm [461]. During human papillomavirus infection, viral dsDNA colocalizes with ASC specks and activates the AIM2 inflammasome in the cytoplasm of human keratinocytes [462]. Among DNA viruses, the AIM2 inflammasome has been shown to play protective roles in mouse models of herpes simplex virus-1 and pseudorabies virus-1 infection, leading to viral clearance [445, 446]. The importance of the AIM2 inflammasome during infection with DNA viruses is further demonstrated by mechanisms in which viruses directly block AIM2, with the tegument protein pUL83 from human cytomegalovirus and VP22 from herpes simplex virus-1 interacting with and inhibiting AIM2 [463, 464].

Notably, AIM2 can also sense infections caused by RNA viruses [465–468]. Although the mechanisms of AIM2 activation induced by RNA viruses are poorly understood, RNA viruses may induce mitochondrial or nuclear damage in mammalian cells, resulting in the release of host dsDNA for AIM2 recognition. Indeed, the IAV proteins M2 and PB1-F2 can damage mitochondrial membranes to trigger cytosolic mitochondrial DNA release [177, 469]. The AIM2 inflammasome, depending on the experimental conditions, plays either protective or detrimental roles during influenza infection. In mice intranasally infected with 40,000 plaque-forming units (PFUs) of IAV PR8, activation of the AIM2 inflammasome leads to increased survival and secretion of IL-1β in the lungs [465]. In contrast, for mice intranasally infected with 40–8000 PFU of IAV PR8, AIM2 promotes lethality [466].

Aberrant DNA release from damaged or dying mammalian cells can also drive sterile inflammation and cancer development [470–474]. Sterile damage induced by the protease inhibitor nelfinavir [475] and the pollutant perfluoroalkyl substances [476] mediates cytosolic dsDNA release from the nucleus and mitochondria, respectively, driving the activation of the AIM2 inflammasome. In the context of cancer, AIM2 either inhibits or promotes tumorigenesis in an inflammasome-dependent or inflammasome-independent manner [471, 472, 477–504]. Most studies describe an inflammasome-independent role of AIM2 in suppressing tumor growth, such as in colorectal cancer [471, 472, 477–480], breast cancer [487–490], cervical carcinoma [484], gastric cancer [485], hepatocellular carcinoma [491, 492], osteosarcoma [486], and renal carcinoma [481–483]. Other studies indicate that AIM2 enhances tumor progression in non-small cell lung cancer [493], lung adenocarcinoma [494, 495] and squamous cell carcinoma [496–498]. Furthermore, AIM2 interacts with the ubiquitin-conjugating enzyme Ube2i to facilitate sumoylation-based suppression of the type I interferon response, limiting kidney inflammation in systemic lupus erythematosus [505]. In the context of an inflammasome-dependent function, AIM2 can be activated by circulating dsDNA in the serum of mice treated with the carcinogen diethylnitrosamine, which promotes inflammasome-mediated inflammation and hepatocellular carcinoma progression [499]. Furthermore, the AIM2 inflammasome enhances non-small cell lung cancer by promoting the entry of cancer cells into the G2/M phase [500]. Nanoparticle-mediated delivery of the gene encoding AIM2 into renal cell carcinoma tissue also promotes caspase-1 activity and IL-1β secretion, leading to inhibition of tumor growth in mice [501]. Owing to both tumor-promoting and tumor-suppressive roles, pharmacological intervention targeting AIM2 activity must be finely controlled for therapeutic purposes.

Therapeutic targeting of AIM2 is a subject of future research because activation of the AIM2 inflammasome is detrimental to many autoimmune and inflammatory diseases [506–518]. The development of AIM2 inhibitors is ongoing. However, no clinically approved drugs are currently available. The synthetic oligodeoxynucleotide A151, via its TTAGGG motif and phosphorothioate backbone, can bind the AIM2 HIN200 domain, block dsDNA binding, and prevent AIM2 inflammasome formation [519]. Additional candidates, such as the seed extract of the *Cornus officinalis* plant [520] and the chemical compounds J114 [521] and 4-sulfonic calixarenes [522], have been reported to inhibit AIM2 inflammasome activation. However, off-target effects have been described for these drugs [519–522], indicating that newer candidates must be developed. Inspirations may be drawn from how AIM2 is inhibited in mammalian cells. AIM2 is constitutively ubiquitinated and undergoes proteasomal degradation [523]. In the presence of dsDNA, the deubiquitinating enzyme USP21 binds to and deubiquitinates AIM2, thereby preventing its degradation and increasing its protein stability [523]. Inhibiting USP21 may be used to degrade AIM2. Furthermore, AIM2 can be blocked by other interferon-inducible HIN-containing proteins, such as mouse p202 and human IFI16-β [524, 525]. Mouse p202 contains a HIN2 domain that interacts with and blocks the AIM2 HIN200 domain [524]. Human IFI16-β is structurally similar and functionally equivalent to mouse p202, which also exerts an inhibitory effect on AIM2 [525]. Although AIM2 is a well-characterized cytosolic dsDNA sensor, the mechanisms controlling its stability, such as ubiquitination and deubiquitination by USP21 or other regulators, including p202 or IFI16-β, require further investigation.

## Pyrin inflammasome

Pyrin (also known as marenostrin, MEFV, MEF and TRIM20) is encoded by the *MEFV* gene and is expressed in cells of the innate immune system, including neutrophils, monocytes, dendritic cells, granulocytes, and eosinophils, in humans [526, 527]. Pyrin was named after the Greek word for fever, Pyretós, owing to the association between *MEFV* variants and familial Mediterranean fever (FMF), the most common noninfectious genetic fever in the world [528]. Pyrin is a member of the TRIM family of proteins, although it is considered an incomplete TRIM protein because of the absence of an E3 ubiquitin ligase domain [529, 530]. Instead, human Pyrin has a five-domain structure consisting of an N-terminal PYD, a bZIP transcription factor domain, a B-box domain, a coiled-coil domain, and a C-terminal B30.2 domain (also known as the SPRY domain) [531]. Mouse Pyrin has a similar domain structure but lacks the B30.2 domain [26].

**Fig. 11:**
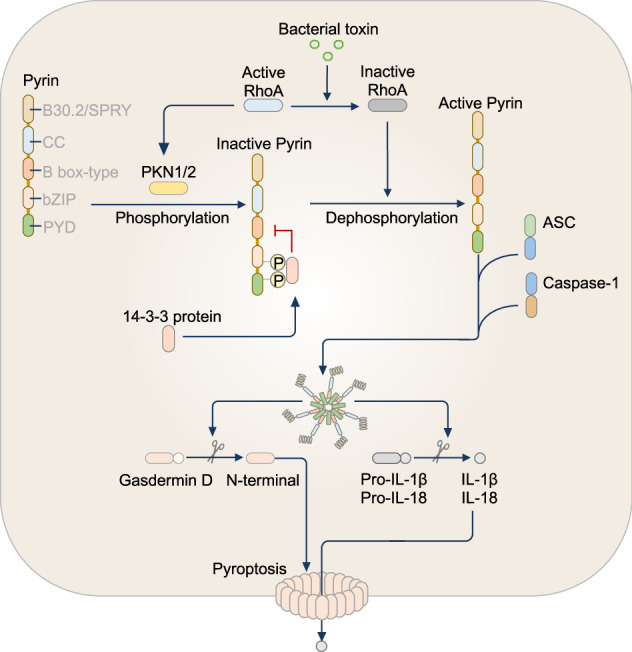
Pyrin inflammasome. Under basal conditions, human and murine pyrin proteins are phosphorylated by the RhoA effector kinase PKN1/2 and bind to 14-3-3 proteins, maintaining them in an inactive state. Bacterial toxins, including *Clostridium difficile* TcdA and TcdB, inhibit RhoA activity and downstream PKN1/2-mediated phosphorylation, resulting in pyrin dephosphorylation and dissociation from 14-3-3 proteins. Activated pyrin then recruits ASC and caspase-1 to assemble a functional inflammasome complex

Whether Pyrin is a genuine inflammasome sensor has been debated for many years. An earlier yeast two-hybrid screen revealed that the PYD of human Pyrin interacts with ASC [532], suggesting that human Pyrin either has a role in inflammasome formation or inflammasome inhibition. In human THP-1 macrophages, siRNA-mediated knockdown of the gene encoding Pyrin led to increased release of IL-1β in response to LPS [533], suggesting that Pyrin may act as a negative regulator of the inflammasome. Similarly, compared with those from wild-type mice, peritoneal macrophages from *Mefv*^–/–^ mice presented increased NLRP3-dependent IL-1β release [534], further supporting a role for Pyrin as a negative regulator of the inflammasome. However, peritoneal macrophages from knock-in mice expressing mouse Pyrin carrying a human B30.2 domain with FMF-associated mutations underwent constitutive caspase-1 activation and ASC dependent, NLRP3-independent IL-1β release [535]. These findings complicated interpretations of Pyrin as a putative negative regulator of inflammasomes or as a bona fide inflammasome sensor. Further clarification of the role of Pyrin in inflammasome biology revealed that Pyrin triggers ASC-dependent caspase-1 activation and IL-1β release in human mononuclear cells infected with the gram-negative bacterium *Burkholderia cenocepacia* [536]. This initial study establishes Pyrin as a putative inflammasome sensor of an infectious trigger. How Pyrin senses *B. cenocepacia* infection in mammalian cells was unknown until a subsequent study linking Pyrin and Rho-GTPase modifications [537] (Fig. 11).

Rho-GTPases drive cytoskeletal remodeling, including actin and microtubule dynamics [538], by switching between an active GTP-bound state and an inactive GDP-bound state [539]. Several bacterial pathogens, including *B. cenocepacia* and *Clostridium difficile*, disrupt actin cytoskeleton dynamics either during entry into or during intracellular replication within mammalian cells [536, 537, 539]. This process is mediated by certain bacterial toxins and effector proteins, including TecA from *B. cenocepacia*, TcdA and TcdB of *C. difficile*, Vops from *Vibrio parahaemolyticus*, lbpA from *H. somni*, and ADP ribosylating C3 toxin from *Clostridium botulinum*. These toxins can modify the switch-1 region of Rho subfamily proteins and activate pyrin [537]. Compared with wild-type BMDMs, *Mefv*^–/–^ BMDMs cannot undergo caspase-1 cleavage and pyroptosis following stimulation with TecA, TcdB, Vops, lbpA or C3 toxin [537]. Furthermore, TcdB, which is defective in glucosyl transferase activity, does not activate pyrin because the glycosylation event at the switch-1 region of RhoA necessitates the inactivation of RhoA [537]. These findings indicate that Pyrin prevents disruptions in RhoA functions triggered by certain bacterial toxins [537]. A recent study identified a novel endogenous regulator of Rho GTPases in the context of bacterial infection. The cell division cycle 42–165 aa (CDC42–165aa), a protein encoded by the circular RNA circCDC42, was found to promote pyrin inflammasome activation and pyroptosis in macrophages during *Klebsiella pneumoniae* infection by inhibiting the activity of the Rho GTPase CDC42 [540]. This discovery further reinforces the role of Rho family GTPase perturbation as a unifying upstream signal for Pyrin-mediated innate immune responses.

Subsequent studies further clarified the mechanisms by which Pyrin detects RhoA inactivation (Fig. 11). Under homeostatic conditions, the Rho-dependent kinases PKN1 and PKN2 (herein PKN1/2) mediate Pyrin phosphorylation at Ser208/242 in humans and at Ser205/241 in mice [541, 542]. Phosphorylated Pyrin then binds to 14-3-3 signaling proteins, maintaining Pyrin in an inactive state [541, 543]. A mutation at the S242R phosphorylation site of human Pyrin attenuates the binding between Pyrin and 14-3-3, resulting in increased ASC speck formation in HEK 293T cells and increased IL-1β release in THP-1 cells [544]. Furthermore, bacterial toxins, such as TcdB, covalently modify RhoA by glucosylation, locking RhoA in its inactive GDP-bound state. Since PKN1 and PKN2 require active GTP-bound RhoA for their catalytic activity, inactivation of RhoA abolishes PKN1/2 function, leading to Pyrin dephosphorylation [541, 542]. This event disrupts the binding between Pyrin and 14-3-3 proteins, possibly leading to a conformational change in Pyrin. These findings highlight the critical role of Pyrin phosphorylation in maintaining Pyrin in an inhibitory state. Importantly, *Yersinia* species have evolved mechanisms to avoid Pyrin activation, such that the *Yersinia* effector protein YopM promotes PKN1/2-mediated Pyrin phosphorylation and prevents Pyrin inflammasome activation [545, 546]. The importance of this regulatory mechanism is underscored by findings that ancient FMF-associated Pyrin mutations may have conferred resistance to *Y. pestis* during historic plague pandemics by enabling heightened inflammasome activation [547].

The assembly of the Pyrin inflammasome is thought to occur at the microtubule organizing center and requires dynein adaptor histone acetyltransferase 6 (HDAC6) and the aggresome processing pathway, which is a cellular pathway that disposes of misfolded proteins [548]. In immortalized BMDMs, HDAC6 contributes to caspase-1 activation, IL-1β secretion, and cell death following TcdB stimulation [548]. However, in the U937 human monocytic leukemia cell line overexpressing Pyrin, HDAC6 is not required for IL-18 secretion and cell death in response to the Pyrin activator and bile acid derivative BAA473 [549], suggesting that the role of HDAC6 in Pyrin activation may be cell-type- and trigger specific. Notably, perturbations in cytoskeletal dynamics can also drive Pyrin-dependent inflammation. For example, a mutation in the actin depolymerization cofactor WDR1 results in systemic autoinflammation in mice [543]. Deletion of the gene encoding Pyrin in *Wdr1* mutant mice leads to decreased serum IL-18 levels compared with those in *Wdr1* mutant mice, which carry a functional Pyrin [543]. A further study suggested that Pyrin activation may be facilitated by the lysosomal regulator complex component LAMTOR1 positioned on the membrane of late endosomes or lysosomes [550]. Compared with wild-type BMDMs, mouse BMDMs lacking LAMTOR1 have an impaired ability to secrete IL-1β following stimulation with TcdB [550]. It is possible that cell types, organelles, and Pyrin triggers converge to drive inflammasome activation and pathological inflammation, underscoring the potentially adaptable mechanisms to different physiological threats. These mechanisms may also explain why Pyrin appears to respond to AOM-DSS in the large intestine of mice, leading to the release of IL-18, maintenance of intestinal barrier integrity, and protection against colitis-associated colorectal cancer [551].

Mutations in the gene encoding Pyrin cause several autoinflammatory disorders. FMF is the most common clinical manifestation arising from mutations in Pyrin, with most of these mutations occurring in the B30.2 domain [544, 552–554]. Blood mononuclear cells from patients with FMF carrying mutant Pyrin show impaired binding between mutant Pyrin and 14-3-3 and between mutant Pyrin and PKN1/2 [542]. However, the structural details of how mutations in the B30.2 domain abrogate the Pyrin–14-3-3 interaction and prevent Pyrin phosphorylation are not known. It is plausible that B30.2 mutations may induce conformational changes that either reduce accessibility to key phosphorylation sites or disrupt autoinhibition, resulting in Pyrin self-oligomerization and inflammasome activation. Recent work also suggests that mutations in the central helical scaffold consisting of coiled-coil alpha helices, in addition to the B30.2 domain, can differentially modulate inflammasome activity, revealing more nuanced Pyrin regulation than previously appreciated [555]. In addition to FMF, Pyrin mutations have been found in patients with Pyrin-associated autoinflammation with neutrophilic dermatosis (PAAND), Pyrin-associated dominant disease (PADD), pyrogenic arthritis, pyoderma gangrenosum and acne (PAPA), mevalonate kinase deficiency (MKD), and autoinflammatory periodic fever, immunodeficiency and thrombocytopenia (PFIT) [542, 556–558]. In these disorders, Pyrin mutations disrupt the interaction between Pyrin and its inhibitory regulators, destabilize the autoinhibitory conformation of Pyrin, or interfere with Rho-GTPase signaling. The net result is aberrant Pyrin activation and excessive IL-1β production, with IL-1β serving as an inflammatory mediator [542, 556–558].

The molecular and genetic basis of Pyrin activation has led to the development of therapeutic strategies, particularly in FMF. Patients with FMF are treated primarily with the anti-inflammatory alkaloid colchicine, which prevents microtubule polymerization, disrupts inflammasome activation, and suppresses IL-1β release [559]. However, in FMF patients who are resistant to colchicine, IL-1-blocking therapies, such as the recombinant IL-1 receptor antagonist anakinra or the anti–IL-1β monoclonal antibody canakinumab, have proven effective at suppressing Pyrin inflammasome–driven inflammation and achieving complete remission of febrile attacks [560, 561]. Future strategies may include the development of small molecules that stabilize Pyrin autoinhibition, for example, by enhancing PKN1/2-mediated phosphorylation of Pyrin or strengthening the binding between Pyrin and 14-3-3. Since Pyrin can detect perturbations in Rho-GTPases [537], further investigations into whether Pyrin can detect Rho-GTPase alterations in cancers and neurological conditions, where Rho-GTPase dysregulation is frequently observed [562–564], could enhance our understanding of Pyrin and guide the development of targeted interventions.

## IFI16 inflammasome

Interferon gamma-inducible protein 16 (also known as IFI16 or IFNGIP1) is a member of the ALR or PYHIN protein family and plays a role in DNA sensing, inflammasome activation, and interferon signaling [565]. IFI16 is found in humans, and the closest ortholog of IFI16, called p204, is found in mice [566]. Multiple cell types express IFI16, including peripheral blood monocytes, T cells, CD34^+^ myeloid precursors, and epithelial cells [567]. Both IFI16 and p204 carry an N-terminal PYD and two C-terminal HIN-200 domains, HINa and HINb, which recognize DNA through oligonucleotide-binding folds [568–570]. IFI16 can bind to DNA fragments of at least 60 bp but optimally binds 150 bp fragments [430, 570]. The HINb domain of IFI16 has a positively charged concave surface that facilitates binding to both strands of dsDNA at the dsDNA backbone, whereas HINa only interacts with one strand at a time, allowing it to bind both ssDNA and dsDNA [569, 571, 572]. In contrast, both HIN domains of mouse p204 bind to DNA via a crosslinker connecting two oligonucleotide binding folds [573].

**Fig. 12:**
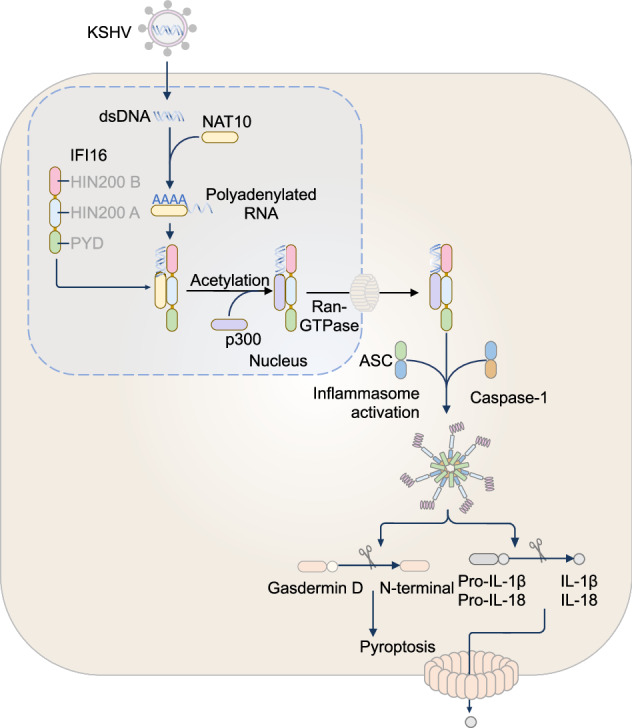
The IFI16 inflammasome. Kaposi’s sarcoma-associated herpesvirus (KSHV) DNA is sensed by interferon gamma-inducible protein 16 in the nucleus. The polyadenylated nuclear RNA of KSHV binds to N-acetyltransferase (NAT) 10, increasing acetylation and increasing the expression of IFI16. Additional acetylation by histone acetyltransferase p300 enables translocation to the cytoplasm via the Ras-related nuclear protein guanosine triphosphate (Ran GTPase), where IFI16 binds viral DNA and assembles an inflammasome

IFI16 is localized in the nucleus, nucleolus, nucleoplasm, and cytoplasm [429, 574–582]. These subcellular localizations determine the role of IFI16 in recognizing cytosolic and nuclear DNA, inducing IFN-β production or inflammasome formation [429, 574–582]. In the cytoplasm, IFI16 senses Vaccinia viral DNA and interacts with STING to increase IFN-β production [570]. In the nucleus, IFI16 can sense HSV-1 DNA to induce the production of type I interferons [583, 584]. In HEK293T cells, mutations in residues within the IFI16 HINa domain involved in DNA binding increase IFN-β release, whereas deletion of HINb attenuates IFN-β expression, suggesting that the complementary domains cross-control IFI16-mediated production of type I interferons [569]. In addition to driving the production of type I interferons, IFI16 can form an inflammasome in the nucleus in response to viral DNA during Kaposi sarcoma herpes virus (KSHV) infection [583]. KSHV encodes a long noncoding RNA known as polyadenylated nuclear RNA, which undergoes acetylation by interacting with N-acetyltransferase (NAT10) [584]. This interaction promotes IFI16 mRNA acetylation via NAT10, increasing IFI16 translation and enhancing DNA sensing [584]. Following recognition of KSHV DNA, IFI16 is also acetylated by the histone acetyl transferase p300 and translocates from the nucleus to the cytoplasm. This translocation is mediated by Ran-GTPase, a GTP-binding protein that controls the movement of other proteins across the nuclear envelope [583]. In the cytoplasm, the PYD of IFI16 interacts with ASC to form an inflammasome [583, 585] (Fig. 12). However, the precise molecular mechanisms governing IFI16 activation, such as acetylation, are unclear. Additionally, how IFI16 activity is restricted to viral DNA and not host DNA within the nucleus requires further exploration. Investigating the potential role of mouse p204 in the formation of an inflammasome may also provide insights into the physiological functions of this pathway. Site-directed mutagenesis of IFI16 acetylation sites, chromatin immunoprecipitation (ChIP) with viral versus host DNA, and comparative studies using murine p204 knockout or human IFI16 knock-in models may clarify the precise mechanisms by which IFI16 senses nucleic acids and drives antiviral immunity.

## CARD8 inflammasome

Caspase recruitment domain-containing protein 8 (CARD8, DACAR, KIAA0955 or NDPP1) is a human inflammasome sensor protein that shares structural homology with human NLRP1 [586] and is expressed predominantly in T cells [587, 588]. No homologs of CARD8 are found in mice [587, 588]. CARD8 consists of a CARD and an FIIND comprising two subdomains called ZU5 and UPA [586]. The structural similarity between CARD8 and NLRP1 initially suggested that CARD8 may function as an inflammasome sensor.

**Fig. 13:**
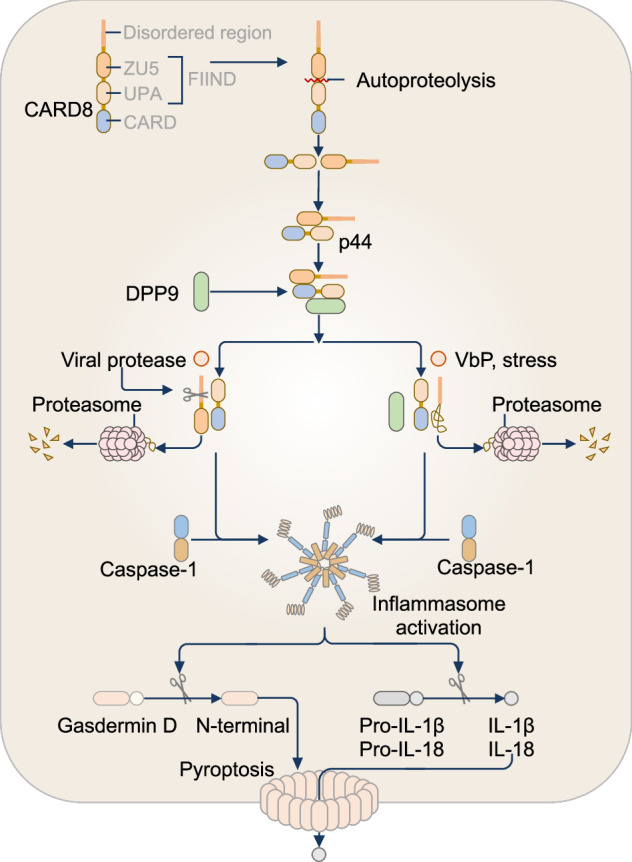
CARD8 inflammasome. Under resting conditions, CARD8 undergoes autoproteolysis and remains inactive through binding to dipeptidyl peptidase (DPP) 8 or 9. Viral proteases from human immunodeficiency virus (HIV) type 1 and Coxsackie virus cleave the disordered N-terminal region of CARD8, releasing the C-terminal fragment, which recruits caspase-1 to assemble the CARD8 inflammasome. The chemical Val-BoroPro or cellular stress disrupts binding to DPP8/9, exposing the N-terminal fragment for proteasomal degradation and liberating the C-terminal fragment to initiate inflammasome assembly

Like NLRP1, CARD8 can be activated by the chemical inhibitor Val-BoroPro, which inhibits the proteolytic enzymes DPP8 and DPP9 [589]. Under homeostatic conditions, CARD8 undergoes autoproteolysis, releasing two fragments: an N-terminal fragment consisting of the FIIND ZU5 subdomain and a 160-amino acid disordered region and a C-terminal fragment consisting of the FIIND UPA subdomain and CARD [590]. These fragments remain noncovalently associated and together form a p44 subunit comprising ZU5, UPA and CARD, which bind the proteolytic enzyme DPP9 [589, 591]. This interaction with DPP9 sequesters the UPA region necessary for CARD8 oligomerization, keeping CARD8 inactive [589, 591]. However, upon Val-boroPro-induced stress, the disordered N-terminal region and ZU5 subdomain are targeted for degradation by the 20S proteasome through an unknown mechanism [591]. This degradation releases the C-terminal UPA–CARD fragment, which can no longer be restrained by DPP9. In the absence of DPP9-mediated restriction, the UPA-CARD region is set free to facilitate CARD8 oligomerization and inflammasome formation [591] (Fig. 13).

The CARD8 inflammasome is activated in response to a range of viral infections and contributes to the pathogenesis of disease development [592–595]. For example, CARD8 activation and subsequent pyroptosis in CD4^+^ T cells infected with HIV-1 drive the depletion of the CD4^+^ T-cell population. The binding between HIV-1 proteases and CARD8 during viral entry [596] or reactivation from latency [595] in CD4^+^ T cells causes proteolysis of the CARD8 N-terminus, leading to the production of an unstable Neo-N-terminus, which is targeted for proteasomal degradation [595]. The subsequent release of a bioactive UPA-CARD fragment results in the formation of an inflammasome complex with caspase-1, leading to the pyroptosis of CD4^+^ T cells and low CD4^+^ T-cell counts [595]. Similarly, the 3 CL^pro^ protease from coronaviruses, including SARS-CoV-2 [594], and the 3C protease from the endocarditis-causing virus Coxsackie virus B3 [593] activate CARD8 by cleaving the CARD8 N-terminal region. This sensing of viral protease activity may be mediated by specific amino acid residues found in human CARD8 but not in other nonhuman primates [594]. For example, CARD8 proteins from nonhuman primates are incapable of sensing HIV-1 protease activity because of the absence of a human-specific F59-F60 motif located within the N-terminal region of FIIND [596], suggesting that polymorphisms of CARD8 found in different host species may explain their susceptibility or resistance to certain viruses.

Other triggers, including unfolded protein response inducers and reductive stress, have also been shown to activate CARD8 by accelerating the degradation of the N-terminal fragment [51, 52]. Additionally, the inhibition of aminopeptidases such as M24B prolidase and Xaa-Pro aminopeptidase-1 by the small-molecule inhibitor CQ31 has been shown to activate CARD8 [597]. CQ31 treatment results in the accumulation of endogenous proline-containing peptides, including Xaa-Pro dipeptides, which inhibit DPP8/9 enzymatic activity and activate CARD8 [597]. As a selective CARD8 activator, CQ31 may serve as a valuable tool for elucidating CARD8 mechanisms and offers therapeutic potential for modulating CARD8 activity to kill virus-infected cells.

In addition to responses to viral infections, CARD8 signaling has been associated with autoimmune, neurodegenerative and cardiovascular diseases. CARD8 mutations have been found in patients with Alzheimer’s disease [598], IBD [599], and rheumatoid arthritis [600]. Heterozygous variants of CARD8 in combination with genetic variants of NLRP3 have been found to increase the risk of ischemic stroke [601]. Future studies characterizing the dysregulated functions of these disease-associated mutations in CARD8 could further reveal opportunities to therapeutically modulate CARD8 activity.

## MxA inflammasome

**Fig. 14:**
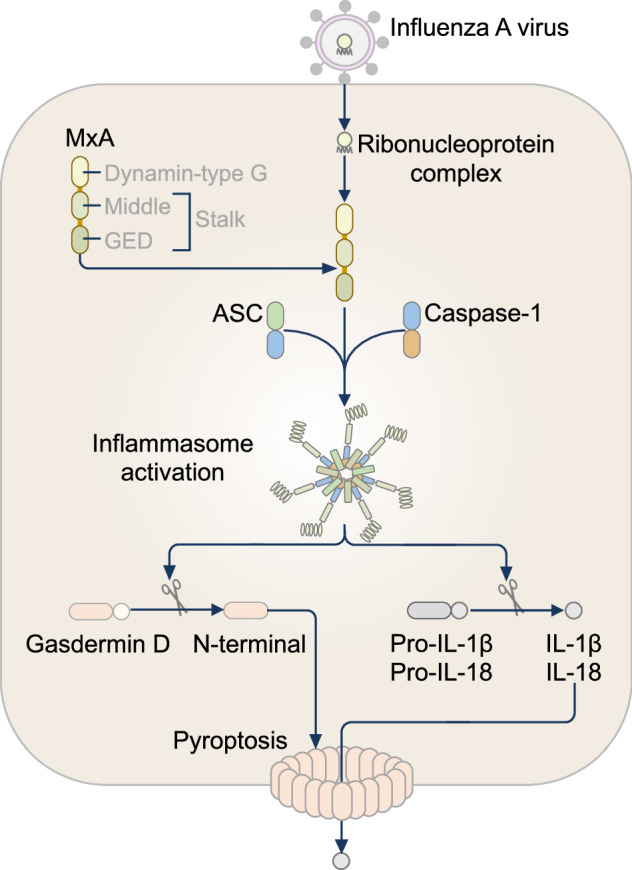
The MxA inflammasome. Myxovirus resistance protein A (MxA) contains a dynamin-type GTPase domain, a middle domain, and a GTPase effector domain that forms a stalk-like structure. During influenza A virus infection, the GTPase domain of MxA binds to the pyrin domain of an apoptosis-associated speck-like protein containing a caspase recruitment domain (ASC), promoting inflammasome activation

The Myxovirus resistance (also known as Mx) proteins are a family of antiviral proteins with pivotal roles in orchestrating innate immune responses against DNA viruses such as hepatitis B virus (HBV) and RNA viruses such as IAV and vesicular stomatitis virus (VSV) [602]. These interferon-inducible proteins are recognized for their ability to impede the viral replication cycle [602–605]. Human Myxovirus resistance protein A (MxA, also known as MX1 and IFI-78K) has been identified as an inflammasome sensor protein that is activated in response to IAV infection [606]. In respiratory epithelial cells, MxA can interact with the ribonucleoprotein complex of IAV, which comprises the viral nucleoprotein and viral polymerase [606]. The nucleoproteins of other viruses, such as vesicular stomatitis virus (VSV) and human parainfluenza virus, are also recognized targets of human MxA [602], but whether MxA senses these nucleoproteins or common structures and/or motifs within nucleoproteins and drives inflammasome activation is not known. The GTPase domain (also known as the dynamin-type G domain) of MxA was found to bind the PYD of ASC, triggering ASC oligomerization [606]. This interaction leads to the formation of an MxA inflammasome complex, leading to the secretion of IL-1β in respiratory epithelial cells infected with IAV [606] (Fig. 14). In cell types that do not express MxA, such as human polymorphonuclear blood monocytes (PBMCs), inflammasome activation in response to IAV infection relies on NLRP3 [606].

C57BL/6J mice do not express functional Mx1 or Mx2 proteins. Compared with conventional C57BL/6J mice, which lack functional Mx1 and Mx2, C57BL/6J mice genetically engineered to carry the entire human Mx locus are more effective at controlling avian IAV infections [606]. Genetic deletion of caspase-1 and caspase-11 in the genetically engineered C57BL/6J mouse strain leads to increased susceptibility to avian IAV infection, highlighting the protective roles of MxA, caspase-1 and caspase-11 in mice. In another mouse model, the C57BL/6J mouse strain, which had been backcrossed to carry functional Mx1, presented increased resistance to human IAV infection [607]. In this mouse genetic background, the role of inflammasomes has been revealed by the genetic deletion of caspase-1 and caspase-11 in *Mx1*^–/–^*Tlr7*^–/–^*Mavs*^–/–^ mice. Compared with inflammasome-competent *Mx1*^–/–^*Tlr7*^–/–^*Mavs*^–/–^ mice, these mice exhibit enhanced protection against human IAV infection and prolonged survival [607], indicating that inflammasomes are detrimental. These findings emphasize that inflammasome functions can either be beneficial or detrimental to the host, depending on the genetic background of the mouse strains used and, in part, whether these mice carry functional Mx proteins.

Mutations in MxA have been found in colon cancer [608], lung cancer [609], and ovarian cancer [610]. In addition, the GTPase domain of MxA is required to inhibit the motility and invasiveness of human prostate carcinoma cells [611], but whether this mechanism is dependent on inflammasome signaling is not known. Therefore, a more in-depth exploration of the molecular mechanisms of MxA function could increase the number of available molecular targets for pharmacological intervention. For example, determining whether MxA recognizes common structural motifs within viral nucleoproteins or specific features of IAV may clarify the breadth of its sensing ability.

## Concluding remarks

The molecular mechanisms governing inflammasome activation has enabled a more in-depth understanding of innate immune responses and their roles in disease pathogenesis. Since the discovery and conceptualization of the inflammasome complex, studies have revealed how different inflammasome sensors are triggered in response to a plethora of PAMPs, DAMPs, and exogenous danger signals. Molecular and structural studies have provided important insights into the activation mechanisms of well-characterized inflammasome sensors. Emerging evidence has also clarified the regulation of inflammasome sensors by unconventional or novel mechanisms. Future research on posttranslational modifications, downstream effects of inflammasome-mediated pyroptosis, and wider implications of inflammasome-mediated immune responses beyond infectious diseases, such as in cancer and autoinflammatory and neurogenerative diseases, are likely areas of active research. Mechanistic and structural studies on the lesser characterized inflammasomes remain an important area for investigation. The inflammasome field has also evolved to a point that fundamental discoveries have been translated to clinical trials of inflammasome inhibitors in humans, particularly in the light of many new and emerging small-molecule NLRP3 inhibitors. These inhibitors will provide hope to patients with aberrant activation of NLRP3 inflammasome, including those with a genetic basis, but inhibitors for most inflammasome sensors are not currently available.

Several critical areas require further research to fully elucidate the role of inflammasomes in health and disease. The context-dependent nature of inflammasome activation, such as the effects of cell types and diseases, remains an active area of inquiry. Moreover, the coordination and potential synergy or antagonism between different inflammasome sensors are poorly understood. For instance, how inflammasome sensor proteins cooperate or compete within a single cell and between different cells to modulate a holistic host response is not known. The revelation of the PANoptosome concept, in which multiple inflammasome components, cytosolic sensors, caspases, and RIPKs aggregate into a single protein complex, represents an example of molecular integration between immune signaling pathways. Further biological insights surrounding these PANoptosomes might fill current knowledge gaps on immune sensing cooperativity and offer new targets for therapeutic development. In conclusion, while substantial efforts have been made to understand the molecular complexities of inflammasome sensors, much remains to be answered. The crosstalk between sensors, the context-dependent nature of activation, molecular mechanisms governing inflammasome signaling, and the identification and clinical development of specific inhibitors are all important focal points as the field moves forward.

## References

[1] Man SM, Karki R, Kanneganti TD. Molecular mechanisms and functions of pyroptosis, inflammatory caspases and inflammasomes in infectious diseases. Immunol Rev. 2017;277:61–75. 28462526 10.1111/imr.12534 PMC5416822

[2] Rathinam VA, Fitzgerald KA. Inflammasome Complexes: Emerging Mechanisms and Effector Functions. Cell. 2016;165:792–800. 27153493 10.1016/j.cell.2016.03.046 PMC5503689

[3] Barnett KC, Li S, Liang K, Ting JP. A 360 degrees view of the inflammasome: Mechanisms of activation, cell death, and diseases. Cell. 2023;186:2288–312. 37236155 10.1016/j.cell.2023.04.025 PMC10228754

[4] Van Opdenbosch N, Lamkanfi M. Caspases in Cell Death, Inflammation, and Disease. Immunity. 2019;50:1352–64. 31216460 10.1016/j.immuni.2019.05.020 PMC6611727

[5] Kesavardhana S, Malireddi RKS, Kanneganti TD. Caspases in Cell Death, Inflammation, and Pyroptosis. Annu Rev Immunol. 2020;38:567–95. 32017655 10.1146/annurev-immunol-073119-095439 PMC7190443

[6] Ding J, Wang K, Liu W, She Y, Sun Q, Shi J, et al. Pore-forming activity and structural autoinhibition of the gasdermin family. Nature. 2016;535:111–6. 27281216 10.1038/nature18590

[7] Liu X, Zhang Z, Ruan J, Pan Y, Magupalli VG, Wu H, et al. Inflammasome-activated gasdermin D causes pyroptosis by forming membrane pores. Nature. 2016;535:153–8. 27383986 10.1038/nature18629 PMC5539988

[8] Shi J, Zhao Y, Wang K, Shi X, Wang Y, Huang H, et al. Cleavage of GSDMD by inflammatory caspases determines pyroptotic cell death. Nature. 2015;526:660–5. 26375003 10.1038/nature15514

[9] Shi J, Zhao Y, Wang Y, Gao W, Ding J, Li P, et al. Inflammatory caspases are innate immune receptors for intracellular LPS. Nature. 2014;514:187–92. 25119034 10.1038/nature13683

[10] Kayagaki N, Stowe IB, Lee BL, O'Rourke K, Anderson K, Warming S, et al. Caspase-11 cleaves gasdermin D for noncanonical inflammasome signaling. Nature. 2015;526:666–71. 26375259 10.1038/nature15541

[11] He WT, Wan H, Hu L, Chen P, Wang X, Huang Z, et al. Gasdermin D is an executor of pyroptosis and required for interleukin-1beta secretion. Cell Res. 2015;25:1285–98. 26611636 10.1038/cr.2015.139 PMC4670995

[12] Wang K, Sun Q, Zhong X, Zeng M, Zeng H, Shi X, et al. Structural Mechanism for GSDMD Targeting by Autoprocessed Caspases in Pyroptosis. Cell. 2020;180:941–955.e20. 32109412 10.1016/j.cell.2020.02.002

[13] Oh C, Spears TJ, Aachoui Y. Inflammasome-mediated pyroptosis in defense against pathogenic bacteria. Immunol Rev. 2025;329:e13408. 39404258 10.1111/imr.13408 PMC11741929

[14] Man SM, Kanneganti TD. Converging roles of caspases in inflammasome activation, cell death and innate immunity. Nat Rev Immunol. 2016;16:7–21. 26655628 10.1038/nri.2015.7 PMC4915362

[15] Xia S, Hollingsworth LR, Wu H. Mechanism and Regulation of Gasdermin-Mediated Cell Death. Cold Spring Harb Perspect Biol. 2020;12:a036400. 10.1101/cshperspect.a036400 PMC7050592 31451512

[16] Privitera G, Rana N, Armuzzi A, Pizarro TT. The gasdermin protein family: emerging roles in gastrointestinal health and disease. Nat Rev Gastroenterol Hepatol. 2023;20:366–87. 36781958 10.1038/s41575-023-00743-w PMC10238632

[17] Feng S, Fox D, Man SM. Mechanisms of Gasdermin Family Members in Inflammasome Signaling and Cell Death. J Mol Biol. 2018;430:3068–80. 29990470 10.1016/j.jmb.2018.07.002

[18] Huston HC, Anderson MJ, Fink SL. Pyroptosis and the cellular consequences of gasdermin pores. Semin Immunol. 2023;69:101803. 37437353 10.1016/j.smim.2023.101803 PMC10530493

[19] Kayagaki N, Kornfeld OS, Lee BL, Stowe IB, O'Rourke K, Li Q, et al. NINJ1 mediates plasma membrane rupture during lytic cell death. Nature. 2021;591:131–6. 33472215 10.1038/s41586-021-03218-7

[20] Chen KW, Broz P. Gasdermins as evolutionarily conserved executors of inflammation and cell death. Nat Cell Biol. 2024;26:1394–406. 39187689 10.1038/s41556-024-01474-z

[21] Man SM, Kanneganti TD. Innate immune sensing of cell death in disease and therapeutics. Nat Cell Biol. 2024;26:1420–33. 39223376 10.1038/s41556-024-01491-y PMC12459733

[22] Carroll SL, Pasare C, Barton GM. Control of adaptive immunity by pattern recognition receptors. Immunity. 2024;57:632–48. 38599163 10.1016/j.immuni.2024.03.014 PMC11037560

[23] Man SM, Jenkins BJ. Context-dependent functions of pattern recognition receptors in cancer. Nat Rev Cancer. 2022;22:397–413. 35355007 10.1038/s41568-022-00462-5

[24] Jastrab JB, Kagan JC. Strategies of bacterial detection by inflammasomes. Cell Chem Biol. 2024;31:835–50. 38636521 10.1016/j.chembiol.2024.03.009 PMC11103797

[25] Man SM, Kanneganti TD. Regulation of inflammasome activation. Immunol Rev. 2015;265:6–21. 25879280 10.1111/imr.12296 PMC4400844

[26] Pandey A, Li Z, Gautam M, Ghosh A, Man SM. Molecular mechanisms of emerging inflammasome complexes and their activation and signaling in inflammation and pyroptosis. Immunol Rev. 2025;329:e13406. 39351983 10.1111/imr.13406 PMC11742652

[27] Mathur A, Hayward JA, Man SM. Molecular mechanisms of inflammasome signaling. J Leukoc Biol. 2018;103:233–57. 28855232 10.1189/jlb.3MR0617-250R

[28] Hayward JA, Mathur A Ngo C, Man SM. Cytosolic Recognition of Microbes and Pathogens: Inflammasomes in Action. Microbiol Mol Biol Rev. 2018;82:e00015–18. 10.1128/MMBR.00015-18 PMC6298609 30209070

[29] Kayagaki N, Warming S, Lamkanfi M, Vande Walle L, Louie S, Dong J, et al. Noncanonical inflammasome activation targets caspase-11. Nature. 2011;479:117–21. 22002608 10.1038/nature10558

[30] Kayagaki N, Wong MT, Stowe IB, Ramani SR, Gonzalez LC, Akashi-Takamura S, et al. Noncanonical inflammasome activation by intracellular LPS independent of TLR4. Science. 2013;341:1246–9. 23887873 10.1126/science.1240248

[31] Martinon F, Burns K, Tschopp J. The inflammasome: a molecular platform triggering activation of inflammatory caspases and processing of proIL-beta. Mol Cell. 2002;10:417–26. 12191486 10.1016/s1097-2765(02)00599-3

[32] Barry K, Murphy C, Mansell A. NLRP1- A CINDERELLA STORY: a perspective of recent advances in NLRP1 and the questions they raise. Commun Biol. 2023;6:1274. 38104185 10.1038/s42003-023-05684-3 PMC10725483

[33] Gong Q, Robinson K, Xu C, Huynh PT, Chong K, Tan E, et al. Structural basis for distinct inflammasome complex assembly by human NLRP1 and CARD8. Nat Commun. 2021;12:188. 33420028 10.1038/s41467-020-20319-5 PMC7794362

[34] Xu Z, Zhou Y, Liu M, Ma H, Sun L, Zahid A, et al. Homotypic CARD-CARD interaction is critical for the activation of NLRP1 inflammasome. Cell Death Dis. 2021;12:57. 33431827 10.1038/s41419-020-03342-8 PMC7801473

[35] Boyden ED, Dietrich WF. Nalp1b controls mouse macrophage susceptibility to anthrax lethal toxin. Nat Genet. 2006;38:240–4. 16429160 10.1038/ng1724

[36] Lilue J, Doran AG, Fiddes IT, Abrudan M, Armstrong J, Bennett R, et al. Sixteen diverse laboratory mouse reference genomes define strain-specific haplotypes and novel functional loci. Nat Genet. 2018;50:1574–83. 30275530 10.1038/s41588-018-0223-8 PMC6205630

[37] Kummer JA, Broekhuizen R, Everett H, Agostini L, Kuijk L, Martinon F, et al. Inflammasome components NALP 1 and 3 show distinct but separate expression profiles in human tissues suggesting a site-specific role in the inflammatory response. J Histochem Cytochem. 2007;55:443–52. 17164409 10.1369/jhc.6A7101.2006

[38] Zhong FL, Mamaï O, Sborgi L, Boussofara L, Hopkins R, Robinson K, et al. Germline NLRP1 Mutations Cause Skin Inflammatory and Cancer Susceptibility Syndromes via Inflammasome Activation. Cell. 2016;167:187–202.e17. 27662089 10.1016/j.cell.2016.09.001

[39] Sand J, Haertel E, Biedermann T, Contassot E, Reichmann E, French LE, et al. Expression of inflammasome proteins and inflammasome activation occurs in human, but not in murine keratinocytes. Cell Death Dis. 2018;9:24. 29348630 10.1038/s41419-017-0009-4 PMC5833864

[40] Cheng YW, Zou ZH, Lou CL, Tong MR, Li JK, Zhu WH, et al. Hippocampal NLRP1 inflammasome mediates anxiety-like behavior in mice with hypothyroidism. Sci Rep. 2025;15:16176. 40346169 10.1038/s41598-025-00979-3 PMC12064720

[41] Sastalla I, Crown D, Masters SL, McKenzie A, Leppla SH, Moayeri M. Transcriptional analysis of the three Nlrp1 paralogs in mice. BMC Genomics. 2013;14:188. 23506131 10.1186/1471-2164-14-188 PMC3641005

[42] D'osualdo A, Weichenberger CX, Wagner RN, Godzik A, Wooley J, Reed JC. CARD8 and NLRP1 undergo autoproteolytic processing through a ZU5-like domain. PLoS One. 2011;6:e27396. 22087307 10.1371/journal.pone.0027396 PMC3210808

[43] Sandstrom A, Mitchell PS, Goers L, Mu EW, Lesser CF, Vance RE. Functional degradation: a mechanism of NLRP1 inflammasome activation by diverse pathogen enzymes. Science. 2019;364:eaau1330. 10.1126/science.aau1330 PMC6532986 30872533

[44] Squires RC, Muehlbauer SM, Brojatsch J. Proteasomes control caspase-1 activation in anthrax lethal toxin-mediated cell killing. J Biol Chem. 2007;282:34260–7. 17878154 10.1074/jbc.M705687200

[45] Wickliffe KE, Leppla SH, Moayeri M. Killing of macrophages by anthrax lethal toxin: involvement of the N-end rule pathway. Cell Microbiol. 2008;10:1352–62. 18266992 10.1111/j.1462-5822.2008.01131.x PMC2500182

[46] Gai K, Okondo MC, Rao SD, Chui AJ, Ball DP, Johnson DC, et al. DPP8/9 inhibitors are universal activators of functional NLRP1 alleles. Cell Death Dis. 2019;10:587. 31383852 10.1038/s41419-019-1817-5 PMC6683174

[47] Okondo MC, Johnson DC, Sridharan R, Go EB, Chui AJ, Wang MS, et al. DPP8 and DPP9 inhibition induces pro-caspase-1-dependent monocyte and macrophage pyroptosis. Nat Chem Biol. 2017;13:46–53. 27820798 10.1038/nchembio.2229 PMC5477230

[48] Hollingsworth LR, Sharif H, Griswold AR, Fontana P, Mintseris J, Dagbay KB, et al. DPP9 sequesters the C-terminus of NLRP1 to repress inflammasome activation. Nature. 2021;592:778–83. 33731932 10.1038/s41586-021-03350-4 PMC8299537

[49] Huang M, Zhang X, Toh GA, Gong Q, Wang J, Han Z, et al. Structural and biochemical mechanisms of NLRP1 inhibition by DPP9. Nature. 2021;592:773–7. 33731929 10.1038/s41586-021-03320-w PMC8081665

[50] Ball DP, Tsamouri LP, Wang AE, Huang HC, Warren CD, Wang Q, et al. Oxidized thioredoxin-1 restrains the NLRP1 inflammasome. Sci Immunol. 2022;7:eabm7200. 36332009 10.1126/sciimmunol.abm7200 PMC9850498

[51] Orth-He EL, Huang HC, Rao SD, Wang Q, Chen Q, O'Mara CM, et al. Protein folding stress potentiates NLRP1 and CARD8 inflammasome activation. Cell Rep. 2023;42:111965. 36649711 10.1016/j.celrep.2022.111965 PMC10042216

[52] Wang Q, Hsiao JC, Yardeny N, Huang HC, O'Mara CM, Orth-He EL, et al. The NLRP1 and CARD8 inflammasomes detect reductive stress. Cell Rep. 2023;42:111966. 36649710 10.1016/j.celrep.2022.111966 PMC9942139

[53] Meade JJ, Stuart S, Neiman-Zenevich J, Krustev C, Girardin SE, Mogridge J. Activation of the NLRP1B inflammasome by caspase-8. Commun Biol. 2024;7:1164. 39289441 10.1038/s42003-024-06882-3 PMC11408587

[54] Feldmeyer L, Keller M, Niklaus G, Hohl D, Werner S, Beer HD. The inflammasome mediates UVB-induced activation and secretion of interleukin-1beta by keratinocytes. Curr Biol. 2007;17:1140–5. 17600714 10.1016/j.cub.2007.05.074

[55] Fenini G, Grossi S, Contassot E, Biedermann T, Reichmann E, French LE, et al. Genome Editing of Human Primary Keratinocytes by CRISPR/Cas9 Reveals an Essential Role of the NLRP1 Inflammasome in UVB Sensing. J Invest Dermatol. 2018;138:2644–52. 30096351 10.1016/j.jid.2018.07.016

[56] Jenster LM, Lange KE, Normann S, vom Hemdt A, Wuerth JD, Schiffelers LDJ, et al. P38 kinases mediate NLRP1 inflammasome activation after ribotoxic stress response and virus infection. J Exp Med. 2023;220:e20220837. 36315050 10.1084/jem.20220837 PMC9623368

[57] Robinson KS, Toh GA, Rozario P, Chua R, Bauernfried S, Sun Z, et al. ZAKalpha-driven ribotoxic stress response activates the human NLRP1 inflammasome. Science. 2022;377:328–35. 35857590 10.1126/science.abl6324 PMC7614315

[58] Robinson KS, Toh GA, Rozario P, Chua R, Bauernfried S, Sun Z, et al. EEF2-inactivating toxins engage the NLRP1 inflammasome and promote epithelial barrier disruption. J Exp Med. 2023;220:e20230104. 37642996 10.1084/jem.20230104 PMC10465324

[59] Robinson KS, Toh GA, Firdaus MJ, Tham KC, Rozario P, Lim CK, et al. Diphtheria toxin activates ribotoxic stress and NLRP1 inflammasome-driven pyroptosis. J Exp Med. 2023;220:e20230105. 37642997 10.1084/jem.20230105 PMC10465786

[60] Tsu BV, Beierschmitt C, Ryan AP, Agarwal R, Mitchell PS, Daugherty MD. Diverse viral proteases activate the NLRP1 inflammasome. Elife. 2021;10:e60609. 33410748 10.7554/eLife.60609 PMC7857732

[61] Bauernfried S, Scherr MJ, Pichlmair A, Duderstadt KE, Hornung V. Human NLRP1 is a sensor for double-stranded RNA. Science. 2021;371:eabd0811. 33243852 10.1126/science.abd0811

[62] Zhou JY, Sarkar MK, Okamura K, Harris JE, Gudjonsson JE, Fitzgerald KA. Activation of the NLRP1 inflammasome in human keratinocytes by the dsDNA mimetic poly(dA:dT). Proc Natl Acad Sci USA. 2023;120:e2213777120. 36693106 10.1073/pnas.2213777120 PMC9945980

[63] Rozario P, Pinilla M, Gorse L, Vind AC, Robinson KS, Toh GA, et al. Mechanistic basis for potassium efflux-driven activation of the human NLRP1 inflammasome. Proc Natl Acad Sci USA. 2024;121:e2309579121. 38175865 10.1073/pnas.2309579121 PMC10786283

[64] Maver A, Lavtar P, Ristić S, Stopinšek S, Simčič S, Hočevar K, et al. Identification of rare genetic variation of NLRP1 gene in familial multiple sclerosis. Sci Rep. 2017;7:3715. 28623311 10.1038/s41598-017-03536-9 PMC5473861

[65] Harapas CR, Robinson KS, Lay K, Wong J, Moreno Traspas R, Nabavizadeh N, et al. DPP9 deficiency: An inflammasomopathy that can be rescued by lowering NLRP1/IL-1 signaling. Sci Immunol. 2022;7:eabi4611. 36112693 10.1126/sciimmunol.abi4611 PMC9844213

[66] Docherty CA, Fernando AJ, Rosli S, Lam M, Dolle RE, Navia MA, et al. A novel dual NLRP1 and NLRP3 inflammasome inhibitor for the treatment of inflammatory diseases. Clin Transl Immunol. 2023;12:e1455. 10.1002/cti2.1455 PMC10288073 37360982

[67] Zhang Z, Shibata T, Fujimura A, Kitaura J, Miyake K, Ohto U, et al. Structural basis for thioredoxin-mediated suppression of NLRP1 inflammasome. Nature. 2023;622:188–94. 37704723 10.1038/s41586-023-06532-4

[68] Guarda G, Zenger M, Yazdi AS, Schroder K, Ferrero I, Menu P, et al. Differential expression of NLRP3 among hematopoietic cells. J Immunol. 2011;186:2529–34. 21257968 10.4049/jimmunol.1002720

[69] Lebreton F, Berishvili E, Parnaud G, Rouget C, Bosco D, Berney T, et al. NLRP3 inflammasome is expressed and regulated in human islets. Cell Death Dis. 2018;9:726. 29941940 10.1038/s41419-018-0764-x PMC6018156

[70] Youm YH, Kanneganti TD, Vandanmagsar B, Zhu X, Ravussin A, Adijiang A, et al. The Nlrp3 inflammasome promotes age-related thymic demise and immunosenescence. Cell Rep. 2012;1:56–68. 22832107 10.1016/j.celrep.2011.11.005 PMC3883512

[71] Aganna E, Martinon F, Hawkins PN, Ross JB, Swan DC, Booth DR, et al. Association of mutations in the NALP3/CIAS1/PYPAF1 gene with a broad phenotype including recurrent fever, cold sensitivity, sensorineural deafness, and AA amyloidosis. Arthritis Rheum. 2002;46:2445–52. 12355493 10.1002/art.10509

[72] Hoffman HM, Mueller JL, Broide DH, Wanderer AA, Kolodner RD. Mutation of a new gene encoding a putative pyrin-like protein causes familial cold autoinflammatory syndrome and Muckle-Wells syndrome. Nat Genet. 2001;29:301–5. 11687797 10.1038/ng756 PMC4322000

[73] Feldmann J, Prieur AM, Quartier P, Berquin P, Certain S, Cortis E, et al. Chronic infantile neurological cutaneous and articular syndrome is caused by mutations in CIAS1, a gene highly expressed in polymorphonuclear cells and chondrocytes. Am J Hum Genet. 2002;71:198–203. 12032915 10.1086/341357 PMC384980

[74] Agostini L, Martinon F, Burns K, McDermott MF, Hawkins PN, Tschopp J. NALP3 forms an IL-1beta-processing inflammasome with increased activity in Muckle-Wells autoinflammatory disorder. Immunity. 2004;20:319–25. 15030775 10.1016/s1074-7613(04)00046-9

[75] Manji GA, Wang L, Geddes BJ, Brown M, Merriam S, Al-Garawi A, et al. PYPAF1, a PYRIN-containing Apaf1-like protein that assembles with ASC and regulates activation of NF-kappa B. J Biol Chem. 2002;277:11570–5. 11786556 10.1074/jbc.M112208200

[76] Mariathasan S, Weiss DS, Newton K, McBride J, O'Rourke K, Roose-Girma M, et al. Cryopyrin activates the inflammasome in response to toxins and ATP. Nature. 2006;440:228–32. 16407890 10.1038/nature04515

[77] Kanneganti TD, Ozören N, Body-Malapel M, Amer A, Park JH, Franchi L, et al. Bacterial RNA and small antiviral compounds activate caspase-1 through cryopyrin/Nalp3. Nature. 2006;440:233–6. 16407888 10.1038/nature04517

[78] Kanneganti TD, Body-Malapel M, Amer A, Park JH, Whitfield J, Franchi L, et al. Critical role for Cryopyrin/Nalp3 in activation of caspase-1 in response to viral infection and double-stranded RNA. J Biol Chem. 2006;281:36560–8. 17008311 10.1074/jbc.M607594200

[79] Martinon F, Pétrilli V, Mayor A, Tardivel A, Tschopp J. Gout-associated uric acid crystals activate the NALP3 inflammasome. Nature. 2006;440:237–41. 16407889 10.1038/nature04516

[80] Bauernfeind FG, Horvath G, Stutz A, Alnemri ES, MacDonald K, Speert D, et al. Cutting edge: NF-kappaB activating pattern recognition and cytokine receptors license NLRP3 inflammasome activation by regulating NLRP3 expression. J Immunol. 2009;183:787–91. 19570822 10.4049/jimmunol.0901363 PMC2824855

[81] Franchi L, Eigenbrod T, Nunez G. Cutting edge: TNF-alpha mediates sensitization to ATP and silica via the NLRP3 inflammasome in the absence of microbial stimulation. J Immunol. 2009;183:792–6. 19542372 10.4049/jimmunol.0900173 PMC2754237

[82] Zhang Z, Meszaros G, He WT, Xu Y, de Fatima Magliarelli H, Mailly L, et al. Protein kinase D at the Golgi controls NLRP3 inflammasome activation. J Exp Med. 2017;214:2671–93. 28716882 10.1084/jem.20162040 PMC5584123

[83] Zheng S, Que X, Wang S, Zhou Q, Xing X, Chen L, et al. ZDHHC5-mediated NLRP3 palmitoylation promotes NLRP3-NEK7 interaction and inflammasome activation. Mol Cell. 2023;83:4570–4585.e7. 38092000 10.1016/j.molcel.2023.11.015

[84] Qin Y, Li Q, Liang W, Yan R, Tong L, Jia M, et al. TRIM28 SUMOylates and stabilizes NLRP3 to facilitate inflammasome activation. Nat Commun. 2021;12:4794. 34373456 10.1038/s41467-021-25033-4 PMC8352945

[85] Hang Y, Tan L, Chen Q, Liu Q, Jin Y. E3 ubiquitin ligase TRIM24 deficiency promotes NLRP3/caspase-1/IL-1beta-mediated pyroptosis in endometriosis. Cell Biol Int. 2021;45:1561–70. 33724611 10.1002/cbin.11592

[86] Shen J, Wu Q, Liang T, Zhang J, Bai J, Yuan M, et al. TRIM40 inhibits IgA1-induced proliferation of glomerular mesangial cells by inactivating NLRP3 inflammasome through ubiquitination. Mol Immunol. 2021;140:225–32. 34763147 10.1016/j.molimm.2021.10.012

[87] Song H, Liu B, Huai W, Yu Z, Wang W, Zhao J, et al. The E3 ubiquitin ligase TRIM31 attenuates NLRP3 inflammasome activation by promoting proteasomal degradation of NLRP3. Nat Commun. 2016;7:13727. 27929086 10.1038/ncomms13727 PMC5155141

[88] Pereira M, Tourlomousis P, Wright J, P Monie T, Bryant CE. CARD9 negatively regulates NLRP3-induced IL-1beta production on Salmonella infection of macrophages. Nat Commun. 2016;7:12874. 27670879 10.1038/ncomms12874 PMC5052644

[89] Liu Q, Yan L, Wu T, Wu Q, Ke B, Shen W. Peli1, regulated by m(6)A modification, suppresses NLRP3 inflammasome activation in atherosclerosis by inhibiting YB-1. Commun Biol. 2025;8:457. 40102597 10.1038/s42003-025-07839-w PMC11920095

[90] Cui H, Banerjee S, Xie N, Dey T, Liu RM, Sanders YY, et al. MafB regulates NLRP3 inflammasome activation by sustaining p62 expression in macrophages. Commun Biol. 2023;6:1047. 37845329 10.1038/s42003-023-05426-5 PMC10579372

[91] Jing W, Lo Pilato J, Kay C, Man SM. Activation mechanisms of inflammasomes by bacterial toxins. Cell Microbiol. 2021;23:13309 p. 10.1111/cmi.13309 33426791

[92] Xue Y, Enosi Tuipulotu D, Tan WH, Kay C, Man SM. Emerging Activators and Regulators of Inflammasomes and Pyroptosis. Trends Immunol. 2019;40:1035–52. 31662274 10.1016/j.it.2019.09.005

[93] Perregaux D, Gabel CA. Interleukin-1 beta maturation and release in response to ATP and nigericin. Evidence that potassium depletion mediated by these agents is a necessary and common feature of their activity. J Biol Chem. 1994;269:15195–203. 8195155

[94] Pétrilli V, Papin S, Dostert C, Mayor A, Martinon F, Tschopp J. Activation of the NALP3 inflammasome is triggered by low intracellular potassium concentration. Cell Death Differ. 2007;14:1583–9. 17599094 10.1038/sj.cdd.4402195

[95] Muñoz-Planillo R, Kuffa P, Martínez-Colón G, Smith BL, Rajendiran TM, Núñez G. K(+) efflux is the common trigger of NLRP3 inflammasome activation by bacterial toxins and particulate matter. Immunity. 2013;38:1142–53. 23809161 10.1016/j.immuni.2013.05.016 PMC3730833

[96] Hornung V, Bauernfeind F, Halle A, Samstad EO, Kono H, Rock KL, et al. Silica crystals and aluminum salts activate the NALP3 inflammasome through phagosomal destabilization. Nat Immunol. 2008;9:847–56. 18604214 10.1038/ni.1631 PMC2834784

[97] Mathur A, Kay C, Xue Y, Pandey A, Lee J, Jing W, et al. Clostridium perfringens virulence factors are nonredundant activators of the NLRP3 inflammasome. EMBO Rep. 2023;24:e54600. 37073791 10.15252/embr.202254600 PMC10240202

[98] Groß CJ, Mishra R, Schneider KS, Médard G, Wettmarshausen J, Dittlein DC, et al. K(+) Efflux-Independent NLRP3 Inflammasome Activation by Small Molecules Targeting Mitochondria. Immunity. 2016;45:761–73. 27692612 10.1016/j.immuni.2016.08.010

[99] Zhou R, Yazdi AS, Menu P, Tschopp J. A role for mitochondria in NLRP3 inflammasome activation. Nature. 2011;469:221–5. 21124315 10.1038/nature09663

[100] Qu Y, Misaghi S, Newton K, Gilmour LL, Louie S, Cupp JE, et al. Pannexin-1 is required for ATP release during apoptosis but not for inflammasome activation. J Immunol. 2011;186:6553–61. 21508259 10.4049/jimmunol.1100478

[101] Di A, Xiong S, Ye Z, Malireddi R, Kometani S, Zhong M, et al. The TWIK2 Potassium Efflux Channel in Macrophages Mediates NLRP3 Inflammasome-Induced Inflammation. Immunity. 2018;49:56–65.e4. 29958799 10.1016/j.immuni.2018.04.032 PMC6051907

[102] Fox D, Mathur A, Xue Y, Liu Y, Tan WH, Feng S, et al. Bacillus cereus nonhaemolytic enterotoxin activates the NLRP3 inflammasome. Nat Commun. 2020;11:760. 32029733 10.1038/s41467-020-14534-3 PMC7005308

[103] Mathur A, Feng S, Hayward JA, Ngo C, Fox D, Atmosukarto II, et al. A multicomponent toxin from Bacillus cereus incites inflammation and shapes host outcome via the NLRP3 inflammasome. Nat Microbiol. 2019;4:362–74. 30531979 10.1038/s41564-018-0318-0 PMC7685251

[104] He Y, Zeng MY, Yang D, Motro B, Núñez G. NEK7 is an essential mediator of NLRP3 activation downstream of potassium efflux. Nature. 2016;530:354–7. 26814970 10.1038/nature16959 PMC4810788

[105] Schmid-Burgk JL, Chauhan D, Schmidt T, Ebert TS, Reinhardt J, Endl E, et al. A Genome-wide CRISPR (Clustered Regularly Interspaced Short Palindromic Repeats) Screen Identifies NEK7 as an Essential Component of NLRP3 Inflammasome Activation. J Biol Chem. 2016;291:103–9. 26553871 10.1074/jbc.C115.700492 PMC4697147

[106] Shi H, Wang Y, Li X, Zhan X, Tang M, Fina M, et al. NLRP3 activation and mitosis are mutually exclusive events coordinated by NEK7, a new inflammasome component. Nat Immunol. 2016;17:250–8. 26642356 10.1038/ni.3333 PMC4862588

[107] Sharif H, Wang L, Wang WL, Magupalli VG, Andreeva L, Qiao Q, et al. Structural mechanism for NEK7-licensed activation of NLRP3 inflammasome. Nature. 2019;570:338–43. 31189953 10.1038/s41586-019-1295-z PMC6774351

[108] Xiao L, Magupalli VG, Wu H. Cryo-EM structures of the active NLRP3 inflammasome disc. Nature. 2023;613:595–600. 36442502 10.1038/s41586-022-05570-8 PMC10091861

[109] Brinkschulte R, Fußhöller DM, Hoss F, Rodríguez-Alcázar JF, Lauterbach MA, Kolbe CC, et al. ATP-binding and hydrolysis of human NLRP3. Commun Biol. 2022;5:1176. 36329210 10.1038/s42003-022-04120-2 PMC9633759

[110] Jiang H, He H, Chen Y, Huang W, Cheng J, Ye J, et al. Identification of a selective and direct NLRP3 inhibitor to treat inflammatory disorders. J Exp Med. 2017;214:3219–38. 29021150 10.1084/jem.20171419 PMC5679172

[111] Dick MS, Sborgi L, Rühl S, Hiller S, Broz P. ASC filament formation serves as a signal amplification mechanism for inflammasomes. Nat Commun. 2016;7:11929. 27329339 10.1038/ncomms11929 PMC4917984

[112] Oroz J, Barrera-Vilarmau S, Alfonso C, Rivas G, de Alba E. ASC Pyrin Domain Self-associates and Binds NLRP3 Protein Using Equivalent Binding Interfaces. J Biol Chem. 2016;291:19487–501. 27432880 10.1074/jbc.M116.741082 PMC5016686

[113] Liu Y, Zhai H, Alemayehu H, Boulanger J, Hopkins LJ, Borgeaud AC, et al. Cryo-electron tomography of NLRP3-activated ASC complexes reveals organelle colocalization. Nat Commun. 2023;14:7246. 37945612 10.1038/s41467-023-43180-8 PMC10636019

[114] Schmidt FI, Lu A, Chen JW, Ruan J, Tang C, Wu H, et al. A single domain antibody fragment that recognizes the adaptor ASC defines the role of ASC domains in inflammasome assembly. J Exp Med. 2016;213:771–90. 27069117 10.1084/jem.20151790 PMC4854733

[115] Xu J, Zhang L, Duan Y, Sun F, Odeh N, He Y, et al. NEK7 phosphorylation amplifies NLRP3 inflammasome activation downstream of potassium efflux and gasdermin D. Sci Immunol. 2025;10:eadl2993. 39752537 10.1126/sciimmunol.adl2993 PMC12020992

[116] Lee GS, Subramanian N, Kim AI, Aksentijevich I, Goldbach-Mansky R, Sacks DB, et al. The calcium-sensing receptor regulates the NLRP3 inflammasome through Ca2+ and cAMP. Nature. 2012;492:123–7. 23143333 10.1038/nature11588 PMC4175565

[117] Murakami T, Ockinger J, Yu J, Byles V, McColl A, Hofer AM, et al. Critical role for calcium mobilization in activation of the NLRP3 inflammasome. Proc Natl Acad Sci USA. 2012;109:11282–7. 22733741 10.1073/pnas.1117765109 PMC3396518

[118] Rossol M, Pierer M, Raulien N, Quandt D, Meusch U, Rothe K, et al. Extracellular Ca2+ is a danger signal activating the NLRP3 inflammasome through G protein-coupled calcium sensing receptors. Nat Commun. 2012;3:1329. 23271661 10.1038/ncomms2339 PMC3535422

[119] Jäger E, Murthy S, Schmidt C, Hahn M, Strobel S, Peters A, et al. Calcium-sensing receptor-mediated NLRP3 inflammasome response to calciprotein particles drives inflammation in rheumatoid arthritis. Nat Commun. 2020;11:4243. 32843625 10.1038/s41467-020-17749-6 PMC7447633

[120] Huang LS, Anas M, Xu J, Zhou B, Toth PT, Krishnan Y, et al. Endosomal trafficking of two-pore K(+) efflux channel TWIK2 to plasmalemma mediates NLRP3 inflammasome activation and inflammatory injury. Elife. 2023;12:e83842. 37158595 10.7554/eLife.83842 PMC10202452

[121] Green JP, Yu S, Martín-Sánchez F, Pelegrin P, Lopez-Castejon G, Lawrence CB, et al. Chloride regulates dynamic NLRP3-dependent ASC oligomerization and inflammasome priming. Proc Natl Acad Sci USA. 2018;115:E9371–E9380. 30232264 10.1073/pnas.1812744115 PMC6176575

[122] Tang T, Lang X, Xu C, Wang X, Gong T, Yang Y, et al. CLICs-dependent chloride efflux is an essential and proximal upstream event for NLRP3 inflammasome activation. Nat Commun. 2017;8:202. 28779175 10.1038/s41467-017-00227-x PMC5544706

[123] Mayes-Hopfinger L, Enache A, Xie J, Huang CL, Köchl R, Tybulewicz V, et al. Chloride sensing by WNK1 regulates NLRP3 inflammasome activation and pyroptosis. Nat Commun. 2021;12:4546. 34315884 10.1038/s41467-021-24784-4 PMC8316491

[124] Pandey A, Shen C, Feng S, Man SM. Cell biology of inflammasome activation. Trends Cell Biol. 2021;31:924–39. 34284921 10.1016/j.tcb.2021.06.010

[125] Shimada K, Crother TR, Karlin J, Dagvadorj J, Chiba N, Chen S, et al. Oxidized mitochondrial DNA activates the NLRP3 inflammasome during apoptosis. Immunity. 2012;36:401–14. 22342844 10.1016/j.immuni.2012.01.009 PMC3312986

[126] Zhong Z, Liang S, Sanchez-Lopez E, He F, Shalapour S, Lin XJ, et al. New mitochondrial DNA synthesis enables NLRP3 inflammasome activation. Nature. 2018;560:198–203. 30046112 10.1038/s41586-018-0372-z PMC6329306

[127] Cabral A, Cabral JE, Wang A, Zhang Y, Liang H, Nikbakht D, et al. Differential Binding of NLRP3 to nonoxidized and Ox-mtDNA mediates NLRP3 Inflammasome Activation. Commun Biol. 2023;6:578. 37253813 10.1038/s42003-023-04817-y PMC10229695

[128] Vince JE, De Nardo D, Gao W, Vince AJ, Hall C, McArthur K, et al. The Mitochondrial Apoptotic Effectors BAX/BAK Activate Caspase-3 and -7 to Trigger NLRP3 Inflammasome and Caspase-8 Driven IL-1beta Activation. Cell Rep. 2018;25:2339–2353.e4. 30485804 10.1016/j.celrep.2018.10.103

[129] Thi Tran U, Kitami T. Niclosamide activates the NLRP3 inflammasome by intracellular acidification and mitochondrial inhibition. Commun Biol. 2019;2:2. 30740538 10.1038/s42003-018-0244-y PMC6318214

[130] Zhou R, Tardivel A, Thorens B, Choi I, Tschopp J. Thioredoxin-interacting protein links oxidative stress to inflammasome activation. Nat Immunol. 2010;11:136–40. 20023662 10.1038/ni.1831

[131] Saller BS, Wöhrle S, Fischer L, Dufossez C, Ingerl IL, Kessler S, et al. Acute suppression of mitochondrial ATP production prevents apoptosis and provides an essential signal for NLRP3 inflammasome activation. Immunity. 2025;58:90–107.e11. 39571574 10.1016/j.immuni.2024.10.012

[132] Chen J, Chen ZJ. PtdIns4P on dispersed trans-Golgi network mediates NLRP3 inflammasome activation. Nature. 2018;564:71–76. 30487600 10.1038/s41586-018-0761-3 PMC9402428

[133] Xiao N, Kogishi A, Radochonski L, Lei Y, Chen J. Type A cholesterol-dependent cytolysins translocate to the trans-Golgi network for NLRP3 inflammasome activation. Nat Immunol. 2025 10.1038/s41590-025-02277-6. Online ahead of print. 10.1038/s41590-025-02277-6 PMC12443345 40913097

[134] Boršić E, Ramuta TŽ, Orehek S, Kreft ME, Geyer M, Jerala R, et al. Clustering of NLRP3 induced by membrane or protein scaffolds promotes inflammasome assembly. Nat Commun. 2025;16:4887. 40425567 10.1038/s41467-025-60277-4 PMC12117088

[135] Bronner DN, Abuaita BH, Chen X, Fitzgerald KA, Nuñez G, He Y, et al. Endoplasmic Reticulum Stress Activates the Inflammasome via NLRP3- and Caspase-2-Driven Mitochondrial Damage. Immunity. 2015;43:451–62. 26341399 10.1016/j.immuni.2015.08.008 PMC4582788

[136] Lee B, Hoyle C, Wellens R, Green JP, Martin-Sanchez F, Williams DM, et al. Disruptions in endocytic traffic contribute to the activation of the NLRP3 inflammasome. Sci Signal. 2023;16:eabm7134. 36809026 10.1126/scisignal.abm7134

[137] Zhang Z, Venditti R, Ran L, Liu Z, Vivot K, Schürmann A, et al. Distinct changes in endosomal composition promote NLRP3 inflammasome activation. Nat Immunol. 2023;24:30–41. 36443515 10.1038/s41590-022-01355-3 PMC9810532

[138] Guo C, Chi Z, Jiang D, Xu T, Yu W, Wang Z, et al. Cholesterol Homeostatic Regulator SCAP-SREBP2 Integrates NLRP3 Inflammasome Activation and Cholesterol Biosynthetic Signaling in Macrophages. Immunity. 2018;49:842–856 e7. 30366764 10.1016/j.immuni.2018.08.021

[139] Halle A, Hornung V, Petzold GC, Stewart CR, Monks BG, Reinheckel T, et al. The NALP3 inflammasome is involved in the innate immune response to amyloid-b. eta Nat Immunol. 2008;9:857–65. 10.1038/ni.1636 PMC3101478 18604209

[140] Duewell P, Kono H, Rayner KJ, Sirois CM, Vladimer G, Bauernfeind FG, et al. the NLRP3 inflammasome are required for atherogenesis and activated by cholesterol crystals. Nature. 2010;464:1357–61. 20428172 10.1038/nature08938 PMC2946640

[141] Lauterbach MA, Saavedra V, Mangan M, Penno A, Thiele C, Latz E, et al. 1-Deoxysphingolipids cause autophagosome and lysosome accumulation and trigger NLRP3 inflammasome activation. Autophagy. 2021;17:1947–61. 32835606 10.1080/15548627.2020.1804677 PMC8386713

[142] Samir P, Kesavardhana S, Patmore DM, Gingras S, Malireddi R, Karki R, et al. DDX3X acts as a live-or-die checkpoint in stressed cells by regulating NLRP3 inflammasome. Nature. 2019;573:590–4. 31511697 10.1038/s41586-019-1551-2 PMC6980284

[143] Gaidt MM, Ebert TS, Chauhan D, Schmidt T, Schmid-Burgk JL, Rapino F, et al. Human Monocytes Engage an Alternative Inflammasome Pathway. Immunity. 2016;44:833–46. 27037191 10.1016/j.immuni.2016.01.012

[144] Unterberger S, Mullen L, Flint MS, Sacre S. Multiple TLRs elicit alternative NLRP3 inflammasome activation in primary human monocytes independent of RIPK1 kinase activity. Front Immunol. 2023;14:1092799. 37954581 10.3389/fimmu.2023.1092799 PMC10639122

[145] Gao Y, Yu S, Chen M, Wang X, Pan L, Wei B, et al. cFLIP(S) regulates alternative NLRP3 inflammasome activation in human monocytes. Cell Mol Immunol. 2023;20:1203–15. 37591930 10.1038/s41423-023-01077-y PMC10541859

[146] Franchi L, Eigenbrod T, Muñoz-Planillo R, Ozkurede U, Kim YG, Arindam C, et al. Cytosolic double-stranded RNA activates the NLRP3 inflammasome via MAVS-induced membrane permeabilization and K+ efflux. J Immunol. 2014;193:4214–22. 25225670 10.4049/jimmunol.1400582 PMC4185247

[147] Hise AG, Tomalka J, Ganesan S, Patel K, Hall BA, Brown GD, et al. An essential role for the NLRP3 inflammasome in host defense against the human fungal pathogen Candida albicans. Cell Host Microbe. 2009;5:487–97. 19454352 10.1016/j.chom.2009.05.002 PMC2824856

[148] Kasper L, König A, Koenig PA, Gresnigt MS, Westman J, Drummond RA, et al. The fungal peptide toxin Candidalysin activates the NLRP3 inflammasome and causes cytolysis in mononuclear phagocytes. Nat Commun. 2018;9:4260. 30323213 10.1038/s41467-018-06607-1 PMC6189146

[149] Marina-García N, Franchi L, Kim YG, Miller D, McDonald C, Boons GJ, et al. Pannexin-1-mediated intracellular delivery of muramyl dipeptide induces caspase-1 activation via cryopyrin/NLRP3 independently of Nod2. J Immunol. 2008;180:4050–7. 18322214 10.4049/jimmunol.180.6.4050

[150] Sander LE, Davis MJ, Boekschoten MV, Amsen D, Dascher CC, Ryffel B, et al. Detection of prokaryotic mRNA signifies microbial viability and promotes immunity. Nature. 2011;474:385–9. 21602824 10.1038/nature10072 PMC3289942

[151] Thomas PG, Dash P, Aldridge JR, Ellebedy AH, Reynolds C, Funk AJ, et al. The intracellular sensor NLRP3 mediates key innate and healing responses to influenza A virus via the regulation of caspase-1. Immunity. 2009;30:566–75. 19362023 10.1016/j.immuni.2009.02.006 PMC2765464

[152] Yao X, Zhang C, Xing Y, Xue G, Zhang Q, Pan F, et al. Remodeling of the gut microbiota by hyperactive NLRP3 induces regulatory T cells to maintain homeostasis. Nat Commun. 2017;8:1896. 29196621 10.1038/s41467-017-01917-2 PMC5711854

[153] Harder J, Franchi L, Muñoz-Planillo R, Park JH, Reimer T, Núñez G. Activation of the Nlrp3 inflammasome by Streptococcus pyogenes requires streptolysin O and NF-kappa B activation but proceeds independently of TLR signaling and P2X7 receptor. J Immunol. 2009;183:5823–9. 19812205 10.4049/jimmunol.0900444 PMC2765568

[154] McNeela EA, Burke A, Neill DR, Baxter C, Fernandes VE, Ferreira D, et al. Pneumolysin activates the NLRP3 inflammasome and promotes proinflammatory cytokines independently of TLR4. PLoS Pathog. 2010;6:e1001191. 21085613 10.1371/journal.ppat.1001191 PMC2978728

[155] Jing W, Pilato JL, Kay C, Feng S, Tuipulotu DE, Mathur A, et al. Clostridium septicum alpha-toxin activates the NLRP3 inflammasome by engaging GPI-anchored proteins. Sci Immunol. 2022;7:eabm1803. 35594341 10.1126/sciimmunol.abm1803

[156] Broz P, Newton K, Lamkanfi M, Mariathasan S, Dixit VM, Monack DM. Redundant roles for inflammasome receptors NLRP3 and NLRC4 in host defense against Salmonella. J Exp Med. 2010;207:1745–55. 20603313 10.1084/jem.20100257 PMC2916133

[157] Man SM, Hopkins LJ, Nugent E, Cox S, Glück IM, Tourlomousis P, et al. Inflammasome activation causes dual recruitment of NLRC4 and NLRP3 to the same macromolecular complex. Proc Natl Acad Sci USA. 2014;111:7403–8. 24803432 10.1073/pnas.1402911111 PMC4034195

[158] Gram AM, Wright JA, Pickering RJ, Lam NL, Booty LM, Webster SJ, et al. Salmonella Flagellin Activates NAIP/NLRC4 and Canonical NLRP3 Inflammasomes in Human Macrophages. J Immunol. 2021;206:631–40. 33380493 10.4049/jimmunol.2000382 PMC7812056

[159] Brodsky IE, Palm NW, Sadanand S, Ryndak MB, Sutterwala FS, Flavell RA, et al. A Yersinia effector protein promotes virulence by preventing inflammasome recognition of the type III secretion system. Cell Host Microbe. 2010;7:376–87. 20478539 10.1016/j.chom.2010.04.009 PMC2883865

[160] Sutterwala FS, Mijares LA, Li L, Ogura Y, Kazmierczak BI, Flavell RA. Immune recognition of Pseudomonas aeruginosa mediated by the IPAF/NLRC4 inflammasome. J Exp Med. 2007;204:3235–45. 18070936 10.1084/jem.20071239 PMC2150987

[161] Chen D, Wu L, Liu X, Wang Q, Gui S, Bao L, et al. Helicobacter pylori CagA mediated mitophagy to attenuate the NLRP3 inflammasome activation and enhance the survival of infected cells. Sci Rep. 2024;14:21648. 39289452 10.1038/s41598-024-72534-5 PMC11408507

[162] Allen IC, Scull MA, Moore CB, Holl EK, McElvania-TeKippe E, Taxman DJ, et al. The NLRP3 inflammasome mediates in vivo innate immunity to influenza A virus through recognition of viral RNA. Immunity. 2009;30:556–65. 19362020 10.1016/j.immuni.2009.02.005 PMC2803103

[163] Kuriakose T, Man SM, Malireddi RK, Karki R, Kesavardhana S, Place DE, et al. ZBP1/DAI is an innate sensor of influenza virus triggering the NLRP3 inflammasome and programmed cell death pathways. Sci Immunol. 2016;1:aag2045. 27917412 10.1126/sciimmunol.aag2045 PMC5131924

[164] Ding X, Lei Q, Li T, Li L, Qin B. Hepatitis B core antigen can regulate NLRP3 inflammasome pathway in HepG2 cells. J Med Virol. 2019;91:1528–36. 31017673 10.1002/jmv.25490

[165] Xie WH, Ding J, Xie XX, Yang XH, Wu XF, Chen ZX, et al. Hepatitis B virus X protein promotes liver cell pyroptosis under oxidative stress through NLRP3 inflammasome activation. Inflamm Res. 2020;69:683–96. 32347316 10.1007/s00011-020-01351-z PMC7261280

[166] Kaushik DK, Gupta M, Kumawat KL, Basu A. NLRP3 inflammasome: key mediator of neuroinflammation in murine Japanese encephalitis. PLoS One. 2012;7:e32270. 22393394 10.1371/journal.pone.0032270 PMC3290554

[167] Ermler ME, Traylor Z, Patel K, Schattgen SA, Vanaja SK, Fitzgerald KA, et al. Rift Valley fever virus infection induces activation of the NLRP3 inflammasome. Virology. 2014;449:174–80. 24418550 10.1016/j.virol.2013.11.015 PMC3951897

[168] Ito M, Yanagi Y, Ichinohe T. Encephalomyocarditis virus viroporin 2B activates NLRP3 inflammasome. PLoS Pathog. 2012;8:e1002857. 22916014 10.1371/journal.ppat.1002857 PMC3415442

[169] Zhi X, Zhang Y, Sun S, Zhang Z, Dong H, Luo X, et al. NLRP3 inflammasome activation by Foot-and-mouth disease virus infection mainly induced by viral RNA and nonstructural protein 2B. RNA Biol. 2020;17:335–49. 31840571 10.1080/15476286.2019.1700058 PMC6999635

[170] de Castro-Jorge LA, de Carvalho R, Klein TM, Hiroki CH, Lopes AH, Guimarães RM, et al. The NLRP3 inflammasome is involved with the pathogenesis of Mayaro virus. PLoS Pathog. 2019;15:e1007934. 31479495 10.1371/journal.ppat.1007934 PMC6743794

[171] Nour AM, Reichelt M, Ku CC, Ho MY, Heineman TC, Arvin AM. Varicella-zoster virus infection triggers formation of an interleukin-1beta (IL-1beta)-processing inflammasome complex. J Biol Chem. 2011;286:17921–33. 21385879 10.1074/jbc.M110.210575 PMC3093867

[172] Shrivastava G, Visoso-Carvajal G, Garcia-Cordero J, Leon-Juarez M, Chavez-Munguia B, Lopez T, et al. Dengue Virus Serotype 2 and Its Non-Structural Proteins 2A and 2B Activate NLRP3 Inflammasome. Front Immunol. 2020;11:352. 32210961 10.3389/fimmu.2020.00352 PMC7076137

[173] Wang W, Li G, De WU, Luo Z, Pan P, Tian M, et al. Zika virus infection induces host inflammatory responses by facilitating NLRP3 inflammasome assembly and interleukin-1beta secretion. Nat Commun. 2018;9:106. 29317641 10.1038/s41467-017-02645-3 PMC5760693

[174] Barlan AU, Danthi P, Wiethoff CM. Lysosomal localization and mechanism of membrane penetration influence nonenveloped virus activation of the NLRP3 inflammasome. Virology. 2011;412:306–14. 21315400 10.1016/j.virol.2011.01.019 PMC3060956

[175] Chakrabarti A, Banerjee S, Franchi L, Loo YM, Gale MJR, Núñez G, et al. RNase L activates the NLRP3 inflammasome during viral infections. Cell Host Microbe. 2015;17:466–77. 25816776 10.1016/j.chom.2015.02.010 PMC4393362

[176] Li J, Hu L, Liu Y, Huang L, Mu Y, Cai X, et al. DDX19A Senses Viral RNA and Mediates NLRP3-Dependent Inflammasome Activation. J Immunol. 2015;195:5732–49. 26538395 10.4049/jimmunol.1501606

[177] Moriyama M, Nagai M, Maruzuru Y, Koshiba T, Kawaguchi Y, Ichinohe T. Influenza Virus-Induced Oxidized DNA Activates Inflammasomes. iScience. 2020;23:101270. 32592999 10.1016/j.isci.2020.101270 PMC7293844

[178] Poeck H, Bscheider M, Gross O, Finger K, Roth S, Rebsamen M, et al. Recognition of RNA virus by RIG-I results in activation of CARD9 and inflammasome signaling for interleukin 1 beta production. Nat Immunol. 2010;11:63–9. 19915568 10.1038/ni.1824

[179] Wang X, Jiang W, Yan Y, Gong T, Han J, Tian Z, et al. RNA viruses promote activation of the NLRP3 inflammasome through a RIP1-RIP3-DRP1 signaling pathway. Nat Immunol. 2014;15:1126–33. 25326752 10.1038/ni.3015

[180] Niu J, Cui M, Yang X, Li J, Yao Y, Guo Q, et al. Microbiota-derived acetate enhances host antiviral response via NLRP3. Nat Commun. 2023;14:642. 36746963 10.1038/s41467-023-36323-4 PMC9901394

[181] Gross O, Poeck H, Bscheider M, Dostert C, Hannesschläger N, Endres S, et al. Syk kinase signaling couples to the Nlrp3 inflammasome for anti-fungal host defense. Nature. 2009;459:433–6. 19339971 10.1038/nature07965

[182] Karki R, Man SM, Malireddi R, Gurung P, Vogel P, Lamkanfi M, et al. Concerted activation of the AIM2 and the NLRP3 inflammasome orchestrates host protection against Aspergillus infection. Cell Host Microbe. 2015;17:357–68. 25704009 10.1016/j.chom.2015.01.006 PMC4359672

[183] Saïd-Sadier N, Padilla E, Langsley G, Ojcius DM. Aspergillus fumigatus stimulates the NLRP3 inflammasome through a pathway requiring ROS production and the Syk tyrosine kinase. PLoS One. 2010;5:e10008. 20368800 10.1371/journal.pone.0010008 PMC2848854

[184] Lu S, Li Z, Wu J, Tang Y, Zhang Q, Xi L. NLRP3 inflammasome activation contributes to fungal clearance and lung injury during Talaromyces marneffei infection. Micro Pathog. 2023;181:106169. 10.1016/j.micpath.2023.106169 37257668

[185] Lamkanfi M, Malireddi RK, Kanneganti TD. Fungal zymosan and mannan activate the cryopyrin inflammasome. J Biol Chem. 2009;284:20574–81. 19509280 10.1074/jbc.M109.023689 PMC2742822

[186] Briard B, Fontaine T, Samir P, Place DE, Muszkieta L, Malireddi R, et al. Galactosaminogalactan activates the inflammasome to provide host protection. Nature. 2020;588:688–92. 33268895 10.1038/s41586-020-2996-z PMC8086055

[187] Cheng SC, van de Veerdonk FL, Lenardon M, Stoffels M, Plantinga T, Smeekens S, et al. The dectin-1/inflammasome pathway is responsible for the induction of protective T-helper 17 responses that discriminate between yeasts and hyphae of Candida albicans. J Leukoc Biol. 2011;90:357–66. 21531876 10.1189/jlb.1210702 PMC3513931

[188] Silva RL, Lopes AH, Becerra A, Fonseca MM, Maganin A, Saraiva A, et al. Molecular mechanisms of zymosan-induced inflammasome activation in macrophages. Cell Signal. 2024;124:111418. 39304096 10.1016/j.cellsig.2024.111418

[189] Deerhake ME, Danzaki K, Inoue M, Cardakli ED, Nonaka T, Aggarwal N, et al. Dectin-1 limits autoimmune neuroinflammation and promotes myeloid cell-astrocyte crosstalk via Card9-independent expression of Oncostatin M. Immunity. 2021;54:484–498 e8. 33581044 10.1016/j.immuni.2021.01.004 PMC7956124

[190] Iliev ID, Funari VA, Taylor KD, Nguyen Q, Reyes CN, Strom SP, et al. Interactions between commensal fungi and the C-type lectin receptor Dectin-1 influence colitis. Science. 2012;336:1314–7. 22674328 10.1126/science.1221789 PMC3432565

[191] Taylor PR, Tsoni SV, Willment JA, Dennehy KM, Rosas M, Findon H, et al. Dectin-1 is required for beta-glucan recognition and control of fungal infection. Nat Immunol. 2007;8:31–8. 17159984 10.1038/ni1408 PMC1888731

[192] Turnbull C, Bones J, Stanley M, Medhavy A, Wang H, Lorenzo A, et al. DECTIN-1: A modifier protein in CTLA-4 haploinsufficiency. Sci Adv. 2023;9:eadi9566. 38055819 10.1126/sciadv.adi9566 PMC10699772

[193] Potere N, Garrad E, Kanthi Y, Di Nisio M, Kaplanski G, Bonaventura A, et al. NLRP3 inflammasome and interleukin-1 contributions to COVID-19-associated coagulopathy and immunothrombosis. Cardiovasc Res. 2023;119:2046–60. 37253117 10.1093/cvr/cvad084 PMC10893977

[194] Sefik E, Qu R, Junqueira C, Kaffe E, Mirza H, Zhao J, et al. Inflammasome activation in infected macrophages drives COVID-19 pathology. Nature. 2022;606:585–93. 35483404 10.1038/s41586-022-04802-1 PMC9288243

[195] Kitur K, Parker D, Nieto P, Ahn DS, Cohen TS, Chung S, et al. Toxin-induced necroptosis is a major mechanism of Staphylococcus aureus lung damage. PLoS Pathog. 2015;11:e1004820. 25880560 10.1371/journal.ppat.1004820 PMC4399879

[196] Liu C, Chi K, Yang M, Guo N. Staphylococcal Enterotoxin A Induces Intestinal Barrier Dysfunction and Activates NLRP3 Inflammasome via NF-kappaB/MAPK Signaling Pathways in Mice. Toxins. 2022;14:29. 35051006 10.3390/toxins14010029 PMC8779132

[197] Gu L, Zhu J, Nie Q, Xie B, Xue S, Zhang A, et al. NLRP3 promotes inflammatory signaling and IL-1beta cleavage in acute lung injury caused by cell wall extract of Lactobacillus casei. Commun Biol. 2025;8:20. 39774843 10.1038/s42003-025-07462-9 PMC11706994

[198] Tran VTA, Zhu X, Jamsranjav A, Lee LP, Cho H. Escherichia Coli K1-colibactin meningitis induces microglial NLRP3/IL-18 exacerbating H3K4me3-synucleinopathy in human inflammatory gut-brain axis. Commun Biol. 2025;8:382. 40050667 10.1038/s42003-025-07787-5 PMC11885818

[199] Yadavalli CS, Upparahalli Venkateshaiah S, Kumar S, Kandikattu HK, Oruganti L, Kathera CS, et al. Allergen-induced NLRP3/caspase1/IL-18 signaling initiate eosinophilic esophagitis and respective inhibitors protect disease pathogenesis. Commun Biol. 2023;6:763. 37524769 10.1038/s42003-023-05130-4 PMC10390481

[200] Galloway NL, Doitsh G, Monroe KM, Yang Z, Muñoz-Arias I, Levy DN, et al. Cell-to-Cell Transmission of HIV-1 Is Required to Trigger Pyroptotic Death of Lymphoid-Tissue-Derived CD4 T Cells. Cell Rep. 2015;12:1555–63. 26321639 10.1016/j.celrep.2015.08.011 PMC4565731

[201] Neven B, Callebaut I, Prieur AM, Feldmann J, Bodemer C, Lepore L, et al. Molecular basis of the spectral expression of CIAS1 mutations associated with phagocytic cell-mediated autoinflammatory disorders CINCA/NOMID, MWS, and FCU. Blood. 2004;103:2809–15. 14630794 10.1182/blood-2003-07-2531

[202] Feng S, Wierzbowski MC, Hrovat-Schaale K, Dumortier A, Zhang Y, Zyulina M, et al. Mechanisms of NLRP3 activation and inhibition elucidated by functional analysis of disease-associated variants. Nat Immunol. 2025;26:511–23. 39930093 10.1038/s41590-025-02088-9 PMC11876074

[203] Mangan MSJ, Olhava EJ, Roush WR, Seidel HM, Glick GD, Latz E. Targeting the NLRP3 inflammasome in inflammatory diseases. Nat Rev Drug Discov. 2018;17:588–606. 30026524 10.1038/nrd.2018.97

[204] Ising C, Venegas C, Zhang S, Scheiblich H, Schmidt SV, Vieira-Saecker A, et al. NLRP3 inflammasome activation drives tau pathology. Nature. 2019;575:669–73. 31748742 10.1038/s41586-019-1769-z PMC7324015

[205] Fan Z, Pan YT, Zhang ZY, Yang H, Yu SY, Zheng Y, et al. Systemic activation of NLRP3 inflammasome and plasma alpha-synuclein levels are correlated with motor severity and progression in Parkinson’s disease. J Neuroinflammation. 2020;17:11. 31915018 10.1186/s12974-019-1670-6 PMC6950934

[206] Allen IC, TeKippe EM, Woodford RM, Uronis JM, Holl EK, Rogers AB, et al. The NLRP3 inflammasome functions as a negative regulator of tumorigenesis during colitis-associated cancer. J Exp Med. 2010;207:1045–56. 20385749 10.1084/jem.20100050 PMC2867287

[207] Daley D, Mani VR, Mohan N, Akkad N, Pandian G, Savadkar S, et al. NLRP3 signaling drives macrophage-induced adaptive immune suppression in pancreatic carcinoma. J Exp Med. 2017;214:1711–24. 28442553 10.1084/jem.20161707 PMC5461004

[208] Ershaid N, Sharon Y, Doron H, Raz Y, Shani O, Cohen N, et al. NLRP3 inflammasome in fibroblasts links tissue damage with inflammation in breast cancer progression and metastasis. Nat Commun. 2019;10:4375. 31558756 10.1038/s41467-019-12370-8 PMC6763472

[209] Pandey A, Shen C, Man SM. Inflammasomes in Colitis and Colorectal Cancer: Mechanism of Action and Therapies. Yale J Biol Med. 2019;92:481–98. 31543710 PMC6747943

[210] Sharma BR, Kanneganti TD. NLRP3 inflammasome in cancer and metabolic diseases. Nat Immunol. 2021;22:550–9. 33707781 10.1038/s41590-021-00886-5 PMC8132572

[211] Karki R, Man SM, Kanneganti TD. Inflammasomes and Cancer. Cancer Immunol Res. 2017;5:94–99. 28093447 10.1158/2326-6066.CIR-16-0269 PMC5593081

[212] Man SM. Inflammasomes in the gastrointestinal tract: infection, cancer and gut microbiota homeostasis. Nat Rev Gastroenterol Hepatol. 2018;15:721–37. 30185915 10.1038/s41575-018-0054-1 PMC7097092

[213] Zaki MH, Boyd KL, Vogel P, Kastan MB, Lamkanfi M, Kanneganti TD. The NLRP3 inflammasome protects against loss of epithelial integrity and mortality during experimental colitis. Immunity. 2010;32:379–91. 20303296 10.1016/j.immuni.2010.03.003 PMC2982187

[214] Zaki MH, Vogel P, Body-Malapel M, Lamkanfi M, Kanneganti TD. IL-18 production downstream of the Nlrp3 inflammasome confers protection against colorectal tumor formation. J Immunol. 2010;185:4912–20. 20855874 10.4049/jimmunol.1002046 PMC3104023

[215] Man SM, Kaakoush NO, Mitchell HM. The role of bacteria and pattern-recognition receptors in Crohn’s disease. Nat Rev Gastroenterol Hepatol. 2011;8:152–68. 21304476 10.1038/nrgastro.2011.3

[216] Henao-Mejia J, Elinav E, Jin C, Hao L, Mehal WZ, Strowig T, et al. Inflammasome-mediated dysbiosis regulates progression of NAFLD and obesity. Nature. 2012;482:179–85. 22297845 10.1038/nature10809 PMC3276682

[217] Yu S, Pei S, Zhang M, Gao S, Chen J, Duan L, et al. PKM2-mediated STAT3 phosphorylation promotes acute liver failure by regulating NLRP3-dependent pyroptosis. Commun Biol. 2024;7:1694. 39722076 10.1038/s42003-024-07227-w PMC11669718

[218] Kim HY, Choi YJ, Kim SK, Kim H, Jun DW, Yoon K, et al. Auranofin prevents liver fibrosis by system Xc-mediated inhibition of NLRP3 inflammasome. Commun Biol. 2021;4:824. 34193972 10.1038/s42003-021-02345-1 PMC8245406

[219] Mridha AR, Wree A, Robertson A, Yeh MM, Johnson CD, Van Rooyen DM, et al. NLRP3 inflammasome blockade reduces liver inflammation and fibrosis in experimental NASH in mice. J Hepatol. 2017;66:1037–46. 28167322 10.1016/j.jhep.2017.01.022 PMC6536116

[220] Vande Walle L, Van Opdenbosch N, Jacques P, Fossoul A, Verheugen E, Vogel P, et al. Negative regulation of the NLRP3 inflammasome by A20 protects against arthritis. Nature. 2014;512:69–73. 25043000 10.1038/nature13322 PMC4126806

[221] Masters SL, Dunne A, Subramanian SL, Hull RL, Tannahill GM, Sharp FA, et al. Activation of the NLRP3 inflammasome by islet amyloid polypeptide provides a mechanism for enhanced IL-1beta in type 2 diabetes. Nat Immunol. 2010;11:897–904. 20835230 10.1038/ni.1935 PMC3103663

[222] Theivanthiran B, Evans KS, DeVito NC, Plebanek M, Sturdivant M, Wachsmuth LP, et al. A tumor-intrinsic PD-L1/NLRP3 inflammasome signaling pathway drives resistance to anti-PD-1 immunotherapy. J Clin Invest. 2020;130:2570–86. 32017708 10.1172/JCI133055 PMC7190922

[223] Wang S, Miura M, Jung YK, Zhu H, Li E, Yuan J. Murine caspase-11, an ICE-interacting protease, is essential for the activation of ICE. Cell. 1998;92:501–9. 9491891 10.1016/s0092-8674(00)80943-5

[224] Wang S, Miura M, Jung YK, Zhu H, Gagliardini V, Shi L, et al. Identification and characterization of Ich-3, a member of the interleukin-1beta converting enzyme (ICE)/Ced-3 family and an upstream regulator of ICE. J Biol Chem. 1996;271:20580–7. 8702803 10.1074/jbc.271.34.20580

[225] Ruhl S, Broz P. Caspase-11 activates a canonical NLRP3 inflammasome by promoting K(+) efflux. Eur J Immunol. 2015;45:2927–36. 26173909 10.1002/eji.201545772

[226] Baker PJ, Boucher D, Bierschenk D, Tebartz C, Whitney PG, D'Silva DB, et al. NLRP3 inflammasome activation downstream of cytoplasmic LPS recognition by both caspase-4 and caspase-5. Eur J Immunol. 2015;45:2918–26. 26173988 10.1002/eji.201545655

[227] Schmid-Burgk JL, Gaidt MM, Schmidt T, Ebert TS, Bartok E, Hornung V. Caspase-4 mediates noncanonical activation of the NLRP3 inflammasome in human myeloid cells. Eur J Immunol. 2015;45:2911–7. 26174085 10.1002/eji.201545523

[228] Kajiwara Y, Schiff T, Voloudakis G, Gama Sosa MA, Elder G, Bozdagi O, et al. A critical role for human caspase-4 in endotoxin sensitivity. J Immunol. 2014;193:335–43. 24879791 10.4049/jimmunol.1303424 PMC4066208

[229] Exconde PM, Hernandez-Chavez C, Bourne CM, Richards RM, Bray MB, Lopez JL, et al. The tetrapeptide sequence of IL-18 and IL-1beta regulates their recruitment and activation by inflammatory caspases. Cell Rep. 2023;42:113581. 38103201 10.1016/j.celrep.2023.113581 PMC11158830

[230] Shi X, Sun Q, Hou Y, Zeng H, Cao Y, Dong M, et al. Recognition and maturation of IL-18 by caspase-4 noncanonical inflammasome. Nature. 2023;624:442–50. 37993714 10.1038/s41586-023-06742-w

[231] Devant P, Dong Y, Mintseris J, Ma W, Gygi SP, Wu H, et al. Structural insights into cytokine cleavage by inflammatory caspase-4. Nature. 2023;624:451–9. 37993712 10.1038/s41586-023-06751-9 PMC10807405

[232] Bruce JK, Li LY, Tang Y, Forster E, Winsor NJ, Bi PY, et al. Gasdermin-D pores induce an inactivating caspase-4 cleavage that limits IL-18 production in the intestinal epithelium. Commun Biol. 2025;8:737. 40355718 10.1038/s42003-025-08183-9 PMC12069520

[233] Caroff M, Karibian D, Cavaillon JM, Haeffner-Cavaillon N. Structural and functional analyses of bacterial lipopolysaccharides. Microbes Infect. 2002;4:915–26. 12106784 10.1016/s1286-4579(02)01612-x

[234] Hagar JA, Powell DA, Aachoui Y, Ernst RK, Miao EA. Cytoplasmic LPS activates caspase-11: implications in TLR4-independent endotoxic shock. Science. 2013;341:1250–3. 24031018 10.1126/science.1240988 PMC3931427

[235] Liu M, Zhou K, Xu Z, Ma H, Cao X, Yin X, et al. Crystal structure of caspase-11 CARD provides insights into caspase-11 activation. Cell Discov. 2020;6:70. 33083005 10.1038/s41421-020-00201-w PMC7552397

[236] Ross C, Chan AH, Von Pein J, Boucher D, Schroder K. Dimerization and autoprocessing induce caspase-11 protease activation within the noncanonical inflammasome. Life Sci Alliance. 2018;1:e201800237. 30564782 10.26508/lsa.201800237 PMC6284101

[237] Lee BL, Stowe IB, Gupta A, Kornfeld OS, Roose-Girma M, Anderson K, et al. Caspase-11 autoproteolysis is crucial for noncanonical inflammasome activation. J Exp Med. 2018;215:2279–88. 30135078 10.1084/jem.20180589 PMC6122968

[238] Zhu F, Ma J, Li W, Liu Q, Qin X, Qian Y, et al. The orphan receptor Nur77 binds cytoplasmic LPS to activate the noncanonical NLRP3 inflammasome. Immunity. 2023;56:753–767.e8. 37001519 10.1016/j.immuni.2023.03.003

[239] Vasudevan SO, Russo AJ, Kumari P, Vanaja SK, Rathinam VA. A TLR4-independent critical role for CD14 in intracellular LPS sensing. Cell Rep. 2022;39:110755. 35508125 10.1016/j.celrep.2022.110755 PMC9376664

[240] Faucheu C, Diu A, Chan AW, Blanchet AM, Miossec C, Hervé F, et al. A novel human protease similar to the interleukin-1 beta converting enzyme induces apoptosis in transfected cells. EMBO J. 1995;14:1914–22. 7743998 10.1002/j.1460-2075.1995.tb07183.x PMC398290

[241] Kamens J, Paskind M, Hugunin M, Talanian RV, Allen H, Banach D, et al. Identification and characterization of ICH-2, a novel member of the interleukin-1 beta-converting enzyme family of cysteine proteases. J Biol Chem. 1995;270:15250–6. 7797510 10.1074/jbc.270.25.15250

[242] Munday NA, Vaillancourt JP, Ali A, Casano FJ, Miller DK, Molineaux SM, et al. Molecular cloning and pro-apoptotic activity of ICErelII and ICErelIII, members of the ICE/CED-3 family of cysteine proteases. J Biol Chem. 1995;270:15870–6. 7797592 10.1074/jbc.270.26.15870

[243] Faucheu C, Blanchet AM, Collard-Dutilleul V, Lalanne JL, Diu-Hercend A. Identification of a cysteine protease closely related to interleukin-1 beta-converting enzyme. Eur J Biochem. 1996;236:207–13. 8617266 10.1111/j.1432-1033.1996.t01-1-00207.x

[244] Lagrange B, Benaoudia S, Wallet P, Magnotti F, Provost A, Michal F, et al. Human caspase-4 detects tetra-acylated LPS and cytosolic Francisella and functions differently from murine caspase-11. Nat Commun. 2018;9:242. 29339744 10.1038/s41467-017-02682-y PMC5770465

[245] Enosi Tuipulotu D, Feng S, Pandey A, Zhao A, Ngo C, Mathur A, et al. Immunity against Moraxella catarrhalis requires guanylate-binding proteins and caspase-11-the NLRP3 inflammasome. EMBO J. 2023;42:e112558. 36762431 10.15252/embj.2022112558 PMC10015372

[246] Vanaja SK, Russo AJ, Behl B, Banerjee I, Yankova M, Deshmukh SD, et al. Bacterial Outer Membrane Vesicles Mediate Cytosolic Localization of LPS and Caspase-11 Activation. Cell. 2016;165:1106–19. 27156449 10.1016/j.cell.2016.04.015 PMC4874922

[247] Santos JC, Dick MS, Lagrange B, Degrandi D, Pfeffer K, Yamamoto M, et al. LPS targets host guanylate-binding proteins to the bacterial outer membrane for noncanonical inflammasome activation. EMBO J. 2018;37:e98089. 29459437 10.15252/embj.201798089 PMC5852652

[248] Duncan JA, Gao X, Huang MT, O'Connor BP, Thomas CE, Willingham SB, et al. Neisseria gonorrheae activates the proteinase cathepsin B to mediate the signaling activities of the NLRP3 and ASC-containing inflammasome. J Immunol. 2009;182:6460–9. 19414800 10.4049/jimmunol.0802696 PMC2722440

[249] Gurung P, Malireddi RK, Anand PK, Demon D, Vande Walle L, Liu Z, et al. Toll or interleukin-1 receptor (TIR) domain-containing adaptor inducing interferon-beta (TRIF)-mediated caspase-11 protease production integrates Toll-like receptor 4 (TLR4) protein- and Nlrp3 inflammasome-mediated host defense against enteropathogens. J. Biol Chem. 2012;287:34474–83. 10.1074/jbc.M112.401406 PMC3464552 22898816

[250] Broz P, Ruby T, Belhocine K, Bouley DM, Kayagaki N, Dixit VM, et al. Caspase-11 increases susceptibility to Salmonella infection in the absence of caspase-1. Nature. 2012;490:288–91. 22895188 10.1038/nature11419 PMC3470772

[251] Rathinam VA, Vanaja SK, Waggoner L, Sokolovska A, Becker C, Stuart LM, et al. TRIF licenses caspase-11-dependent NLRP3 inflammasome activation by gram-negative bacteria. Cell. 2012;150:606–19. 22819539 10.1016/j.cell.2012.07.007 PMC3660860

[252] Schauvliege R, Vanrobaeys J, Schotte P, Beyaert R. Caspase-11 gene expression in response to lipopolysaccharide and interferon-gamma requires nuclear factor-kappa B and signal transducer and activator of transcription (STAT) 1. J Biol Chem. 2002;277:41624–30. 12198138 10.1074/jbc.M207852200

[253] Lin XY, Choi MS, Porter AG. Expression analysis of the human caspase-1 subfamily reveals specific regulation of the CASP5 gene by lipopolysaccharide and interferon-gamma. J Biol Chem. 2000;275:39920–6. 10986288 10.1074/jbc.M007255200

[254] Man SM, Place DE, Kuriakose T, Kanneganti TD. Interferon-inducible guanylate-binding proteins at the interface of cell-autonomous immunity and inflammasome activation. J Leukoc Biol. 2017;101:143–50. 27418355 10.1189/jlb.4MR0516-223R PMC6608036

[255] Kirkby M, Enosi Tuipulotu D, Feng S, Lo Pilato J, Man SM. Guanylate-binding proteins: mechanisms of pattern recognition and antimicrobial functions. Trends Biochem Sci. 2023;48:883–93. 37567806 10.1016/j.tibs.2023.07.002

[256] Meunier E, Dick MS, Dreier RF, Schürmann N, Kenzelmann Broz D, Warming S, et al. Caspase-11 activation requires lysis of pathogen-containing vacuoles by IFN-induced GTPases. Nature. 2014;509:366–70. 24739961 10.1038/nature13157

[257] Man SM, Karki R, Sasai M, Place DE, Kesavardhana S, Temirov J, et al. IRGB10 Liberates Bacterial Ligands for Sensing by the AIM2 and Caspase-11-NLRP3 Inflammasomes. Cell. 2016;167:382–396 e17. 27693356 10.1016/j.cell.2016.09.012 PMC5074697

[258] Feeley EM, Pilla-Moffett DM, Zwack EE, Piro AS, Finethy R, Kolb JP, et al. Galectin-3 directs antimicrobial guanylate binding proteins to vacuoles furnished with bacterial secretion systems. Proc Natl Acad Sci USA. 2017;114:E1698–E1706. 28193861 10.1073/pnas.1615771114 PMC5338555

[259] Pilla DM, Hagar JA, Haldar AK, Mason AK, Degrandi D, Pfeffer K, et al. Guanylate binding proteins promote caspase-11-dependent pyroptosis in response to cytoplasmic LPS. Proc Natl Acad Sci USA. 2014;111:6046–51. 24715728 10.1073/pnas.1321700111 PMC4000848

[260] Kim BH, Shenoy AR, Kumar P, Das R, Tiwari S, MacMicking JD. A family of IFN-gamma-inducible 65-kD GTPases protects against bacterial infection. Science. 2011;332:717–21. 21551061 10.1126/science.1201711

[261] Fisch D, Bando H, Clough B, Hornung V, Yamamoto M, Shenoy AR, et al. Human GBP1 is a microbe-specific gatekeeper of macrophage apoptosis and pyroptosis. EMBO J. 2019;38:e100926. 31268602 10.15252/embj.2018100926 PMC6600649

[262] Fisch D, Clough B, Domart MC, Encheva V, Bando H, Snijders AP, et al. Human GBP1 Differentially Targets Salmonella and Toxoplasma to License Recognition of Microbial Ligands and Caspase-Mediated Death. Cell Rep. 2020;32:108008. 32783936 10.1016/j.celrep.2020.108008 PMC7435695

[263] Santos JC, Boucher D, Schneider LK, Demarco B, Dilucca M, Shkarina K, et al. Human GBP1 binds LPS to initiate assembly of a caspase-4 activating platform on cytosolic bacteria. Nat Commun. 2020;11:3276. 32581219 10.1038/s41467-020-16889-z PMC7314798

[264] Wandel MP, Kim BH, Park ES, Boyle KB, Nayak K, Lagrange B, et al. Guanylate-binding proteins convert cytosolic bacteria into caspase-4 signaling platforms. Nat Immunol. 2020;21:880–91. 32541830 10.1038/s41590-020-0697-2 PMC7381384

[265] Kuhm T, Taisne C, de Agrela Pinto C, Gross L, Giannopoulou EA, Huber ST, et al. Structural basis of antimicrobial membrane coat assembly by human GBP1. Nat Struct Mol Biol. 2025;32:172–84. 39394410 10.1038/s41594-024-01400-9 PMC11746146

[266] Zhu S, Bradfield CJ, Maminska A, Park ES, Kim BH, Kumar P, et al. Native architecture of a human GBP1 defense complex for cell-autonomous immunity to infection. Science. 2024;383:eabm9903. 38422126 10.1126/science.abm9903 PMC12091997

[267] Gaudet RG, Zhu S, Halder A, Kim BH, Bradfield CJ, Huang S, et al. A human apolipoprotein L with detergent-like activity kills intracellular pathogens. Science. 2021;373:eabf8113. 34437126 10.1126/science.abf8113 PMC8422858

[268] Kutsch M, Sistemich L, Lesser CF, Goldberg MB, Herrmann C, Coers J. Direct binding of polymeric GBP1 to LPS disrupts bacterial cell envelope functions. EMBO J. 2020;39:e104926. 32510692 10.15252/embj.2020104926 PMC7327485

[269] Li FJ, Starrs L, Mathur A, Enosi Tuipulotu D, Man SM, Burgio G. Interferon signaling and noncanonical inflammasome activation promote host protection against multidrug-resistant Acinetobacter baumannii. Commun Biol. 2024;7:1494. 39533032 10.1038/s42003-024-07204-3 PMC11557958

[270] Li FJ, Starrs L, Mathur A, Ishii H, Man SM, Burgio G. Differential activation of NLRP3 inflammasome by Acinetobacter baumannii strains. PLoS One. 2022;17:e0277019. 36318583 10.1371/journal.pone.0277019 PMC9624416

[271] Aachoui Y, Leaf IA, Hagar JA, Fontana MF, Campos CG, Zak DE, et al. Caspase-11 protects against bacteria that escape the vacuole. Science. 2013;339:975–8. 23348507 10.1126/science.1230751 PMC3697099

[272] Man SM, Karki R, Briard B, Burton A, Gingras S, Pelletier S, et al. Differential roles of caspase-1 and caspase-11 in infection and inflammation. Sci Rep. 2017;7:45126. 28345580 10.1038/srep45126 PMC5366862

[273] Li P, Allen H, Banerjee S, Franklin S, Herzog L, Johnston C, et al. Mice deficient in IL-1 beta-converting enzyme are defective in production of mature IL-1 beta and resistant to endotoxic shock. Cell. 1995;80:401–11. 7859282 10.1016/0092-8674(95)90490-5

[274] Cheng KT, Xiong S, Ye Z, Hong Z, Di A, Tsang KM, et al. Caspase-11-mediated endothelial pyroptosis underlies endotoxemia-induced lung injury. J Clin Invest. 2017;127:4124–35. 28990935 10.1172/JCI94495 PMC5663346

[275] Coutermarsh-Ott SL, Doran JT, Campbell C, Williams TM, Lindsay DS, Allen IC. Caspase-11 Modulates Inflammation and Attenuates Toxoplasma gondii Pathogenesis. Mediators Inflamm. 2016;2016:9848263. 27378827 10.1155/2016/9848263 PMC4917705

[276] Krause K, Franch Arroyo S, Ugolini M, Kueck T, Sullivan TJ, Gálvez EJC, et al. Streptococcus pyogenes EVs induce the alternative inflammasome via caspase-4/-5 in human monocytes. EMBO Rep. 2025 10.1038/s44319-025-00558-7. Online ahead of print. 10.1038/s44319-025-00558-7 PMC12508482 40925957

[277] Zanoni I, Tan Y, Di Gioia M, Broggi A, Ruan J, Shi J, et al. An endogenous caspase-11 ligand elicits interleukin-1 release from living dendritic cells. Science. 2016;352:1232–6. 27103670 10.1126/science.aaf3036 PMC5111085

[278] Chu LH, Indramohan M, Ratsimandresy RA, Gangopadhyay A, Morris EP, Monack DM, et al. The oxidized phospholipid oxPAPC protects from septic shock by targeting the noncanonical inflammasome in macrophages. Nat Commun. 2018;9:996. 29520027 10.1038/s41467-018-03409-3 PMC5843631

[279] Pizzuto M, Monteleone M, Burgener SS, Began J, Kurera M, Chia JR, et al. Cardiolipin inhibits the noncanonical inflammasome by preventing LPS binding to caspase-4/11. EMBO J. 2025;44:4419–42. 40670771 10.1038/s44318-025-00507-z PMC12361528

[280] Havira MS, Ta A, Kumari P, Wang C, Russo AJ, Ruan J, et al. Shiga toxin suppresses noncanonical inflammasome responses to cytosolic LPS. Sci Immunol. 2020;5:eabc0217. 33246946 10.1126/sciimmunol.abc0217 PMC7717664

[281] Yang C, Briones M, Chiou J, Lei L, Patton MJ, Ma L, et al. Chlamydia trachomatis Lipopolysaccharide Evades the Canonical and Noncanonical Inflammatory Pathways To Subvert Innate Immunity. mBio. 2019;10:e00595–19. 31015326 10.1128/mBio.00595-19 PMC6479002

[282] Pallett MA, Crepin VF, Serafini N, Habibzay M, Kotik O, Sanchez-Garrido J, et al. Bacterial virulence factor inhibits caspase-4/11 activation in intestinal epithelial cells. Mucosal Immunol. 2017;10:602–12. 27624779 10.1038/mi.2016.77 PMC5159625

[283] Blasche S, Mörtl M, Steuber H, Siszler G, Nisa S, Schwarz F, et al. The E. coli effector protein NleF is a caspase inhibitor. PLoS One. 2013;8:e58937. 23516580 10.1371/journal.pone.0058937 PMC3597564

[284] Kobayashi T, Ogawa M, Sanada T, Mimuro H, Kim M, Ashida H, et al. The Shigella OspC3 effector inhibits caspase-4, antagonizes inflammatory cell death, and promotes epithelial infection. Cell Host Microbe. 2013;13:570–83. 23684308 10.1016/j.chom.2013.04.012

[285] Demon D, Kuchmiy A, Fossoul A, Zhu Q, Kanneganti TD, Lamkanfi M. Caspase-11 is expressed in the colonic mucosa and protects against dextran sodium sulfate-induced colitis. Mucosal Immunol. 2014;7:1480–91. 24850431 10.1038/mi.2014.36 PMC4205216

[286] Oficjalska K, Raverdeau M, Aviello G, Wade SC, Hickey A, Sheehan KM, et al. Protective role for caspase-11 during acute experimental murine colitis. J Immunol. 2015;194:1252–60. 25548224 10.4049/jimmunol.1400501 PMC4298125

[287] Flood B, Manils J, Nulty C, Flis E, Kenealy S, Barber G, et al. Caspase-11 regulates the tumor suppressor function of STAT1 in a murine model of colitis-associated carcinogenesis. Oncogene. 2019;38:2658–74. 30538296 10.1038/s41388-018-0613-5 PMC6484510

[288] Marshall JC. Why have clinical trials in sepsis failed?. Trends Mol Med. 2014;20:195–203. 24581450 10.1016/j.molmed.2014.01.007

[289] Warren HS, Fitting C, Hoff E, Adib-Conquy M, Beasley-Topliffe L, Tesini B, et al. Resilience to bacterial infection: difference between species could be due to proteins in serum. J Infect Dis. 2010;201:223–32. 20001600 10.1086/649557 PMC2798011

[290] Coll RC, Robertson AA, Chae JJ, Higgins SC, Muñoz-Planillo R, Inserra MC, et al. A small-molecule inhibitor of the NLRP3 inflammasome for the treatment of inflammatory diseases. Nat Med. 2015;21:248–55. 25686105 10.1038/nm.3806 PMC4392179

[291] Hu JJ, Liu X, Xia S, Zhang Z, Zhang Y, Zhao J, et al. FDA-approved disulfiram inhibits pyroptosis by blocking gasdermin D pore formation. Nat Immunol. 2020;21:736–45. 32367036 10.1038/s41590-020-0669-6 PMC7316630

[292] Humphries F, Shmuel-Galia L, Ketelut-Carneiro N, Li S, Wang B, Nemmara VV, et al. Succination inactivates gasdermin D and blocks pyroptosis. Science. 2020;369:1633–7. 32820063 10.1126/science.abb9818 PMC8744141

[293] Elinav E, Strowig T, Kau AL, Henao-Mejia J, Thaiss CA, Booth CJ, et al. NLRP6 inflammasome regulates colonic microbial ecology and risk for colitis. Cell. 2011;145:745–57. 21565393 10.1016/j.cell.2011.04.022 PMC3140910

[294] Gremel G, Wanders A, Cedernaes J, Fagerberg L, Hallström B, Edlund K, et al. The human gastrointestinal tract-specific transcriptome and proteome as defined by RNA sequencing and antibody-based profiling. J Gastroenterol. 2015;50:46–57. 24789573 10.1007/s00535-014-0958-7

[295] Lech M, Avila-Ferrufino A, Skuginna V, Susanti HE, Anders HJ. Quantitative expression of RIG-like helicase, NOD-like receptor and inflammasome-related mRNAs in humans and mice. Int Immunol. 2010;22:717–28. 20584763 10.1093/intimm/dxq058

[296] Wei B, Billman ZP, Nozaki K, Goodridge HS, Miao EA. NLRP3, NLRP6, and NLRP12 are inflammasomes with distinct expression patterns. Front Immunol. 2024;15:1418290. 39076995 10.3389/fimmu.2024.1418290 PMC11284034

[297] Grenier JM, Wang L, Manji GA, Huang WJ, Al-Garawi A, Kelly R, et al. Functional screening of five PYPAF family members identifies PYPAF5 as a novel regulator of NF-kappaB and caspase-1. FEBS Lett. 2002;530:73–8. 12387869 10.1016/s0014-5793(02)03416-6

[298] Anand PK, Malireddi RK, Lukens JR, Vogel P, Bertin J, Lamkanfi M, et al. NLRP6 negatively regulates innate immunity and host defense against bacterial pathogens. Nature. 2012;488:389–93. 22763455 10.1038/nature11250 PMC3422416

[299] Xu D, Wu X, Peng L, Chen T, Huang Q, Wang Y, et al. The Critical Role of NLRP6 Inflammasome in Streptococcus pneumoniae Infection In Vitro and In Vivo. Int J Mol Sci. 2021;22:3876. 33918100 10.3390/ijms22083876 PMC8069100

[300] Ghimire L, Paudel S, Jin L, Baral P, Cai S, Jeyaseelan S. NLRP6 negatively regulates pulmonary host defense in gram-positive bacterial infection through modulating neutrophil recruitment and function. PLoS Pathog. 2018;14:e1007308. 30248149 10.1371/journal.ppat.1007308 PMC6171945

[301] Hara H, Seregin SS, Yang D, Fukase K, Chamaillard M, Alnemri ES, et al. The NLRP6 Inflammasome Recognizes Lipoteichoic Acid and Regulates Gram-Positive Pathogen Infection. Cell. 2018;175:1651–1664 e14. 30392956 10.1016/j.cell.2018.09.047 PMC6294477

[302] Levy M, Thaiss CA, Zeevi D, Dohnalová L, Zilberman-Schapira G, Mahdi JA, et al. Microbiota-Modulated Metabolites Shape the Intestinal Microenvironment by Regulating NLRP6 Inflammasome Signaling. Cell. 2015;163:1428–43. 26638072 10.1016/j.cell.2015.10.048 PMC5665753

[303] Leng F, Yin H, Qin S, Zhang K, Guan Y, Fang R, et al. NLRP6 self-assembles into a linear molecular platform following LPS binding and ATP stimulation. Sci Rep. 2020;10:198. 31932628 10.1038/s41598-019-57043-0 PMC6957519

[304] Lu WL, Zhang L, Song DZ, Yi XW, Xu WZ, Ye L, et al. NLRP6 suppresses the inflammatory response of human periodontal ligament cells by inhibiting NF-kappaB and ERK signaling pathways. Int Endod J. 2019;52:999–1009. 30712265 10.1111/iej.13091

[305] Shen C, Li R, Negro R, Cheng J, Vora SM, Fu TM, et al. Phase separation drives RNA virus-induced activation of the NLRP6 inflammasome. Cell. 2021;184:5759–5774 e20. 34678144 10.1016/j.cell.2021.09.032 PMC8643277

[306] Mukherjee S, Kumar R, Tsakem Lenou E, Basrur V, Kontoyiannis DL, Ioakeimidis F, et al. Deubiquitination of NLRP6 inflammasome by Cyld critically regulates intestinal inflammation. Nat Immunol. 2020;21:626–35. 32424362 10.1038/s41590-020-0681-x PMC7881443

[307] Wlodarska M, Thaiss CA, Nowarski R, Henao-Mejia J, Zhang JP, Brown EM, et al. NLRP6 inflammasome orchestrates the colonic host–microbe interface by regulating goblet cell mucus secretion. Cell. 2014;156:1045–59. 24581500 10.1016/j.cell.2014.01.026 PMC4017640

[308] Seregin SS, Golovchenko N, Schaf B, Chen J, Pudlo NA, Mitchell J, et al. NLRP6 Protects Il10(-/-) Mice from Colitis by Limiting Colonization of Akkermansia muciniphila. Cell Rep. 2017;19:733–45. 28445725 10.1016/j.celrep.2017.03.080 PMC5528001

[309] Lemire P, Robertson SJ, Maughan H, Tattoli I, Streutker CJ, Platnich JM, et al. The NLR Protein NLRP6 Does Not Impact Gut Microbiota Composition. Cell Rep. 2017;21:3653–61. 29281815 10.1016/j.celrep.2017.12.026

[310] Mamantopoulos M, Ronchi F, Van Hauwermeiren F, Vieira-Silva S, Yilmaz B, Martens L, et al. Nlrp6- and ASC-Dependent Inflammasomes Do Not Shape the Commensal Gut Microbiota Composition. Immunity. 2017;47:339–348 e4. 28801232 10.1016/j.immuni.2017.07.011

[311] Bai Y, Li S. Long noncoding RNA OIP5-AS1 aggravates cell proliferation, migration in gastric cancer by epigenetically silencing NLRP6 expression by binding EZH2. J Cell Biochem. 2020;121:353–62. 31219209 10.1002/jcb.29183

[312] Ranson N, Veldhuis M, Mitchell B, Fanning S, Cook AL, Kunde D, et al. Nod-Like Receptor Pyrin-Containing Protein 6 (NLRP6) Is Upregulated in Ileal Crohn’s Disease and Differentially Expressed in Goblet Cells. Cell Mol Gastroenterol Hepatol. 2018;6:110–112.e8. 29928676 10.1016/j.jcmgh.2018.03.001 PMC6007817

[313] Toubai T, Fujiwara H, Rossi C, Riwes M, Tamaki H, Zajac C, et al. Host NLRP6 exacerbates graft-versus-host disease independent of gut microbial composition. Nat Microbiol. 2019;4:800–12. 30858572 10.1038/s41564-019-0373-1 PMC6689241

[314] Wang X, Wu X, Wang Q, Zhang Y, Wang C, Chen J. NLRP6 suppresses gastric cancer growth via GRP78 ubiquitination. Exp Cell Res. 2020;395:112177. 32682010 10.1016/j.yexcr.2020.112177

[315] Angosto-Bazarra D, Molina-Lopez C, Pelegrin P. Physiological and pathophysiological functions of NLRP6: pro- and anti-inflammatory roles. Commun Biol. 2022;5:524. 35650327 10.1038/s42003-022-03491-w PMC9160023

[316] Tomuschat C, Virbel CR, O'Donnell AM, Puri P. Reduced expression of the NLRP6 inflammasome in the colon of patients with Hirschsprung’s disease. J Pediatr Surg. 2019;54:1573–7. 30262203 10.1016/j.jpedsurg.2018.08.059

[317] Ji X, Li L, Lu P, Li X, Tian D, Liu M. NLRP6 exerts a protective role via NF-kB with involvement of CCL20 in a mouse model of alcoholic hepatitis. Biochem Biophys Res Commun. 2020;528:485–92. 32507279 10.1016/j.bbrc.2020.05.171

[318] Lin Y, Luo Z. NLRP6 facilitates the interaction between TAB2/3 and TRIM38 in rheumatoid arthritis fibroblast-like synoviocytes. FEBS Lett. 2017;591:1141–9. 28295271 10.1002/1873-3468.12622

[319] Wang P, Zhu S, Yang L, Cui S, Pan W, Jackson R, et al. Nlrp6 regulates intestinal antiviral innate immunity. Science. 2015;350:826–30. 26494172 10.1126/science.aab3145 PMC4927078

[320] Ydens E, Demon D, Lornet G, De Winter V, Timmerman V, Lamkanfi M, et al. Nlrp6 promotes recovery after peripheral nerve injury independently of inflammasomes. J Neuroinflammation. 2015;12:143. 26253422 10.1186/s12974-015-0367-8 PMC4528710

[321] Zhi F, Li B, Zhang C, Xia F, Wang R, Xie W, et al. NLRP6 potentiates PI3K/AKT signaling by promoting autophagic degradation of p85alpha to drive tumorigenesis. Nat Commun. 2023;14:6069. 37770465 10.1038/s41467-023-41739-z PMC10539329

[322] Zhang P, Dixon M, Zucchelli M, Hambiliki F, Levkov L, Hovatta O, et al. Expression analysis of the NLRP gene family suggests a role in human preimplantation development. PLoS One. 2008;3:e2755. 18648497 10.1371/journal.pone.0002755 PMC2447171

[323] Van Gorp H, Kuchmiy A, Van Hauwermeiren F, Lamkanfi M. NOD-like receptors interfacing the immune and reproductive systems. FEBS J. 2014;281:4568–82. 25154302 10.1111/febs.13014

[324] Kinoshita T, Wang Y, Hasegawa M, Imamura R, Suda T. PYPAF3, a PYRIN-containing APAF-1-like protein, is a feedback regulator of caspase-1-dependent interleukin-1beta secretion. J Biol Chem. 2005;280:21720–5. 15817483 10.1074/jbc.M410057200

[325] Tian X, Pascal G, Monget P. Evolution and functional divergence of NLRP genes in mammalian reproductive systems. BMC Evol Biol. 2009;9:202. 19682372 10.1186/1471-2148-9-202 PMC2735741

[326] Khare S, Dorfleutner A, Bryan NB, Yun C, Radian AD, de Almeida L, et al. An NLRP7-containing inflammasome mediates recognition of microbial lipopeptides in human macrophages. Immunity. 2012;36:464–76. 22361007 10.1016/j.immuni.2012.02.001 PMC3315380

[327] Zhou Y, Shah SZ, Yang L, Zhang Z, Zhou X, Zhao D. Virulent Mycobacterium bovis Beijing Strain Activates the NLRP7 Inflammasome in THP-1 Macrophages. PLoS One. 2016;11:e0152853. 27043315 10.1371/journal.pone.0152853 PMC4820140

[328] Lavergne M, Belville C, Choltus H, Gross C, Minet-Quinard R, Gallot D, et al. Human Amnion Epithelial Cells (AECs) Respond to the FSL-1 Lipopeptide by Engaging the NLRP7 Inflammasome. Front Immunol. 2020;11:1645. 32849565 10.3389/fimmu.2020.01645 PMC7426397

[329] Radian AD, Khare S, Chu LH, Dorfleutner A, Stehlik C. ATP binding by NLRP7 is required for inflammasome activation in response to bacterial lipopeptides. Mol Immunol. 2015;67:294–302. 26143398 10.1016/j.molimm.2015.06.013 PMC4565763

[330] Bednash JS, Weathington N, Londino J, Rojas M, Gulick DL, Fort R, et al. Targeting the deubiquitinase STAMBP inhibits NALP7 inflammasome activity. Nat Commun. 2017;8:15203. 28492230 10.1038/ncomms15203 PMC5437278

[331] Radian AD, de Almeida L, Dorfleutner A, Stehlik C. NLRP7 and related inflammasome activating pattern recognition receptors and their function in host defense and disease. Microbes Infect. 2013;15:630–9. 23618810 10.1016/j.micinf.2013.04.001 PMC3722249

[332] Carriere J, Dorfleutner A, Stehlik C. NLRP7: From inflammasome regulation to human disease. Immunology. 2021;163:363–76. 34021586 10.1111/imm.13372 PMC8274175

[333] Chen Z, Jiang L, Su M, Zeng Q, Luo P, Chu L. NLRP7 maintains the genomic stability during early human embryogenesis by mediating alternative splicing. Commun Biol. 2025;8:125. 39865169 10.1038/s42003-025-07571-5 PMC11770114

[334] Wang CM, Dixon PH, Decordova S, Hodges MD, Sebire NJ, Ozalp S, et al. Identification of 13 novel NLRP7 mutations in 20 families with recurrent hydatidiform mole; missense mutations cluster in the leucine-rich region. J Med Genet. 2009;46:569–75. 19246479 10.1136/jmg.2008.064196

[335] Silber M, Dekel N, Heusler I, Biron-Shental T, Amiel A, Kidron D, et al. Inflammasome activation in preeclampsia and intrauterine growth restriction. Am J Reprod Immunol. 2022;88:e13598. 35976163 10.1111/aji.13598

[336] Fallahi J, Razban V, Momtahan M, Akbarzadeh-Jahromi M, Namavar-Jahromi B, Anvar Z, et al. A Novel Mutation in NLRP7 Related to Recurrent Hydatidiform Mole and Reproductive Failure. Int J Fertil Steril. 2019;13:135–8. 31037924 10.22074/ijfs.2019.5657 PMC6500085

[337] Jing X, Yun Y, Ji X, Yang E, Li P. Pyroptosis and Inflammasome-Related Genes-NLRP3, NLRC4 and NLRP7 Polymorphisms Were Associated with Risk of Lung Cancer. Pharmgenomics Pers Med. 2023;16:795–804. 37650010 10.2147/PGPM.S424326 PMC10464886

[338] Onoufriadis A, Stone K, Katsiamides A, Amar A, Omar Y, de Lange KM, et al. Exome Sequencing and Genotyping Identify a Rare Variant in NLRP7 Gene Associated With Ulcerative Colitis. J Crohns Colitis. 2018;12:321–6. 29211899 10.1093/ecco-jcc/jjx157 PMC6290881

[339] Reynaud D, Alfaidy N, Collet C, Lemaitre N, Sergent F, Miege C, et al. NLRP7 Enhances Choriocarcinoma Cell Survival and Camouflage in an Inflammasome Independent Pathway. Cells. 2023;12:857. 36980199 10.3390/cells12060857 PMC10099745

[340] Amoushahi M, Steffensen LL, Galieva A, Agger J, Heuck A, Siupka P, et al. Maternally contributed Nlrp9b expressed in human and mouse ovarian follicles contributes to early murine preimplantation development. J Assist Reprod Genet. 2020;37:1355–65. 32399794 10.1007/s10815-020-01767-w PMC7311623

[341] Dalbiès-Tran R, Papillier P, Pennetier S, Uzbekova S, Monget P. Bovine mater-like NALP9 is an oocyte marker gene. Mol Reprod Dev. 2005;71:414–21. 15892040 10.1002/mrd.20298

[342] Peng H, Lin X, Liu F, Wang C, Zhang W. NLRP9B protein is dispensable for oocyte maturation and early embryonic development in the mouse. J Reprod Dev. 2015;61:559–64. 26411641 10.1262/jrd.2015-050 PMC4685222

[343] Ponsuksili S, Brunner RM, Goldammer T, Kühn C, Walz C, Chomdej S, et al. Bovine NALP5, NALP8, and NALP9 genes: assignment to a QTL region and the expression in adult tissues, oocytes, and preimplantation embryos. Biol Reprod. 2006;74:577–84. 16339045 10.1095/biolreprod.105.045096

[344] Ferrao R, Wu H. Helical assembly in the death domain (DD) superfamily. Curr Opin Struct Biol. 2012;22:241–7. 22429337 10.1016/j.sbi.2012.02.006 PMC3320699

[345] Ha HJ, Park HH. Crystal structure of the human NLRP9 pyrin domain reveals a bent N-terminal loop that may regulate inflammasome assembly. FEBS Lett. 2020;594:2396–405. 32542766 10.1002/1873-3468.13866

[346] Marleaux M, Anand K, Latz E, Geyer M. Crystal structure of the human NLRP9 pyrin domain suggests a distinct mode of inflammasome assembly. FEBS Lett. 2020;594:2383–95. 32542665 10.1002/1873-3468.13865

[347] Zhu S, Ding S, Wang P, Wei Z, Pan W, Palm NW, et al. Nlrp9b inflammasome restricts rotavirus infection in intestinal epithelial cells. Nature. 2017;546:667–70. 28636595 10.1038/nature22967 PMC5787375

[348] Chen YG, Hur S. Cellular origins of dsRNA, their recognition and consequences. Nat Rev Mol Cell Biol. 2022;23:286–301. 34815573 10.1038/s41580-021-00430-1 PMC8969093

[349] Qin G, Yu X, Zhao Y, Li X, Yu B, Peng H, et al. NLRP9 involved in antiviral innate immunity by binding VIM in IPEC-J2 cells. Dev Comp Immunol. 2023;147:104895. 37473827 10.1016/j.dci.2023.104895

[350] Satoh J, Yamamura T, Arima K. The 14-3-3 protein epsilon isoform expressed in reactive astrocytes in demyelinating lesions of multiple sclerosis binds to vimentin and glial fibrillary acidic protein in cultured human astrocytes. Am J Pathol. 2004;165:577–92. 15277231 10.1016/s0002-9440(10)63322-6 PMC1618573

[351] Gil-Varea E, Urcelay E, Vilariño-Güell C, Costa C, Midaglia L, Matesanz F, et al. Exome sequencing study in patients with multiple sclerosis reveals variants associated with disease course. J Neuroinflammation. 2018;15:265. 30217166 10.1186/s12974-018-1307-1 PMC6138928

[352] Fernández MV, Budde J, Del-Aguila JL, Ibañez L, Deming Y, Harari O, et al. Evaluation of Gene-Based Family-Based Methods to Detect Novel Genes Associated With Familial Late Onset Alzheimer Disease. Front Neurosci. 2018;12:209. 29670507 10.3389/fnins.2018.00209 PMC5893779

[353] Moon SW, Son HJ, Mo HY, Yoo NJ, Lee SH. Somatic Mutation of NLRP Genes in Gastric and Colonic Cancers. Pathol Oncol Res. 2021;27:607385. 34257569 10.3389/pore.2021.607385 PMC8262223

[354] Zheng D, Mohapatra G, Kern L, He Y, Shmueli MD, Valdés-Mas R, et al. Epithelial Nlrp10 inflammasome mediates protection against intestinal autoinflammation. Nat Immunol. 2023;24:585–94. 36941399 10.1038/s41590-023-01450-z

[355] Próchnicki T, Vasconcelos MB, Robinson KS, Mangan M, De Graaf D, Shkarina K, et al. Mitochondrial damage activates the NLRP10 inflammasome. Nat Immunol. 2023;24:595–603. 36941400 10.1038/s41590-023-01451-y

[356] Wang Y, Hasegawa M, Imamura R, Kinoshita T, Kondo C, Konaka K, et al. PYNOD, a novel Apaf-1/CED4-like protein is an inhibitor of ASC and caspase-1. Int Immunol. 2004;16:777–86. 15096476 10.1093/intimm/dxh081

[357] Imamura R, Wang Y, Kinoshita T, Suzuki M, Noda T, Sagara J, et al. Anti-Inflammatory Activity of PYNOD and Its Mechanism in Humans and Mice. J Immunol. 2010;184:5874–84. 20393137 10.4049/jimmunol.0900779

[358] Lautz K, Damm A, Menning M, Wenger J, Adam AC, Zigrino P, et al. NLRP10 enhances Shigella-induced pro-inflammatory responses. Cell Microbiol. 2012;14:1568–83. 22672233 10.1111/j.1462-5822.2012.01822.x

[359] Damm A, Giebeler N, Zamek J, Zigrino P, Kufer TA. Epidermal NLRP10 contributes to contact hypersensitivity responses in mice. Eur J Immunol. 2016;46:1959–69. 27221772 10.1002/eji.201646401

[360] Vacca M, Böhme J, Zambetti LP, Khameneh HJ, Paleja BS, Laudisi F, et al. NLRP10 enhances CD4+ T-cell-mediated IFNγ response via regulation of dendritic cell-derived IL-12 release. Front Immunol. 2017;8:1462. 29163529 10.3389/fimmu.2017.01462 PMC5673625

[361] Joly S, Eisenbarth SC, Olivier AK, Williams A, Kaplan DH, Cassel SL, et al. Cutting edge: Nlrp10 is essential for protective antifungal adaptive immunity against Candida albicans. J Immunol. 2012;189:4713–7. 23071280 10.4049/jimmunol.1201715 PMC3548226

[362] Clay GM, Valadares DG, Graff JW, Ulland TK, Davis RE, Scorza BM, et al. An anti-inflammatory role for NLRP10 in murine cutaneous leishmaniasis. J Immunol. 2017;199:2823–33. 28931602 10.4049/jimmunol.1500832 PMC5679237

[363] Eisenbarth SC, Williams A, Colegio OR, Meng H, Strowig T, Rongvaux A, et al. NLRP10 is a NOD-like receptor essential to initiate adaptive immunity by dendritic cells. Nature. 2012;484:510–3. 22538615 10.1038/nature11012 PMC3340615

[364] Krishnaswamy JK, Singh A, Gowthaman U, Wu R, Gorrepati P, Sales Nascimento M, et al. Coincidental loss of DOCK8 function in NLRP10-deficient and C3H/HeJ mice results in defective dendritic cell migration. Proc Natl Acad Sci. 2015;112:3056–61. 25713392 10.1073/pnas.1501554112 PMC4364188

[365] Tanaka N, Koido M, Suzuki A, Otomo N, Suetsugu H, Kochi Y, et al. Eight novel susceptibility loci and putative causal variants in atopic dermatitis. J Allergy Clin Immunol. 2021;148:1293–306. 34116867 10.1016/j.jaci.2021.04.019

[366] Shami PJ, Kanai N, Wang LY, Vreeke TM, Parker CH. Identification and characterization of a novel gene that is upregulated in leukemia cells by nitric oxide. Br J Hematol. 2001;112:138–47. 10.1046/j.1365-2141.2001.02491.x 11167794

[367] Williams KL, Taxman DJ, Linhoff MW, Reed W, Ting JP. Cutting edge: Monarch-1: a pyrin/nucleotide-binding domain/leucine-rich repeat protein that controls classical and nonclassical MHC class I genes. J Immunol. 2003;170:5354–8. 12759408 10.4049/jimmunol.170.11.5354

[368] Lich JD, Williams KL, Moore CB, Arthur JC, Davis BK, Taxman DJ, et al. Monarch-1 suppresses noncanonical NF-kappaB activation and p52-dependent chemokine expression in monocytes. J Immunol. 2007;178:1256–60. 17237370 10.4049/jimmunol.178.3.1256

[369] Williams KL, Lich JD, Duncan JA, Reed W, Rallabhandi P, Moore C, et al. The CATERPILLER protein monarch-1 is an antagonist of toll-like receptor-, tumor necrosis factor alpha-, and Mycobacterium tuberculosis-induced pro-inflammatory signals. J Biol Chem. 2005;280:39914–24. 16203735 10.1074/jbc.M502820200 PMC4422647

[370] Zaki MH, Man SM, Vogel P, Lamkanfi M, Kanneganti TD. Salmonella exploits NLRP12-dependent innate immune signaling to suppress host defenses during infection. Proc Natl Acad Sci USA. 2014;111:385–90. 24347638 10.1073/pnas.1317643111 PMC3890849

[371] Ataide MA, Andrade WA, Zamboni DS, Wang D, Souza Mdo C, Franklin BS, et al. Malaria-induced NLRP12/NLRP3-dependent caspase-1 activation mediates inflammation and hypersensitivity to bacterial superinfection. PLoS Pathog. 2014;10:e1003885. 24453977 10.1371/journal.ppat.1003885 PMC3894209

[372] Vladimer GI, Weng D, Paquette SW, Vanaja SK, Rathinam VA, Aune MH, et al. The NLRP12 inflammasome recognizes Yersinia pestis. Immunity. 2012;37:96–107. 22840842 10.1016/j.immuni.2012.07.006 PMC3753114

[373] Sundaram B, Pandian N, Mall R, Wang Y, Sarkar R, Kim HJ, et al. NLRP12-PANoptosome activates PANoptosis and pathology in response to heme and PAMPs. Cell. 2023;186:2783–2801.e20. 37267949 10.1016/j.cell.2023.05.005 PMC10330523

[374] Coombs JR, Zamoshnikova A, Holley CL, Maddugoda MP, Teo D, Chauvin C, et al. NLRP12 interacts with NLRP3 to block the activation of the human NLRP3 inflammasome. Sci Signal. 2024;17:eabg8145. 38261657 10.1126/scisignal.abg8145

[375] Pinheiro AS, Eibl C, Ekman-Vural Z, Schwarzenbacher R, Peti W. The NLRP12 pyrin domain: structure, dynamics, and functional insights. J Mol Biol. 2011;413:790–803. 21978668 10.1016/j.jmb.2011.09.024 PMC3202057

[376] Park MY, Jang HD, Lee SY, Lee KJ, Kim E. Fas-associated factor-1 inhibits nuclear factor-kappaB (NF-kappaB) activity by interfering with nuclear translocation of the RelA (p65) subunit of NF-kappaB. J Biol Chem. 2004;279:2544–9. 14600157 10.1074/jbc.M304565200

[377] Sundaram B, Pandian N, Kim HJ, Abdelaal HM, Mall R, Indari O, et al. NLRC5 senses NAD(+) depletion, forming a PANoptosome and driving PANoptosis and inflammation. Cell. 2024;187:4061–4077.e17. 38878777 10.1016/j.cell.2024.05.034 PMC11283362

[378] Rossi MS, Fetherston JD, Létoffé S, Carniel E, Perry RD, Ghigo JM. Identification and characterization of the hemophore-dependent heme acquisition system of Yersinia pestis. Infect Immun. 2001;69:6707–17. 11598042 10.1128/IAI.69.11.6707-6717.2001 PMC100047

[379] Francis SE, Sullivan DJ Jr, Goldberg andDE. Hemoglobin metabolism in the malaria parasite Plasmodium falciparum. Annu Rev Microbiol. 1997;51:97–123. 9343345 10.1146/annurev.micro.51.1.97

[380] Poyet JL, Srinivasula SM, Tnani M, Razmara M, Fernandes-Alnemri T, Alnemri ES. Identification of Ipaf, a human caspase-1-activating protein related to Apaf-1. J Biol Chem. 2001;276:28309–13. 11390368 10.1074/jbc.C100250200

[381] Kay C, Wang R, Kirkby M, Man SM. Molecular mechanisms activating the NAIP-NLRC4 inflammasome: Implications in infectious disease, autoinflammation, and cancer. Immunol Rev. 2020;297:67–82. 32729154 10.1111/imr.12906

[382] Shen C, Pandey A, Enosi Tuipulotu D, Mathur A, Liu L, Yang H, et al. Inflammasome protein scaffolds the DNA damage complex during tumor development. Nat Immunol. 2024;25:2085–96. 39402152 10.1038/s41590-024-01988-6

[383] Mariathasan S, Newton K, Monack DM, Vucic D, French DM, Lee WP, et al. Differential activation of the inflammasome by caspase-1 adaptors ASC and Ipaf. Nature. 2004;430:213–8. 15190255 10.1038/nature02664

[384] Franchi L, Amer A, Body-Malapel M, Kanneganti TD, Ozören N, Jagirdar R, et al. Cytosolic flagellin requires Ipaf for activation of caspase-1 and interleukin 1beta in salmonella-infected macrophages. Nat Immunol. 2006;7:576–82. 16648852 10.1038/ni1346

[385] Miao EA, Alpuche-Aranda CM, Dors M, Clark AE, Bader MW, Miller SI, et al. Cytoplasmic flagellin activates caspase-1 and secretion of interleukin 1beta via Ipaf. Nat Immunol. 2006;7:569–75. 16648853 10.1038/ni1344

[386] Cerqueira DM, Pereira MS, Silva AL, Cunha LD, Zamboni DS. Caspase-1 but Not Caspase-11 Is Required for NLRC4-Mediated Pyroptosis and Restriction of Infection by Flagellated Legionella Species in Mouse Macrophages and In Vivo. J Immunol. 2015;195:2303–11. 26232428 10.4049/jimmunol.1501223

[387] Gonçalves AV, Margolis SR, Quirino G, Mascarenhas D, Rauch I, Nichols RD, et al. Gasdermin-D and Caspase-7 are the key Caspase-1/8 substrates downstream of the NAIP5/NLRC4 inflammasome required for restriction of Legionella pneumophila. PLoS Pathog. 2019;15:e1007886. 31251782 10.1371/journal.ppat.1007886 PMC6622555

[388] Pereira MS, Marques GG, Dellama JE, Zamboni DS. The Nlrc4 Inflammasome Contributes to Restriction of Pulmonary Infection by Flagellated Legionella spp. that Trigger Pyroptosis. Front Microbiol. 2011;2:33. 21687424 10.3389/fmicb.2011.00033 PMC3109297

[389] Pereira MS, Morgantetti GF, Massis LM, Horta CV, Hori JI, Zamboni DS. Activation of NLRC4 by flagellated bacteria triggers caspase-1-dependent and -independent responses to restrict Legionella pneumophila replication in macrophages and in vivo. J Immunol. 2011;187:6447–55. 22079982 10.4049/jimmunol.1003784

[390] Miao EA, Mao DP, Yudkovsky N, Bonneau R, Lorang CG, Warren SE, et al. Innate immune detection of the type III secretion apparatus through the NLRC4 inflammasome. Proc Natl Acad Sci USA. 2010;107:3076–80. 20133635 10.1073/pnas.0913087107 PMC2840275

[391] Endrizzi MG, Hadinoto V, Growney JD, Miller W, Dietrich WF. Genomic sequence analysis of the mouse Naip gene array. Genome Res. 2000;10:1095–102. 10958627 10.1101/gr.10.8.1095 PMC310933

[392] Diez E, Lee SH, Gauthier S, Yaraghi Z, Tremblay M, Vidal S, et al. Birc1e is the gene within the Lgn1 locus associated with resistance to Legionella pneumophila. Nat Genet. 2003;33:55–60. 12483212 10.1038/ng1065

[393] Wright EK, Goodart SA, Growney JD, Hadinoto V, Endrizzi MG, Long EM, et al. Naip5 affects host susceptibility to the intracellular pathogen Legionella pneumophila. Curr Biol. 2003;13:27–36. 12526741 10.1016/s0960-9822(02)01359-3

[394] Lightfield KL, Persson J, Brubaker SW, Witte CE, von Moltke J, Dunipace EA, et al. Critical function for Naip5 in inflammasome activation by a conserved carboxy-terminal domain of flagellin. Nat Immunol. 2008;9:1171–8. 18724372 10.1038/ni.1646 PMC2614210

[395] Molofsky AB, Byrne BG, Whitfield NN, Madigan CA, Fuse ET, Tateda K, et al. Cytosolic recognition of flagellin by mouse macrophages restricts Legionella pneumophila infection. J Exp Med. 2006;203:1093–104. 16606669 10.1084/jem.20051659 PMC1584282

[396] Ren T, Zamboni DS, Roy CR, Dietrich WF, Vance RE. Flagellin-deficient Legionella mutants evade caspase-1- and Naip5-mediated macrophage immunity. PLoS Pathog. 2006;2:e18. 16552444 10.1371/journal.ppat.0020018 PMC1401497

[397] Kofoed EM, Vance RE. Innate immune recognition of bacterial ligands by NAIPs determines inflammasome specificity. Nature. 2011;477:592–5. 21874021 10.1038/nature10394 PMC3184209

[398] Zhao Y, Yang J, Shi J, Gong YN, Lu Q, Xu H, et al. The NLRC4 inflammasome receptors for bacterial flagellin and type III secretion apparatus. Nature. 2011;477:596–600. 21918512 10.1038/nature10510

[399] Rayamajhi M, Zak DE, Chavarria-Smith J, Vance RE, Miao EA. Cutting edge: Mouse NAIP1 detects the type III secretion system needle protein. J Immunol. 2013;191:3986–9. 24043898 10.4049/jimmunol.1301549 PMC3819181

[400] Yang J, Zhao Y, Shi J, Shao F. Human NAIP and mouse NAIP1 recognize bacterial type III secretion needle protein for inflammasome activation. Proc Natl Acad Sci USA. 2013;110:14408–13. 23940371 10.1073/pnas.1306376110 PMC3761597

[401] Kortmann J, Brubaker SW, Monack DM. Cutting Edge: Inflammasome Activation in Primary Human Macrophages Is Dependent on Flagellin. J Immunol. 2015;195:815–9. 26109648 10.4049/jimmunol.1403100 PMC4505955

[402] Reyes Ruiz VM, Ramirez J, Naseer N, Palacio NM, Siddarthan IJ, Yan BM, et al. Broad detection of bacterial type III secretion system and flagellin proteins by the human NAIP/NLRC4 inflammasome. Proc Natl Acad Sci USA. 2017;114:13242–7. 29180436 10.1073/pnas.1710433114 PMC5740664

[403] Hu Z, Yan C, Liu P, Huang Z, Ma R, Zhang C, et al. Crystal structure of NLRC4 reveals its autoinhibition mechanism. Science. 2013;341:172–5. 23765277 10.1126/science.1236381

[404] Matico RE, Yu X, Miller R, Somani S, Ricketts MD, Kumar N, et al. Structural basis of the human NAIP/NLRC4 inflammasome assembly and pathogen sensing. Nat Struct Mol Biol. 2024;31:82–91. 38177670 10.1038/s41594-023-01143-z PMC10803261

[405] Paidimuddala B, Cao J, Nash G, Xie Q, Wu H, Zhang L. Mechanism of NAIP-NLRC4 inflammasome activation revealed by cryo-EM structure of unliganded NAIP5. Nat Struct Mol Biol. 2023;30:159–66. 36604500 10.1038/s41594-022-00889-2 PMC10576962

[406] Paidimuddala B, Cao J, Zhang L. Structural basis for flagellin-induced NAIP5 activation. Sci Adv. 2023;9:eadi8539. 38055825 10.1126/sciadv.adi8539 PMC10699770

[407] Zhang L, Chen S, Ruan J, Wu J, Tong AB, Yin Q, et al. Cryo-EM structure of the activated NAIP2-NLRC4 inflammasome reveals nucleated polymerization. Science. 2015;350:404–9. 26449474 10.1126/science.aac5789 PMC4640189

[408] Qu Y, Misaghi S, Izrael-Tomasevic A, Newton K, Gilmour LL, Lamkanfi M, et al. Phosphorylation of NLRC4 is critical for inflammasome activation. Nature. 2012;490:539–42. 22885697 10.1038/nature11429

[409] Wang S-b, Narendran S, Hirahara S, Varshney A, Pereira F, Apicella I, et al. DDX17 is an essential mediator of sterile NLRC4 inflammasome activation by retrotransposon RNAs. Sci Immunol. 2021;6:eabi4493. 34860583 10.1126/sciimmunol.abi4493 PMC8767314

[410] Suzuki S, Franchi L, He Y, Muñoz-Planillo R, Mimuro H, Suzuki T, et al. Shigella type III secretion protein MxiI is recognized by Naip2 to induce Nlrc4 inflammasome activation independently of Pkcd. elta PLoS Pathog. 2014;10:e1003926. 10.1371/journal.ppat.1003926 PMC3916413 24516390

[411] Tenthorey JL, Chavez RA, Thompson TW, Deets KA, Vance RE, Rauch I. NLRC4 inflammasome activation is NLRP3- and phosphorylation-independent during infection and does not protect from melanoma. J Exp Med. 2020;217:e20191736. 32342103 10.1084/jem.20191736 PMC7336302

[412] Qu Y, Misaghi S, Newton K, Maltzman A, Izrael-Tomasevic A, Arnott D, et al. NLRP3 recruitment by NLRC4 during Salmonella infection. J Exp Med. 2016;213:877–85. 27139490 10.1084/jem.20132234 PMC4886354

[413] Man SM, Tourlomousis P, Hopkins L, Monie TP, Fitzgerald KA, Bryant CE. Salmonella infection induces recruitment of Caspase-8 to the inflammasome to modulate IL-1beta production. J Immunol. 2013;191:5239–46. 24123685 10.4049/jimmunol.1301581 PMC3835177

[414] Man SM, Ekpenyong A, Tourlomousis P, Achouri S, Cammarota E, Hughes K, et al. Actin polymerization as a key innate immune effector mechanism to control Salmonella infection. Proc Natl Acad Sci USA. 2014;111:17588–93. 25422455 10.1073/pnas.1419925111 PMC4267384

[415] Freeman L, Guo H, David CN, Brickey WJ, Jha S, Ting JP. NLR members NLRC4 and NLRP3 mediate sterile inflammasome activation in microglia and astrocytes. J Exp Med. 2017;214:1351–70. 28404595 10.1084/jem.20150237 PMC5413320

[416] Romberg N, Al Moussawi K, Nelson-Williams C, Stiegler AL, Loring E, Choi M, et al. Mutation of NLRC4 causes a syndrome of enterocolitis and autoinflammation. Nat Genet. 2014;46:1135–9. 25217960 10.1038/ng.3066 PMC4177367

[417] Canna SW, de Jesus AA, Gouni S, Brooks SR, Marrero B, Liu Y, et al. An activating NLRC4 inflammasome mutation causes autoinflammation with recurrent macrophage activation syndrome. Nat Genet. 2014;46:1140–6. 25217959 10.1038/ng.3089 PMC4177369

[418] Kitamura A, Sasaki Y, Abe T, Kano H, Yasutomo K. An inherited mutation in NLRC4 causes autoinflammation in human and mice. J Exp Med. 2014;211:2385–96. 25385754 10.1084/jem.20141091 PMC4235634

[419] Kim YH, Lim JO, Kim JS, Kim BY, Pyun BJ, Lee SJ, et al. Protease allergen-induced HMGB1 contributes to NLRC4 inflammasome-mediated inflammation in experimental asthma. Allergy. 2023;78:1387–92. 36748908 10.1111/all.15668

[420] Kolb R, Phan L, Borcherding N, Liu Y, Yuan F, Janowski AM, et al. Obesity-associated NLRC4 inflammasome activation drives breast cancer progression. Nat Commun. 2016;7:13007. 27708283 10.1038/ncomms13007 PMC5059727

[421] Ohashi K, Wang Z, Yang YM, Billet S, Tu W, Pimienta M, et al. NOD-like receptor C4 inflammasome regulates the growth of colon cancer liver metastasis in NAFLD. Hepatology. 2019;70:1582–99. 31044438 10.1002/hep.30693 PMC6819206

[422] Hu B, Elinav E, Huber S, Booth CJ, Strowig T, Jin C, et al. Inflammation-induced tumorigenesis in the colon is regulated by caspase-1 and NLRC4. Proc Natl Acad Sci. 2010;107:21635–40. 21118981 10.1073/pnas.1016814108 PMC3003083

[423] Round JL, Mazmanian SK. The gut microbiota shapes intestinal immune responses during health and disease. Nat Rev Immunol. 2009;9:313–23. 19343057 10.1038/nri2515 PMC4095778

[424] Zitvogel L, Galluzzi L, Viaud S, Vétizou M, Daillère R, Merad M, et al. Cancer and the gut microbiota: an unexpected link. Sci Transl Med. 2015;7:271ps1–271ps1. 25609166 10.1126/scitranslmed.3010473 PMC4690201

[425] Robertson SJ, Lemire P, Maughan H, Goethel A, Turpin W, Bedrani L, et al. Comparison of cohousing and littermate methods for microbiota standardization in mouse models. Cell Rep. 2019;27:1910–1919.e2. 31067473 10.1016/j.celrep.2019.04.023

[426] Fernandes-Alnemri T, Yu JW, Datta P, Wu J, Alnemri ES. AIM2 activates the inflammasome and cell death in response to cytoplasmic DNA. Nature. 2009;458:509–13. 19158676 10.1038/nature07710 PMC2862225

[427] Hornung V, Ablasser A, Charrel-Dennis M, Bauernfeind F, Horvath G, Caffrey DR, et al. AIM2 recognizes cytosolic dsDNA and forms a caspase-1-activating inflammasome with ASC. Nature. 2009;458:514–8. 19158675 10.1038/nature07725 PMC2726264

[428] Roberts TL, Idris A, Dunn JA, Kelly GM, Burnton CM, Hodgson S, et al. HIN-200 proteins regulate caspase activation in response to foreign cytoplasmic DNA. Science. 2009;323:1057–60. 19131592 10.1126/science.1169841

[429] Bürckstümmer T, Baumann C, Blüml S, Dixit E, Dürnberger G, Jahn H, et al. An orthogonal proteomic-genomic screen identifies AIM2 as a cytoplasmic DNA sensor for the inflammasome. Nat Immunol. 2009;10:266–72. 19158679 10.1038/ni.1702

[430] Jin T, Perry A, Jiang J, Smith P, Curry JA, Unterholzner L, et al. Structures of the HIN domain:DNA complexes reveal ligand binding and activation mechanisms of the AIM2 inflammasome and IFI16 receptor. Immunity. 2012;36:561–71. 22483801 10.1016/j.immuni.2012.02.014 PMC3334467

[431] Jin T, Perry A, Smith P, Jiang J, Xiao TS. Structure of the absent in melanoma 2 (AIM2) pyrin domain provides insights into the mechanisms of AIM2 autoinhibition and inflammasome assembly. J Biol Chem. 2013;288:13225–35. 23530044 10.1074/jbc.M113.468033 PMC3650362

[432] Morrone SR, Matyszewski M, Yu X, Delannoy M, Egelman EH, Sohn J. Assembly driven activation of the AIM2 foreign-dsDNA sensor provides a polymerization template for downstream ASC. Nat Commun. 2015;6:7827. 26197926 10.1038/ncomms8827 PMC4525163

[433] Rathinam VA, Jiang Z, Waggoner SN, Sharma S, Cole LE, Waggoner L, et al. The AIM2 inflammasome is essential for host defense against cytosolic bacteria and DNA viruses. Nat Immunol. 2010;11:395–402. 20351692 10.1038/ni.1864 PMC2887480

[434] Fernandes-Alnemri T, Yu JW, Juliana C, Solorzano L, Kang S, Wu J, et al. The AIM2 inflammasome is critical for innate immunity to Francisella tularensis. Nat Immunol. 2010;11:385–93. 20351693 10.1038/ni.1859 PMC3111085

[435] Jones JW, Kayagaki N, Broz P, Henry T, Newton K, O'Rourke K, et al. Absent in melanoma 2 is required for innate immune recognition of Francisella tularensis. Proc Natl Acad Sci USA. 2010;107:9771–6. 20457908 10.1073/pnas.1003738107 PMC2906881

[436] Belhocine K, Monack DM. Francisella infection triggers activation of the AIM2 inflammasome in murine dendritic cells. Cell Microbiol. 2012;14:71–80. 21902795 10.1111/j.1462-5822.2011.01700.x PMC3240688

[437] Man SM, Karki R, Malireddi RK, Neale G, Vogel P, Yamamoto M, et al. The transcription factor IRF1 and guanylate-binding proteins target activation of the AIM2 inflammasome by Francisella infection. Nat Immunol. 2015;16:467–75. 25774715 10.1038/ni.3118 PMC4406811

[438] Meunier E, Wallet P, Dreier RF, Costanzo S, Anton L, Rühl S, et al. Guanylate-binding proteins promote activation of the AIM2 inflammasome during infection with Francisella novicida. Nat Immunol. 2015;16:476–84. 25774716 10.1038/ni.3119 PMC4568307

[439] Atianand MK, Duffy EB, Shah A, Kar S, Malik M, Harton JA. Francisella tularensis reveals a disparity between human and mouse NLRP3 inflammasome activation. J Biol Chem. 2011;286:39033–42. 21930705 10.1074/jbc.M111.244079 PMC3234728

[440] Feng S, Enosi Tuipulotu D, Pandey A, Jing W, Shen C, Ngo C, et al. Pathogen-selective killing by guanylate-binding proteins as a molecular mechanism leading to inflammasome signaling. Nat Commun. 2022;13:4395. 35906252 10.1038/s41467-022-32127-0 PMC9338265

[441] Kim S, Bauernfeind F, Ablasser A, Hartmann G, Fitzgerald KA, Latz E, et al. Listeria monocytogenes is sensed by the NLRP3 and AIM2 inflammasome. Eur J Immunol. 2010;40:1545–51. 20333626 10.1002/eji.201040425 PMC3128919

[442] Sauer JD, Witte CE, Zemansky J, Hanson B, Lauer P, Portnoy DA. Listeria monocytogenes triggers AIM2-mediated pyroptosis upon infrequent bacteriolysis in the macrophage cytosol. Cell Host Microbe. 2010;7:412–9. 20417169 10.1016/j.chom.2010.04.004 PMC2947455

[443] Warren SE, Armstrong A, Hamilton MK, Mao DP, Leaf IA, Miao EA, et al. Cutting edge: Cytosolic bacterial DNA activates the inflammasome via Aim2. J Immunol. 2010;185:818–21. 20562263 10.4049/jimmunol.1000724 PMC2993756

[444] Wu J, Fernandes-Alnemri T, Alnemri ES. Involvement of the AIM2, NLRC4, and the NLRP3 inflammasome in caspase-1 activation by Listeria monocytogenes. J Clin Immunol. 2010;30:693–702. 20490635 10.1007/s10875-010-9425-2 PMC3321545

[445] Lee S, Karki R, Wang Y, Nguyen LN, Kalathur RC, Kanneganti TD. AIM2 forms a complex with pyrin and ZBP1 to drive PANoptosis and host defense. Nature. 2021;597:415–9. 34471287 10.1038/s41586-021-03875-8 PMC8603942

[446] Zhou Q, Zhang L, Lin Q, Liu H, Ye G, Liu X, et al. Pseudorabies Virus Infection Activates the TLR-NF-kappaB Axis and AIM2 Inflammasome To Enhance Inflammatory Responses in Mice. J Virol. 2023;97:0000323. 10.1128/jvi.00003-23 PMC10062126 36877049

[447] Kalantari P, DeOliveira RB, Chan J, Corbett Y, Rathinam V, Stutz A, et al. Dual engagement of the NLRP3 and AIM2 inflammasomes by Plasmodium-derived hemozoin and DNA during malaria. Cell Rep. 2014;6:196–210. 24388751 10.1016/j.celrep.2013.12.014 PMC4105362

[448] Marques-da-Silva C, Poudel B, Baptista RP, Peissig K, Hancox LS, Shiau JC, et al. AIM2 sensors mediate immunity to Plasmodium infection in hepatocytes. Proc Natl Acad Sci USA. 2023;120:e2210181120. 36595704 10.1073/pnas.2210181120 PMC9926219

[449] Oh S, Lee J, Oh J, Yu G, Ryu H, Kim D, et al. Integrated NLRP3, AIM2, NLRC4, Pyrin inflammasome activation and assembly drive PANoptosis. Cell Mol Immunol. 2023;20:1513–26. 38008850 10.1038/s41423-023-01107-9 PMC10687226

[450] Brunette RL, Young JM, Whitley DG, Brodsky IE, Malik HS, Stetson DB. Extensive evolutionary and functional diversity among mammalian AIM2-like receptors. J Exp Med. 2012;209:1969–83. 23045604 10.1084/jem.20121960 PMC3478938

[451] Ngo CC, Man SM. Mechanisms and functions of guanylate-binding proteins and related interferon-inducible GTPases: Roles in intracellular lysis of pathogens. Cell Microbiol. 2017;19:e12791. 10.1111/cmi.12791 28975702

[452] Costa Franco MM, Marim F, Guimarães ES, Assis N, Cerqueira DM, Alves-Silva J, et al. Brucella abortus Triggers a cGAS-Independent STING Pathway To Induce Host Protection That Involves Guanylate-Binding Proteins and Inflammasome Activation. J Immunol. 2018;200:607–22. 29203515 10.4049/jimmunol.1700725 PMC5760291

[453] Gomes MT, Campos PC, Oliveira FS, Corsetti PP, Bortoluci KR, Cunha LD, et al. Critical role of ASC inflammasomes and bacterial type IV secretion system in caspase-1 activation and host innate resistance to Brucella abortus infection. J Immunol. 2013;190:3629–38. 23460746 10.4049/jimmunol.1202817

[454] Costa Franco MMS, Marim FM, Alves-Silva J, Cerqueira D, Rungue M, Tavares IP, et al. AIM2 senses Brucella abortus DNA in dendritic cells to induce IL-1beta secretion, pyroptosis and resistance to bacterial infection in mice. Microbes Infect. 2019;21:85–93. 30248400 10.1016/j.micinf.2018.09.001 PMC6430705

[455] Pierini R, Perret M, Djebali S, Juruj C, Michallet MC, Förster I, et al. ASC controls IFN-gamma levels in an IL-18-dependent manner in caspase-1-deficient mice infected with Francisella novicida. J Immunol. 2013;191:3847–57. 23975862 10.4049/jimmunol.1203326

[456] Saiga H, Kitada S, Shimada Y, Kamiyama N, Okuyama M, Makino M, et al. Critical role of AIM2 in Mycobacterium tuberculosis infection. Int Immunol. 2012;24:637–44. 22695634 10.1093/intimm/dxs062

[457] Hanamsagar R, Aldrich A, Kielian T. Critical role for the AIM2 inflammasome during acute CNS bacterial infection. J Neurochem. 2014;129:704–11. 24484406 10.1111/jnc.12669 PMC3999210

[458] Zhu Q, Man SM, Karki R, Malireddi R, Kanneganti TD. Detrimental Type I Interferon Signaling Dominates Protective AIM2 Inflammasome Responses during Francisella novicida Infection. Cell Rep. 2018;22:3168–74. 29562174 10.1016/j.celrep.2018.02.096 PMC6204211

[459] Tanishita Y, Sekiya H, Inohara N, Tsuchiya K, Mitsuyama M, Núñez G, et al. Listeria toxin promotes phosphorylation of the inflammasome adaptor ASC through Lyn and Syk to exacerbate pathogen expansion. Cell Rep. 2022;38:110414. 35196496 10.1016/j.celrep.2022.110414

[460] Man SM, Karki R, Kanneganti TD. AIM2 inflammasome in infection, cancer, and autoimmunity: Role in DNA sensing, inflammation, and innate immunity. Eur J Immunol. 2016;46:269–80. 26626159 10.1002/eji.201545839 PMC4758349

[461] Li Y, Yang Y, Li T, Wang Z, Gao C, Deng R, et al. Activation of AIM2 by hepatitis B virus results in antiviral immunity that suppresses hepatitis C virus during coinfection. J Virol. 2023;97:e0109023. 37787533 10.1128/jvi.01090-23 PMC10617567

[462] Reinholz M, Kawakami Y, Salzer S, Kreuter A, Dombrowski Y, Koglin S, et al. HPV16 activates the AIM2 inflammasome in keratinocytes. Arch Dermatol Res. 2013;305:723–32. 23764897 10.1007/s00403-013-1375-0

[463] Huang Y, Ma D, Huang H, Lu Y, Liao Y, Liu L, et al. Interaction between HCMV pUL83 and human AIM2 disrupts the activation of the AIM2 inflammasome. Virol J. 2017;14:34. 28219398 10.1186/s12985-016-0673-5 PMC5319029

[464] Maruzuru Y, Ichinohe T, Sato R, Miyake K, Okano T, Suzuki T, et al. Herpes Simplex Virus 1 VP22 Inhibits AIM2-Dependent Inflammasome Activation to Enable Efficient Viral Replication. Cell Host Microbe. 2018;23:254–65.e7. 29447697 10.1016/j.chom.2017.12.014

[465] Schattgen SA, Gao G, Kurt-Jones EA, Fitzgerald KA. Cutting Edge: DNA in the Lung Microenvironment during Influenza Virus Infection Tempers Inflammation by Engaging the DNA Sensor AIM2. J Immunol. 2016;196:29–33. 26590313 10.4049/jimmunol.1501048 PMC4793160

[466] Zhang H, Luo J, Alcorn JF, Chen K, Fan S, Pilewski J, et al. AIM2 Inflammasome Is Critical for Influenza-Induced Lung Injury and Mortality. J Immunol. 2017;198:4383–93. 28424239 10.4049/jimmunol.1600714 PMC5439025

[467] Junqueira C, Crespo Â, Ranjbar S, de Lacerda LB, Lewandrowski M, Ingber J, et al. FcgammaR-mediated SARS-CoV-2 infection of monocytes activates inflammation. Nature. 2022;606:576–84. 35385861 10.1038/s41586-022-04702-4 PMC10071495

[468] Ekchariyawat P, Hamel R, Bernard E, Wichit S, Surasombatpattana P, Talignani L, et al. Inflammasome signaling pathways exert antiviral effect against Chikungunya virus in human dermal fibroblasts. Infect Genet Evol. 2015;32:401–8. 25847693 10.1016/j.meegid.2015.03.025

[469] Moriyama M, Koshiba T, Ichinohe T. Influenza A virus M2 protein triggers mitochondrial DNA-mediated antiviral immune responses. Nat Commun. 2019;10:4624. 31604929 10.1038/s41467-019-12632-5 PMC6789137

[470] Zhu Q, Man SM, Gurung P, Liu Z, Vogel P, Lamkanfi M, et al. Cutting edge: STING mediates protection against colorectal tumorigenesis by governing the magnitude of intestinal inflammation. J Immunol. 2014;193:4779–82. 25320273 10.4049/jimmunol.1402051 PMC4308418

[471] Man SM, Zhu Q, Zhu L, Liu Z, Karki R, Malik A, et al. Critical Role for the DNA Sensor AIM2 in Stem Cell Proliferation and Cancer. Cell. 2015;162:45–58. 26095253 10.1016/j.cell.2015.06.001 PMC4491002

[472] Wilson JE, Petrucelli AS, Chen L, Koblansky AA, Truax AD, Oyama Y, et al. Inflammasome-independent role of AIM2 in suppressing colon tumorigenesis via DNA-PK and Akt. Nat Med. 2015;21:906–13. 26107252 10.1038/nm.3908 PMC4529369

[473] Pandey A, Shen C, Feng S, Enosi Tuipulotu D, Ngo C, Liu C, et al. Ku70 senses cytosolic DNA and assembles a tumor-suppressive signalosome. Sci Adv. 2024;10:eadh3409. 38277448 10.1126/sciadv.adh3409 PMC10816715

[474] Karki R, Man SM, Malireddi R, Kesavardhana S, Zhu Q, Burton AR, et al. NLRC3 is an inhibitory sensor of PI3K-mTOR pathways in cancer. Nature. 2016;540:583–7. 27951586 10.1038/nature20597 PMC5468516

[475] Di Micco A, Frera G, Lugrin J, Jamilloux Y, Hsu ET, Tardivel A, et al. AIM2 inflammasome is activated by pharmacological disruption of nuclear envelope integrity. Proc Natl Acad Sci USA. 2016;113:E4671–80. 27462105 10.1073/pnas.1602419113 PMC4987819

[476] Wang LQ, Liu T, Yang S, Sun L, Zhao ZY, Li LY, et al. Perfluoroalkyl substance pollutants activate the innate immune system through the AIM2 inflammasome. Nat Commun. 2021;12:2915. 34006824 10.1038/s41467-021-23201-0 PMC8131593

[477] Ratsimandresy RA, Indramohan M, Dorfleutner A, Stehlik C. The AIM2 inflammasome is a central regulator of intestinal homeostasis through the IL-18/IL-22/STAT3 pathway. Cell Mol Immunol. 2017;14:127–42. 27524110 10.1038/cmi.2016.35 PMC5214942

[478] Yang Y, Zhang M, Jin C, Ding Y, Yang M, Wang R, et al. Absent in melanoma 2 suppresses epithelial–mesenchymal transition via Akt and inflammasome pathways in human colorectal cancer cells. J Cell Biochem. 2019;120:17744–56. 31210372 10.1002/jcb.29040

[479] Xu M, Wang J, Li H, Zhang Z, Cheng Z. AIM2 inhibits colorectal cancer cell proliferation and migration through suppression of Gli1. Aging. 2020;13:1017–31. 33291082 10.18632/aging.202226 PMC7835022

[480] Patsos G, Germann A, Gebert J, Dihlmann S. Restoration of absent in melanoma 2 (AIM2) induces G2/M cell cycle arrest and promotes invasion of colorectal cancer cells. Int J Cancer. 2010;126:1838–49. 19795419 10.1002/ijc.24905

[481] Chai D, Shan H, Wang G, Li H, Fang L, Song J, et al. AIM2 is a potential therapeutic target in human renal carcinoma and suppresses its invasion and metastasis by enhancing autophagy induction. Exp Cell Res. 2018;370:561–70. 30031129 10.1016/j.yexcr.2018.07.021

[482] Chai D, Zhang Z, Shi SY, Qiu D, Zhang C, Wang G, et al. Absent in melanoma 2-mediating M1 macrophages facilitate tumor rejection in renal carcinoma. Transl Oncol. 2021;14:101018. 33493800 10.1016/j.tranon.2021.101018 PMC7823216

[483] Chai D, Qiu D, Zhang Z, Yuchen Shi S, Wang G, Fang L, et al. Absent in melanoma 2 enhances antitumor effects of CAIX promotor controlled conditionally replicative adenovirus in renal cancer. J Cell Mol Med. 2020;24:10744–55. 32725966 10.1111/jcmm.15697 PMC7521288

[484] So D, Shin HW, Kim J, Lee M, Myeong J, Chun YS, et al. Cervical cancer is addicted to SIRT1 disarming the AIM2 antiviral defense. Oncogene. 2018;37:5191–204. 29844574 10.1038/s41388-018-0339-4

[485] Wang D, Zou J, Dai J, Cheng Z. Absent in melanoma 2 suppresses gastric cancer cell proliferation and migration via inactivation of AKT signaling pathway. Sci Rep. 2021;11:8235. 33859277 10.1038/s41598-021-87744-4 PMC8050218

[486] Zheng J, Liu C, Shi J, Wen K, Wang X. AIM2 inhibits the proliferation, invasion and migration, and promotes the apoptosis of osteosarcoma cells by inactivating the PI3K/AKT/mTOR signaling pathway. Mol Med Rep. 2022;25:53. 34913077 10.3892/mmr.2021.12569 PMC8711022

[487] Chen IF, Ou-Yang F, Hung JY, Liu JC, Wang H, Wang SC, et al. AIM2 suppresses human breast cancer cell proliferation in vitro and mammary tumor growth in a mouse model. Mol Cancer Ther. 2006;5:1–7. 16432157 10.1158/1535-7163.MCT-05-0310

[488] Liu ZY, Yi J, Liu FE. The molecular mechanism of breast cancer apoptosis induction by absent in melanoma (AIM2). Int J Clin Exp Med. 2015;8:14750–8. 26628957 PMC4658846

[489] Su S, Zhao J, Xing Y, Zhang X, Liu J, Ouyang Q, et al. Immune Checkpoint Inhibition Overcomes ADCP-Induced Immunosuppression by Macrophages. Cell. 2018;175:442–457.e23. 30290143 10.1016/j.cell.2018.09.007

[490] Li Y, Wang W, Li A, Huang W, Chen S, Han F, et al. Dihydroartemisinin induces pyroptosis by promoting the AIM2/caspase-3/DFNA5 axis in breast cancer cells. Chem Biol Interact. 2021;340:109434. 33689708 10.1016/j.cbi.2021.109434

[491] Zheng P, Xiao W, Zhang J, Zheng X, Jiang J. The role of AIM2 in human hepatocellular carcinoma and its clinical significance. Pathol Res Pr. 2023;245:154454. 10.1016/j.prp.2023.154454 37060822

[492] Ma X, Guo P, Qiu Y, Mu K, Zhu L, Zhao W, et al. Loss of AIM2 expression promotes hepatocarcinoma progression through activation of mTOR-S6K1 pathway. Oncotarget. 2016;7:36185–97. 27167192 10.18632/oncotarget.9154 PMC5094992

[493] Qi M, Dai D, Liu J, Li Z, Liang P, Wang Y, et al. AIM2 promotes the development of non-small cell lung cancer by modulating mitochondrial dynamics. Oncogene. 2020;39:2707–23. 32005973 10.1038/s41388-020-1176-9

[494] Ye H, Yu W, Li Y, Bao X, Ni Y, Chen X, et al. AIM2 fosters lung adenocarcinoma immune escape by modulating PD-L1 expression in tumor-associated macrophages via JAK/STAT3. Hum Vaccin Immunother. 2023;19:2269790. 37877820 10.1080/21645515.2023.2269790 PMC10601527

[495] Zheng JQ, Lin CH, Lee HH, Chang WM, Li LJ, Su CY, et al. AIM2 upregulation promotes metastatic progression and PD-L1 expression in lung adenocarcinoma. Cancer Sci. 2023;114:306–20. 36104978 10.1111/cas.15584 PMC9807530

[496] Nakamura Y, Nakahata S, Kondo Y, Izumi A, Yamamoto K, Ichikawa T, et al. Overexpression of absent in melanoma 2 in oral squamous cell carcinoma contributes to tumor progression. Biochem Biophys Res Commun. 2019;509:82–88. 30587341 10.1016/j.bbrc.2018.12.066

[497] Chiu HW, Lee HL, Lee HH, Lu HW, Lin KY, Lin YF, et al. AIM2 promotes irradiation resistance, migration ability and PD-L1 expression through STAT1/NF-kappaB activation in oral squamous cell carcinoma. J Transl Med. 2024;22:13. 38166970 10.1186/s12967-023-04825-w PMC10762966

[498] Tan X, Chen D, Guo S, Wang Y, Zou Y, Wu Z, et al. Molecular stratification by BCL2A1 and AIM2 provides additional prognostic value in penile squamous cell carcinoma. Theranostics. 2021;11:1364–76. 33391539 10.7150/thno.51725 PMC7738875

[499] Martínez-Cardona C, Lozano-Ruiz B, Bachiller V, Peiró G, Algaba-Chueca F, Gómez-Hurtado I, et al. AIM2 deficiency reduces the development of hepatocellular carcinoma in mice. Int J Cancer. 2018;143:2997–3007. 30133699 10.1002/ijc.31827

[500] Zhang M, Jin C, Yang Y, Wang K, Zhou Y, Zhou Y, et al. AIM2 promotes non-small cell lung cancer cell growth through inflammasome-dependent pathway. J Cell Physiol. 2019;234:20161–73. 30953357 10.1002/jcp.28617

[501] Chai D, Liu N, Li H, Wang G, Song J, Fang L, et al. H1/pAIM2 nanoparticles exert antitumor effects that is associated with the inflammasome activation in renal carcinoma. J Cell Mol Med. 2018;22:5670–81. 30160343 10.1111/jcmm.13842 PMC6201339

[502] Shah S, Qin S, Luo Y, Huang Y, Jing R, Shah JN, et al. AIM2 Inhibits BRAF-Mutant Colorectal Cancer Growth in a Caspase-1-Dependent Manner. Front Cell Dev Biol. 2021;9:588278. 33842454 10.3389/fcell.2021.588278 PMC8027362

[503] Farshchian M, Nissinen L, Siljamäki E, Riihilä P, Piipponen M, Kivisaari A, et al. Tumor cell-specific AIM2 regulates growth and invasion of cutaneous squamous cell carcinoma. Oncotarget. 2017;8:45825–36. 28526809 10.18632/oncotarget.17573 PMC5542230

[504] Li L, Mao R, Yuan S, Xie Q, Meng J, Gu Y, et al. NCF4 attenuates colorectal cancer progression by modulating inflammasome activation and immune surveillance. Nat Commun. 2024;15:5170. 38886341 10.1038/s41467-024-49549-7 PMC11183137

[505] Lu A, Wu S, Niu J, Cui M, Chen M, Clapp WL, et al. Aim2 Couples With Ube2i for Sumoylation-Mediated Repression of Interferon Signatures in Systemic Lupus Erythematosus. Arthritis Rheumatol. 2021;73:1467–77. 33559374 10.1002/art.41677 PMC8324518

[506] Zhang W, Cai Y, Xu W, Yin Z, Gao X, Xiong S. AIM2 facilitates the apoptotic DNA-induced systemic lupus erythematosus by arbitrating macrophage functional maturation. J Clin Immunol. 2013;33:925–37. 23479181 10.1007/s10875-013-9881-6

[507] Méndez-Frausto G, Medina-Rosales MN, Uresti-Rivera EE, Baranda-Cándido L, Zapata-Zúñiga M, Bastián Y, et al. Expression and activity of AIM2-inflammasome in rheumatoid arthritis patients. Immunobiology. 2020;225:151880. 31836304 10.1016/j.imbio.2019.11.015

[508] Dombrowski Y, Peric M, Koglin S, Kammerbauer C, Göss C, Anz D, et al. Cytosolic DNA triggers inflammasome activation in keratinocytes in psoriatic lesions. Sci Transl Med. 2011;3:82ra38. 21562230 10.1126/scitranslmed.3002001 PMC3235683

[509] Cao T, Yuan X, Fang H, Chen J, Xue K, Li Z, et al. Neutrophil extracellular traps promote keratinocyte inflammation via AIM2 inflammasome and AIM2-XIAP in psoriasis. Exp Dermatol. 2023;32:368–78. 36401800 10.1111/exd.14711

[510] Vakrakou AG, Svolaki IP, Evangelou K, Gorgoulis VG, Manoussakis MN. Cell-autonomous epithelial activation of AIM2 (absent in melanoma-2) inflammasome by cytoplasmic DNA accumulations in primary Sjogren’s syndrome. J Autoimmun. 2020;108:102381. 31919014 10.1016/j.jaut.2019.102381

[511] Du L, Wang X, Chen S, Guo X. The AIM2 inflammasome: A novel biomarker and target in cardiovascular disease. Pharm Res. 2022;186:106533. 10.1016/j.phrs.2022.106533 36332811

[512] Wang L, Sun L, Byrd KM, Ko CC, Zhao Z, Fang J. AIM2 Inflammasome’s First Decade of Discovery: Focus on Oral Diseases. Front Immunol. 2020;11:1487. 32903550 10.3389/fimmu.2020.01487 PMC7438472

[513] Lozano-Ruiz B, Gonzalez-Navajas JM. The Emerging Relevance of AIM2 in Liver Disease. Int J Mol Sci. 2020;21:6535. 32906750 10.3390/ijms21186535 PMC7555176

[514] Wu PJ, Hung YF, Liu HY, Hsueh YP. Deletion of the Inflammasome Sensor Aim2 Mitigates Abeta Deposition and Microglial Activation but Increases Inflammatory Cytokine Expression in an Alzheimer Disease Mouse Model. Neuroimmunomodulation. 2017;24:29–39. 28618410 10.1159/000477092

[515] Rui WJ, Li S, Yang L, Liu Y, Fan Y, Hu YC, et al. Microglial AIM2 alleviates antiviral-related neuro-inflammation in mouse models of Parkinson’s disease. Glia. 2022;70:2409–25. 35959803 10.1002/glia.24260

[516] Hu B, Jin C, Li HB, Tong J, Ouyang X, Cetinbas NM, et al. The DNA-sensing AIM2 inflammasome controls radiation-induced cell death and tissue injury. Science. 2016;354:765–8. 27846608 10.1126/science.aaf7532 PMC5640175

[517] Gao J, Peng S, Shan X, Deng G, Shen L, Sun J, et al. Inhibition of AIM2 inflammasome-mediated pyroptosis by Andrographolide contributes to amelioration of radiation-induced lung inflammation and fibrosis. Cell Death Dis. 2019;10:957. 31862870 10.1038/s41419-019-2195-8 PMC6925222

[518] Man SM, Karki R, Kanneganti TD. DNA-sensing inflammasomes: regulation of bacterial host defense and the gut microbiota. Pathog Dis. 2016;74:ftw028. 27056948 10.1093/femspd/ftw028 PMC5985483

[519] Kaminski JJ, Schattgen SA, Tzeng TC, Bode C, Klinman DM, Fitzgerald KA. Synthetic oligodeoxynucleotides containing suppressive TTAGGG motifs inhibit AIM2 inflammasome activation. J Immunol. 2013;191:3876–83. 23986531 10.4049/jimmunol.1300530 PMC3878640

[520] Lee SB, Kang JH, Sim EJ, Jung YR, Kim JH, Hillman PF, et al. Cornus officinalis Seed Extract Inhibits AIM2-Inflammasome Activation and Attenuates Imiquimod-Induced Psoriasis-like Skin Inflammation. Int J Mol Sci. 2023;24:5653. 36982727 10.3390/ijms24065653 PMC10051512

[521] Jiao Y, Nan J, Mu B, Zhang Y, Zhou N, Yang S, et al. Discovery of a novel and potent inhibitor with differential species-specific effects against NLRP3 and AIM2 inflammasome-dependent pyroptosis. Eur J Med Chem. 2022;232:114194. 35183871 10.1016/j.ejmech.2022.114194

[522] Green JP, El-Sharkawy LY, Roth S, Zhu J, Cao J, Leach AG, et al. Discovery of an inhibitor of DNA-driven inflammation that preferentially targets the AIM2 inflammasome. iScience. 2023;26:106758. 37216118 10.1016/j.isci.2023.106758 PMC10193008

[523] Hong Y, Lee SO, Oh C, Kang K, Ryoo J, Kim D, et al. USP21 Deubiquitinase Regulates AIM2 Inflammasome Activation. J Immunol. 2021;207:1926–36. 34470856 10.4049/jimmunol.2100449

[524] Yin Q, Sester DP, Tian Y, Hsiao YS, Lu A, Cridland JA, et al. Molecular mechanism for p202-mediated specific inhibition of AIM2 inflammasome activation. Cell Rep. 2013;4:327–39. 23850291 10.1016/j.celrep.2013.06.024 PMC3760141

[525] Wang PH, Ye ZW, Deng JJ, Siu KL, Gao WW, Chaudhary V, et al. Inhibition of AIM2 inflammasome activation by a novel transcript isoform of IFI16. EMBO Rep. 2018;19:e45737. 30104205 10.15252/embr.201845737 PMC6172465

[526] Centola M, Wood G, Frucht DM, Galon J, Aringer M, Farrell C, et al. The gene for familial Mediterranean fever, MEFV, is expressed in early leukocyte development and is regulated in response to inflammatory mediators. Blood. 2000;95:3223–31. 10807793

[527] French FMFC. A candidate gene for familial Mediterranean fever. Nat Genet. 1997;17:25–31. 9288094 10.1038/ng0997-25

[528] Tufan A, Lachmann HJ. Familial Mediterranean fever, from pathogenesis to treatment: a contemporary review. Turk J Med Sci. 2020;50:1591–610. 32806879 10.3906/sag-2008-11 PMC7672358

[529] D'cruz AA, Babon JJ, Norton RS, Nicola NA, Nicholson SE. Structure and function of the SPRY/B30.2 domain proteins involved in innate immunity. Protein Sci. 2013;22:1–10. 23139046 10.1002/pro.2185 PMC3575854

[530] Perfetto L, Gherardini PF, Davey NE, Diella F, Helmer-Citterich M, Cesareni G. Exploring the diversity of SPRY/B30.2-mediated interactions. Trends Biochem Sci. 2013;38:38–46. 23164942 10.1016/j.tibs.2012.10.001

[531] Weinert C, Morger D, Djekic A, Grütter MG, Mittl PR. Crystal structure of TRIM20 C-terminal coiled-coil/B30.2 fragment: implications for the recognition of higher order oligomers. Sci Rep. 2015;5:10819. 26043233 10.1038/srep10819 PMC4455283

[532] Richards N, Schaner P, Diaz A, Stuckey J, Shelden E, Wadhwa A, et al. Interaction between pyrin and the apoptotic speck protein (ASC) modulates ASC-induced apoptosis. J Biol Chem. 2001;276:39320–9. 11498534 10.1074/jbc.M104730200

[533] Chae JJ, Wood G, Masters SL, Richard K, Park G, Smith BJ, et al. The B30.2 domain of pyrin, the familial Mediterranean fever protein, interacts directly with caspase-1 to modulate IL-1beta production. Proc Natl Acad Sci USA. 2006;103:9982–7. 16785446 10.1073/pnas.0602081103 PMC1479864

[534] Hesker PR, Nguyen M, Kovarova M, Ting JP, Koller BH. Genetic loss of murine pyrin, the Familial Mediterranean Fever protein, increases interleukin-1beta levels. PLoS One. 2012;7:e51105. 23226472 10.1371/journal.pone.0051105 PMC3511413

[535] Chae JJ, Cho YH, Lee GS, Cheng J, Liu PP, Feigenbaum L, et al. Gain-of-function Pyrin mutations induce NLRP3 protein-independent interleukin-1beta activation and severe autoinflammation in mice. Immunity. 2011;34:755–68. 21600797 10.1016/j.immuni.2011.02.020 PMC3129608

[536] Gavrilin MA, Abdelaziz DH, Mostafa M, Abdulrahman BA, Grandhi J, Akhter A, et al. Activation of the pyrin inflammasome by intracellular Burkholderia cenocepacia. J Immunol. 2012;188:3469–77. 22368275 10.4049/jimmunol.1102272 PMC3482472

[537] Xu H, Yang J, Gao W, Li L, Li P, Zhang L, et al. Innate immune sensing of bacterial modifications of Rho GTPases by the Pyrin inflammasome. Nature. 2014;513:237–41. 24919149 10.1038/nature13449

[538] Hodge RG, Ridley AJ. Regulating Rho GTPases and their regulators. Nat Rev Mol Cell Biol. 2016;17:496–510. 27301673 10.1038/nrm.2016.67

[539] Aktories K. Bacterial protein toxins that modify host regulatory GTPases. Nat Rev Microbiol. 2011;9:487–98. 21677684 10.1038/nrmicro2592

[540] Xu N, Jiang J, Jiang F, Dong G, Meng L, Wang M, et al. CircCDC42-encoded CDC42-165aa regulates macrophage pyroptosis in Klebsiella pneumoniae infection through Pyrin inflammasome activation. Nat Commun. 2024;15:5730. 38977695 10.1038/s41467-024-50154-x PMC11231140

[541] Gao W, Yang J, Liu W, Wang Y, Shao F. Site-specific phosphorylation and microtubule dynamics control Pyrin inflammasome activation. Proc Natl Acad Sci USA. 2016;113:E4857–66. p. 27482109 10.1073/pnas.1601700113 PMC4995971

[542] Park YH, Wood G, Kastner DL, Chae JJ. Pyrin inflammasome activation and RhoA signaling in the autoinflammatory diseases FMF and HIDS. Nat Immunol. 2016;17:914–21. 27270401 10.1038/ni.3457 PMC4955684

[543] Kim ML, Chae JJ, Park YH, De Nardo D, Stirzaker RA, Ko HJ, et al. Aberrant actin depolymerization triggers the pyrin inflammasome and autoinflammatory disease that is dependent on IL-18, not IL-1b. eta J Exp Med. 2015;212:927–38. 10.1084/jem.20142384 PMC4451132 26008898

[544] Masters SL, Lagou V, Jéru I, Baker PJ, Van Eyck L, Parry DA, et al. Familial autoinflammation with neutrophilic dermatosis reveals a regulatory mechanism of pyrin activation. Sci Transl Med. 2016;8:332ra45. 27030597 10.1126/scitranslmed.aaf1471

[545] Chung LK, Park YH, Zheng Y, Brodsky IE, Hearing P, Kastner DL, et al. The Yersinia Virulence Factor YopM Hijacks Host Kinases to Inhibit Type III Effector-Triggered Activation of the Pyrin Inflammasome. Cell Host Microbe. 2016;20:296–306. 27569559 10.1016/j.chom.2016.07.018 PMC5025386

[546] Ratner D, Orning MP, Proulx MK, Wang D, Gavrilin MA, Wewers MD, et al. The Yersinia pestis Effector YopM Inhibits Pyrin Inflammasome Activation. PLoS Pathog. 2016;12:e1006035. 27911947 10.1371/journal.ppat.1006035 PMC5135138

[547] Park YH, Remmers EF, Lee W, Ombrello AK, Chung LK, Shilei Z, et al. Ancient familial Mediterranean fever mutations in human pyrin and resistance to Yersinia pestis. Nat Immunol. 2020;21:857–67. 32601469 10.1038/s41590-020-0705-6 PMC7381377

[548] Magupalli VG, Negro R, Tian Y, Hauenstein AV, Di Caprio G, Skillern W, et al. HDAC6 mediates an aggresome-like mechanism for NLRP3 and pyrin inflammasome activation. Science. 2020;369:eaas8995. 32943500 10.1126/science.aas8995 PMC7814939

[549] Wang L, Shi S, Unterreiner A, Kapetanovic R, Ghosh S, Sanchez J, et al. HDAC6/aggresome processing pathway importance for inflammasome formation is context dependent. J Biol Chem. 2024;300:105638. 38199570 10.1016/j.jbc.2024.105638 PMC10850954

[550] Tsujimoto K, Jo T, Nagira D, Konaka H, Park JH, Yoshimura SI, et al. The lysosomal Ragulator complex activates NLRP3 inflammasome in vivo via HDAC6. EMBO J. 2023;42:e111389. 36444797 10.15252/embj.2022111389 PMC9811619

[551] Sharma D, Malik A, Guy CS, Karki R, Vogel P, Kanneganti TD. Pyrin Inflammasome Regulates Tight Junction Integrity to Restrict Colitis and Tumorigenesis. Gastroenterology. 2018;154:948–964 e8. 29203393 10.1053/j.gastro.2017.11.276 PMC5847456

[552] Taskiran EZ, Cetinkaya A, Balci-Peynircioglu B, Akkaya YZ, Yilmaz E. The effect of colchicine on pyrin and pyrin interacting proteins. J Cell Biochem. 2012;113:3536–46. 22730186 10.1002/jcb.24231

[553] Van Gorp H, Saavedra PH, de Vasconcelos NM, Van Opdenbosch N, Vande Walle L, Matusiak M, et al. Familial Mediterranean fever mutations lift the obligatory requirement for microtubules in Pyrin inflammasome activation. Proc Natl Acad Sci USA. 2016;113:14384–9. 27911804 10.1073/pnas.1613156113 PMC5167202

[554] Yu JW, Wu J, Zhang Z, Datta P, Ibrahimi I, Taniguchi S, et al. Cryopyrin and pyrin activate caspase-1, but not NF-kappaB, via ASC oligomerization. Cell Death Differ. 2006;13:236–49. 16037825 10.1038/sj.cdd.4401734

[555] Chirita D, Bronnec P, Magnotti F, Dalmon S, Martin A, Popoff M, et al. Mutations in the B30.2 and the central helical scaffold domains of pyrin differentially affect inflammasome activation. Cell Death Dis. 2023;14:213. 36966139 10.1038/s41419-023-05745-9 PMC10039897

[556] Kuhns DB, Fink DL, Choi U, Sweeney C, Lau K, Priel DL, et al. Cytoskeletal abnormalities and neutrophil dysfunction in WDR1 deficiency. Blood. 2016;128:2135–43. 27557945 10.1182/blood-2016-03-706028 PMC5084607

[557] Munoz MA, Jurczyluk J, Mehr S, Chai RC, Arts R, Sheu A, et al. Defective protein prenylation is a diagnostic biomarker of mevalonate kinase deficiency. J Allergy Clin Immunol. 2017;140:873–875.e6. 28501347 10.1016/j.jaci.2017.02.033

[558] Shoham NG, Centola M, Mansfield E, Hull KM, Wood G, Wise CA, et al. Pyrin binds the PSTPIP1/CD2BP1 protein, defining familial Mediterranean fever and PAPA syndrome as disorders in the same pathway. Proc Natl Acad Sci USA. 2003;100:13501–6. 14595024 10.1073/pnas.2135380100 PMC263843

[559] Leung YY, Yao Hui LL, Kraus VB. Colchicine-Update on mechanisms of action and therapeutic uses. Semin Arthritis Rheum. 2015;45:341–50. 26228647 10.1016/j.semarthrit.2015.06.013 PMC4656054

[560] Ben-Zvi I, Kukuy O, Giat E, Pras E, Feld O, Kivity S, et al. Anakinra for Colchicine-Resistant Familial Mediterranean Fever: A Randomized, Double-Blind, Placebo-Controlled Trial. Arthritis Rheumatol. 2017;69:854–62. 27860460 10.1002/art.39995

[561] Ozen S, Bilginer Y. A clinical guide to autoinflammatory diseases: familial Mediterranean fever and next-of-kin. Nat Rev Rheumatol. 2014;10:135–47. 24247370 10.1038/nrrheum.2013.174

[562] Datta D, Arion D, Corradi JP, Lewis DA. Altered expression of CDC42 signaling pathway components in cortical layer 3 pyramidal cells in schizophrenia. Biol Psychiatry. 2015;78:775–85. 25981171 10.1016/j.biopsych.2015.03.030 PMC4600637

[563] Fife CM, McCarroll JA, Kavallaris M. Movers and shakers: cell cytoskeleton in cancer metastasis. Br J Pharm. 2014;171:5507–23. 10.1111/bph.12704 PMC4290699 24665826

[564] Stankiewicz TR, Linseman DA. Rho family GTPases: key players in neuronal development, neuronal survival, and neurodegeneration. Front Cell Neurosci. 2014;8:314. 25339865 10.3389/fncel.2014.00314 PMC4187614

[565] Schattgen SA, Fitzgerald KA. The PYHIN protein family as mediators of host defenses. Immunol Rev. 2011;243:109–18. 21884171 10.1111/j.1600-065X.2011.01053.x

[566] Zhao H, Gonzalezgugel E, Cheng L, Richbourgh B, Nie L, Liu C. The roles of interferon-inducible p200 family members IFI16 and p204 in innate immune responses, cell differentiation and proliferation. Genes Dis. 2015;2:46–56. 25815367 10.1016/j.gendis.2014.10.003 PMC4372153

[567] Mondini M, Vidali M, Airò P, De Andrea M, Riboldi P, Meroni PL, et al. Role of the interferon-inducible gene IFI16 in the etiopathogenesis of systemic autoimmune disorders. Ann N Y Acad Sci. 2007;1110:47–56. 17911419 10.1196/annals.1423.006

[568] Albrecht M, Choubey D, Lengauer T. The HIN domain of IFI-200 proteins consists of two OB folds. Biochem Biophys Res Commun. 2005;327:679–87. 15649401 10.1016/j.bbrc.2004.12.056

[569] Ni X, Ru H, Ma F, Zhao L, Shaw N, Feng Y, et al. New insights into the structural basis of DNA recognition by HINa and HINb domains of IFI16. J Mol Cell Biol. 2016;8:51–61. 26246511 10.1093/jmcb/mjv053

[570] Unterholzner L, Keating SE, Baran M, Horan KA, Jensen SB, Sharma S, et al. IFI16 is an innate immune sensor for intracellular DNA. Nat Immunol. 2010;11:997–1004. 20890285 10.1038/ni.1932 PMC3142795

[571] Brázda V, Coufal J, Liao JC, Arrowsmith CH. Preferential binding of IFI16 protein to cruciform structure and superhelical DNA. Biochem Biophys Res Commun. 2012;422:716–20. 22618232 10.1016/j.bbrc.2012.05.065

[572] Yan H, Dalal K, Hon BK, Youkharibache P, Lau D, Pio F. RPA nucleic acid-binding properties of IFI16-HIN200. Biochim Biophys Acta. 2008;1784:1087–97. 18472023 10.1016/j.bbapap.2008.04.004

[573] Fan X, Jiang J, Zhao D, Chen F, Ma H, Smith P, et al. Structural mechanism of DNA recognition by the p204 HIN domain. Nucleic Acids Res. 2021;49:2959–72. 33619523 10.1093/nar/gkab076 PMC7969034

[574] Aglipay JA, Lee SW, Okada S, Fujiuchi N, Ohtsuka T, Kwak JC, et al. A member of the Pyrin family, IFI16, is a novel BRCA1-associated protein involved in the p53-mediated apoptosis pathway. Oncogene. 2003;22:8931–8. 14654789 10.1038/sj.onc.1207057

[575] Barbe L, Lundberg E, Oksvold P, Stenius A, Lewin E, Björling E, et al. Toward a confocal subcellular atlas of the human proteome. Mol Cell Proteom. 2008;7:499–508. 10.1074/mcp.M700325-MCP200 18029348

[576] Berry A, Matthews L, Jangani M, Plumb J, Farrow S, Buchan N, et al. Interferon-inducible factor 16 is a novel modulator of glucocorticoid action. FASEB J. 2010;24:1700–13. 20086048 10.1096/fj.09-139998 PMC3000051

[577] Briggs LJ, Johnstone RW, Elliot RM, Xiao CY, Dawson M, Trapani JA, et al. Novel properties of the protein kinase CK2-site-regulated nuclear- localization sequence of the interferon-induced nuclear factor IFI 16. Biochem J. 2001;353:69–77. 11115400 PMC1221544

[578] Dawson MJ, Trapani JA. The interferon-inducible autoantigen, IFI 16: localization to the nucleolus and identification of a DNA-binding domain. Biochem Biophys Res Commun. 1995;214:152–62. 7545391 10.1006/bbrc.1995.2269

[579] Duan X, Ponomareva L, Veeranki S, Panchanathan R, Dickerson E, Choubey D. Differential roles for the interferon-inducible IFI16 and AIM2 innate immune sensors for cytosolic DNA in cellular senescence of human fibroblasts. Mol Cancer Res. 2011;9:589–602. 21471287 10.1158/1541-7786.MCR-10-0565 PMC3096691

[580] Veeranki S, Choubey D. Interferon-inducible p200-family protein IFI16, an innate immune sensor for cytosolic and nuclear double-stranded DNA: regulation of subcellular localization. Mol Immunol. 2012;49:567–71. 22137500 10.1016/j.molimm.2011.11.004 PMC3249514

[581] Veeranki S, Duan X, Panchanathan R, Liu H, Choubey D. IFI16 protein mediates the anti-inflammatory actions of the type-I interferons through suppression of activation of caspase-1 by inflammasomes. PLoS One. 2011;6:e27040. 22046441 10.1371/journal.pone.0027040 PMC3203938

[582] Xin H, Curry J, Johnstone RW, Nickoloff BJ, Choubey D. Role of IFI 16, a member of the interferon-inducible p200-protein family, in prostate epithelial cellular senescence. Oncogene. 2003;22:4831–40. 12894224 10.1038/sj.onc.1206754

[583] Ansari MA, Dutta S, Veettil MV, Dutta D, Iqbal J, Kumar B, et al. Herpesvirus Genome Recognition Induced Acetylation of Nuclear IFI16 Is Essential for Its Cytoplasmic Translocation, Inflammasome and IFN-beta Responses. PLoS Pathog. 2015;11:e1005019. 26134128 10.1371/journal.ppat.1005019 PMC4489722

[584] Yan Q, Zhou J, Wang Z, Ding X, Ma X, Li W, et al. NAT10-dependent N(4)-acetylcytidine modification mediates PAN RNA stability, KSHV reactivation, and IFI16-related inflammasome activation. Nat Commun. 2023;14:6327. 37816771 10.1038/s41467-023-42135-3 PMC10564894

[585] Kerur N, Veettil MV, Sharma-Walia N, Bottero V, Sadagopan S, Otageri P, et al. IFI16 acts as a nuclear pathogen sensor to induce the inflammasome in response to Kaposi Sarcoma-associated herpesvirus infection. Cell Host Microbe. 2011;9:363–75. 21575908 10.1016/j.chom.2011.04.008 PMC3113467

[586] Taabazuing CY, Griswold AR, Bachovchin DA. The NLRP1 and CARD8 inflammasomes. Immunol Rev. 2020;297:13–25. 32558991 10.1111/imr.12884 PMC7483925

[587] Johnson DC, Okondo MC, Orth EL, Rao SD, Huang HC, Ball DP, et al. DPP8/9 inhibitors activate the CARD8 inflammasome in resting lymphocytes. Cell Death Dis. 2020;11:628. 32796818 10.1038/s41419-020-02865-4 PMC7428001

[588] Linder A, Bauernfried S, Cheng Y, Albanese M, Jung C, Keppler OT, et al. CARD8 inflammasome activation triggers pyroptosis in human T cells. EMBO J. 2020;39:e105071. 32840892 10.15252/embj.2020105071 PMC7527815

[589] Sharif H, Hollingsworth LR, Griswold AR, Hsiao JC, Wang Q, Bachovchin DA, et al. Dipeptidyl peptidase 9 sets a threshold for CARD8 inflammasome formation by sequestering its active C-terminal fragment. Immunity. 2021;54:1392–1404 e10. 34019797 10.1016/j.immuni.2021.04.024 PMC8423358

[590] Chui AJ, Griswold AR, Taabazuing CY, Orth EL, Gai K, Rao SD, et al. Activation of the CARD8 Inflammasome Requires a Disordered Region. Cell Rep. 2020;33:108264. 33053349 10.1016/j.celrep.2020.108264 PMC7594595

[591] Hsiao JC, Neugroschl AR, Chui AJ, Taabazuing CY, Griswold AR, Wang Q, et al. A ubiquitin-independent proteasome pathway controls activation of the CARD8 inflammasome. J Biol Chem. 2022;298:102032. 35580636 10.1016/j.jbc.2022.102032 PMC9213247

[592] Kulsuptrakul J, Turcotte EA, Emerman M, Mitchell PS. A human-specific motif facilitates CARD8 inflammasome activation after HIV-1 infection. Elife. 2023;12:e84108. 37417868 10.7554/eLife.84108 PMC10359095

[593] Nadkarni R, Chu WC, Lee CQE, Mohamud Y, Yap L, Toh GA, et al. Viral proteases activate the CARD8 inflammasome in the human cardiovascular system. J Exp Med. 2022;219:e20212117. 36129453 10.1084/jem.20212117 PMC9499823

[594] Tsu BV, Agarwal R, Gokhale NS, Kulsuptrakul J, Ryan AP, Fay EJ, et al. Host-specific sensing of coronaviruses and picornaviruses by the CARD8 inflammasome. PLoS Biol. 2023;21:e3002144. 37289745 10.1371/journal.pbio.3002144 PMC10249858

[595] Wang Q, Gao H, Clark KM, Mugisha CS, Davis K, Tang JP, et al. CARD8 is an inflammasome sensor for HIV-1 protease activity. Science. 2021;371:eabe1707. 33542150 10.1126/science.abe1707 PMC8029496

[596] Wang Q, Clark KM, Tiwari R, Raju N, Tharp GK, Rogers J, et al. The CARD8 inflammasome dictates HIV/SIV pathogenesis and disease progression. Cell. 2024;187:1223–1237.e16. 38428396 10.1016/j.cell.2024.01.048 PMC10919936

[597] Rao SD, Chen Q, Wang Q, Orth-He EL, Saoi M, Griswold AR, et al. M24B aminopeptidase inhibitors selectively activate the CARD8 inflammasome. Nat Chem Biol. 2022;18:565–74. 35165443 10.1038/s41589-021-00964-7 PMC9179932

[598] Fontalba A, Gutiérrez O, Llorca J, Mateo I, Berciano J, Fernández-Luna JL, et al. Deficiency of CARD8 is associated with increased Alzheimer’s disease risk in women. Dement Geriatr Cogn Disord. 2008;26:247–50. 18841008 10.1159/000160956

[599] Liu J, Liu YY, Liu J, Li BZ, Cen H, Xu WD, et al. Association between CARD8 rs2043211 polymorphism and inflammatory bowel disease: a meta-analysis. Immunol Invest. 2015;44:253–64. 25564880 10.3109/08820139.2014.988721

[600] Kastbom A, Johansson M, Verma D, Söderkvist P, Rantapää-Dahlqvist S. CARD8 p.C10X polymorphism is associated with inflammatory activity in early rheumatoid arthritis. Ann Rheum Dis. 2010;69:723–6. 19443463 10.1136/ard.2008.106989

[601] Lv J, Jiang X, Zhang J, Peng X, Lin H. Combined polymorphisms in genes encoding the inflammasome components NLRP3 and CARD8 confer risk of ischemic stroke in men. J Stroke Cerebrovasc Dis. 2020;29:104874. 32689633 10.1016/j.jstrokecerebrovasdis.2020.104874

[602] Haller O, Kochs G. Human MxA protein: an interferon-induced dynamin-like GTPase with broad antiviral activity. J Interferon Cytokine Res. 2011;31:79–87. 21166595 10.1089/jir.2010.0076

[603] Kochs G, Haener M, Aebi U, Haller O. Self-assembly of human MxA GTPase into highly ordered dynamin-like oligomers. J Biol Chem. 2002;277:14172–6. 11847228 10.1074/jbc.M200244200

[604] Reichelt M, Stertz S, Krijnse-Locker J, Haller O, Kochs G. Missorting of LaCrosse virus nucleocapsid protein by the interferon-induced MxA GTPase involves smooth ER membranes. Traffic. 2004;5:772–84. 15355513 10.1111/j.1600-0854.2004.00219.x

[605] Kochs G, Haller O. Interferon-induced human MxA GTPase blocks nuclear import of Thogoto virus nucleocapsids. Proc Natl Acad Sci USA. 1999;96:2082–6. 10051598 10.1073/pnas.96.5.2082 PMC26740

[606] Lee S, Ishitsuka A, Noguchi M, Hirohama M, Fujiyasu Y, Petric PP, et al. Influenza restriction factor MxA functions as inflammasome sensor in the respiratory epithelium. Sci Immunol. 2019;4:eaau4643. 31653718 10.1126/sciimmunol.aau4643

[607] Pillai PS, Molony RD, Martinod K, Dong H, Pang IK, Tal MC, et al. Mx1 reveals innate pathways to antiviral resistance and lethal influenza disease. Science. 2016;352:463–6. 27102485 10.1126/science.aaf3926 PMC5465864

[608] Mouradov D, Sloggett C, Jorissen RN, Love CG, Li S, Burgess AW, et al. Colorectal cancer cell lines are representative models of the main molecular subtypes of primary cancer. Cancer Res. 2014;74:3238–47. 24755471 10.1158/0008-5472.CAN-14-0013

[609] Imielinski M, Berger AH, Hammerman PS, Hernandez B, Pugh TJ, Hodis E, et al. Mapping the Hallmarks of Lung Adenocarcinoma with Massively Parallel Sequencing. Cell. 2012;150:1107–20. 22980975 10.1016/j.cell.2012.08.029 PMC3557932

[610] Bell D, et al. Integrated genomic analyses of ovarian carcinoma. Nature. 2011;474:609–15. 21720365 10.1038/nature10166 PMC3163504

[611] Mushinski JF, Nguyen P, Stevens LM, Khanna C, Lee S, Chung EJ, et al. Inhibition of tumor cell motility by the interferon-inducible GTPase MxA. J Biol Chem. 2009;284:15206–14. 19297326 10.1074/jbc.M806324200 PMC2685701

